# Experimental Treatment with Favipiravir for Ebola Virus Disease (the JIKI Trial): A Historically Controlled, Single-Arm Proof-of-Concept Trial in Guinea

**DOI:** 10.1371/journal.pmed.1001967

**Published:** 2016-03-01

**Authors:** Daouda Sissoko, Cedric Laouenan, Elin Folkesson, Abdoul-Bing M’Lebing, Abdoul-Habib Beavogui, Sylvain Baize, Alseny-Modet Camara, Piet Maes, Susan Shepherd, Christine Danel, Sara Carazo, Mamoudou N. Conde, Jean-Luc Gala, Géraldine Colin, Hélène Savini, Joseph Akoi Bore, Frederic Le Marcis, Fara Raymond Koundouno, Frédéric Petitjean, Marie-Claire Lamah, Sandra Diederich, Alexis Tounkara, Geertrui Poelart, Emmanuel Berbain, Jean-Michel Dindart, Sophie Duraffour, Annabelle Lefevre, Tamba Leno, Olivier Peyrouset, Léonid Irenge, N’Famara Bangoura, Romain Palich, Julia Hinzmann, Annette Kraus, Thierno Sadou Barry, Sakoba Berette, André Bongono, Mohamed Seto Camara, Valérie Chanfreau Munoz, Lanciné Doumbouya, Souley Harouna, Patient Mumbere Kighoma, Fara Roger Koundouno, Réné Lolamou, Cécé Moriba Loua, Vincent Massala, Kinda Moumouni, Célia Provost, Nenefing Samake, Conde Sekou, Abdoulaye Soumah, Isabelle Arnould, Michel Saa Komano, Lina Gustin, Carlotta Berutto, Diarra Camara, Fodé Saydou Camara, Joliene Colpaert, Léontine Delamou, Lena Jansson, Etienne Kourouma, Maurice Loua, Kristian Malme, Emma Manfrin, André Maomou, Adele Milinouno, Sien Ombelet, Aboubacar Youla Sidiboun, Isabelle Verreckt, Pauline Yombouno, Anne Bocquin, Caroline Carbonnelle, Thierry Carmoi, Pierre Frange, Stéphane Mely, Vinh-Kim Nguyen, Delphine Pannetier, Anne-Marie Taburet, Jean-Marc Treluyer, Jacques Kolie, Raoul Moh, Minerva Cervantes Gonzalez, Eeva Kuisma, Britta Liedigk, Didier Ngabo, Martin Rudolf, Ruth Thom, Romy Kerber, Martin Gabriel, Antonino Di Caro, Roman Wölfel, Jamal Badir, Mostafa Bentahir, Yann Deccache, Catherine Dumont, Jean-François Durant, Karim El Bakkouri, Marie Gasasira Uwamahoro, Benjamin Smits, Nora Toufik, Stéphane Van Cauwenberghe, Khaled Ezzedine, Eric Dortenzio, Louis Pizarro, Aurélie Etienne, Jérémie Guedj, Alexandra Fizet, Eric Barte de Sainte Fare, Bernadette Murgue, Tuan Tran-Minh, Christophe Rapp, Pascal Piguet, Marc Poncin, Bertrand Draguez, Thierry Allaford Duverger, Solenne Barbe, Guillaume Baret, Isabelle Defourny, Miles Carroll, Hervé Raoul, Augustin Augier, Serge P. Eholie, Yazdan Yazdanpanah, Claire Levy-Marchal, Annick Antierrens, Michel Van Herp, Stephan Günther, Xavier de Lamballerie, Sakoba Keïta, France Mentre, Xavier Anglaret, Denis Malvy, JIKI Study Group

**Affiliations:** 1 Inserm, UMR 1219, Université de Bordeaux, Bordeaux, France; 2 Centre Hospitalier Universitaire de Bordeaux, Bordeaux, France; 3 Inserm, IAME, UMR 1137, Université Paris Diderot, Paris, France; 4 Assistance Publique–Hôpitaux de Paris, Hôpital Bichat Claude Bernard, Paris, France; 5 Médecins Sans Frontières Belgique, Brussels, Belgium; 6 ALIMA, Dakar, Senegal; 7 Centre de Recherche en Santé Rurale, Maférinya, Guinea; 8 Institut Pasteur, Centre International de Recherche en Infectiologie, Lyon, France; 9 Inserm, Laboratoire P4 Jean Mérieux, Lyon, France; 10 European Mobile Laboratory Project, Hamburg, Germany; 11 Rega Institute for Medical Research, Leuven, Belgium; 12 Programme PACCI, Abidjan, Côte d’Ivoire; 13 Biological Light Fieldable Laboratory for Emergencies (B-LiFE)/Belgian First Aid and Support (B-FAST), Brussels, Belgium; 14 Cliniques Universitaires Saint-Luc, Brussels, Belgium; 15 Université Catholique de Louvain, Louvain-la-Neuve, Belgium; 16 Belgian Ministry of Defense, Brussels, Belgium; 17 Croix Rouge Française, Paris, France; 18 Service de Santé des Armées, Paris, France; 19 Institut National de Santé Publique, Conakry, Guinea; 20 Laboratoire des Fièvres Hémorragiques en Guinée, Université Gamal Abdel Nasser de Conakry, Conakry, Guinea; 21 Ecole Normale Supérieure, Lyon, France; 22 Friedrich Loeffler Institute–Federal Research Institute for Animal Health, Greifswald, Germany; 23 Robert Koch Institute, Berlin, Germany; 24 Public Health Agency of Sweden, Solna, Sweden; 25 Assistance Publique–Hôpitaux de Paris, Hôpital Necker–Enfants Malades, Paris, France; 26 Université de Montréal, Montréal, Canada; 27 Assistance Publique–Hôpitaux de Paris, Hôpital Bicêtre, Paris, France; 28 Public Health England, Porton Down, United Kingdom; 29 Bernhard Nocht Institute for Tropical Medicine, Hamburg, Germany; 30 National Institute for Infectious Diseases “L. Spallanzani,” Rome, Italy; 31 Bundeswehr Institute of Microbiology, Munich, Germany; 32 Solidarité Thérapeutique et Initiatives pour la Santé (Solthis), Paris, France; 33 Inserm, Paris, France; 34 Southampton General Hospital, University of Southampton, Southampton, United Kingdom; 35 Centre Hospitalier Universitaire de Treichville, Abidjan, Côte d’Ivoire; 36 Université Aix Marseille, Institut de Recherche pour le Développement, École des Hautes Études en Santé Publique, EPV, Marseille, France; 37 Cellule de Coordination Nationale de Lutte contre la Maladie à Virus Ebola, Conakry, Guinea; Harvard School of Public Health, UNITED STATES

## Abstract

**Background:**

Ebola virus disease (EVD) is a highly lethal condition for which no specific treatment has proven efficacy. In September 2014, while the Ebola outbreak was at its peak, the World Health Organization released a short list of drugs suitable for EVD research. Favipiravir, an antiviral developed for the treatment of severe influenza, was one of these. In late 2014, the conditions for starting a randomized Ebola trial were not fulfilled for two reasons. One was the perception that, given the high number of patients presenting simultaneously and the very high mortality rate of the disease, it was ethically unacceptable to allocate patients from within the same family or village to receive or not receive an experimental drug, using a randomization process impossible to understand by very sick patients. The other was that, in the context of rumors and distrust of Ebola treatment centers, using a randomized design at the outset might lead even more patients to refuse to seek care.

Therefore, we chose to conduct a multicenter non-randomized trial, in which all patients would receive favipiravir along with standardized care. The objectives of the trial were to test the feasibility and acceptability of an emergency trial in the context of a large Ebola outbreak, and to collect data on the safety and effectiveness of favipiravir in reducing mortality and viral load in patients with EVD. The trial was not aimed at directly informing future guidelines on Ebola treatment but at quickly gathering standardized preliminary data to optimize the design of future studies.

**Methods and Findings:**

Inclusion criteria were positive Ebola virus reverse transcription PCR (RT-PCR) test, age ≥ 1 y, weight ≥ 10 kg, ability to take oral drugs, and informed consent. All participants received oral favipiravir (day 0: 6,000 mg; day 1 to day 9: 2,400 mg/d). Semi-quantitative Ebola virus RT-PCR (results expressed in “cycle threshold” [Ct]) and biochemistry tests were performed at day 0, day 2, day 4, end of symptoms, day 14, and day 30. Frozen samples were shipped to a reference biosafety level 4 laboratory for RNA viral load measurement using a quantitative reference technique (genome copies/milliliter). Outcomes were mortality, viral load evolution, and adverse events. The analysis was stratified by age and Ct value. A “target value” of mortality was defined a priori for each stratum, to guide the interpretation of interim and final analysis.

Between 17 December 2014 and 8 April 2015, 126 patients were included, of whom 111 were analyzed (adults and adolescents, ≥13 y, *n* = 99; young children, ≤6 y, *n* = 12). Here we present the results obtained in the 99 adults and adolescents. Of these, 55 had a baseline Ct value ≥ 20 (Group A Ct ≥ 20), and 44 had a baseline Ct value < 20 (Group A Ct < 20). Ct values and RNA viral loads were well correlated, with Ct = 20 corresponding to RNA viral load = 7.7 log_10_ genome copies/ml. Mortality was 20% (95% CI 11.6%–32.4%) in Group A Ct ≥ 20 and 91% (95% CI 78.8%–91.1%) in Group A Ct < 20. Both mortality 95% CIs included the predefined target value (30% and 85%, respectively). Baseline serum creatinine was ≥110 μmol/l in 48% of patients in Group A Ct ≥ 20 (≥300 μmol/l in 14%) and in 90% of patients in Group A Ct < 20 (≥300 μmol/l in 44%). In Group A Ct ≥ 20, 17% of patients with baseline creatinine ≥110 μmol/l died, versus 97% in Group A Ct < 20. In patients who survived, the mean decrease in viral load was 0.33 log_10_ copies/ml per day of follow-up. RNA viral load values and mortality were not significantly different between adults starting favipiravir within <72 h of symptoms compared to others. Favipiravir was well tolerated.

**Conclusions:**

In the context of an outbreak at its peak, with crowded care centers, randomizing patients to receive either standard care or standard care plus an experimental drug was not felt to be appropriate. We did a non-randomized trial. This trial reaches nuanced conclusions. On the one hand, we do not conclude on the efficacy of the drug, and our conclusions on tolerance, although encouraging, are not as firm as they could have been if we had used randomization. On the other hand, we learned about how to quickly set up and run an Ebola trial, in close relationship with the community and non-governmental organizations; we integrated research into care so that it improved care; and we generated knowledge on EVD that is useful to further research. Our data illustrate the frequency of renal dysfunction and the powerful prognostic value of low Ct values. They suggest that drug trials in EVD should systematically stratify analyses by baseline Ct value, as a surrogate of viral load. They also suggest that favipiravir monotherapy merits further study in patients with medium to high viremia, but not in those with very high viremia.

**Trial registration:**

ClinicalTrials.gov NCT02329054

## Abstract 2

In the context the recent Ebola outbreak, Xavier Anglaret and colleagues test an experimental treatment, favipiravir, for Ebola virus disease in a multicenter non-randomized trial.

## Editors' Summary

**Background:**

In 2014 and 2015, an Ebola virus outbreak larger than any known before occurred in West Africa. Ebola virus disease (EVD) is highly contagious, and many infected people die. Central to the emergency response to the recent outbreak were local Ebola treatment centers where patients were diagnosed, were isolated, and received supportive care. With thousands of patients dying and many health workers contracting the disease, fear was ubiquitous and distrust abundant. While conducting research in this environment was extremely challenging, the urgent need for treatments and the opportunity to conduct studies that could bring such treatments closer to reality was also recognized. In September 2014, WHO released a short list of existing drugs that were candidates for clinical trials among patients infected in the outbreak. Favipiravir, an antiviral drug developed in Japan for patients with severe influenza, was on the list.

**Why Was This Study Done?:**

Because of the urgent need to find drugs that could reduce deaths caused by Ebola, the researchers decided to conduct a clinical trial using favipiravir in patients with EVD in Guinea. In view of the circumstances, they decided against a randomized controlled trial and instead designed a study where all participants would receive the same treatment. In randomized controlled trials only some participants receive the treatment in addition to standard care, while others serve as a control group and receive standard care only, or standard care plus a placebo. Such studies allow stronger conclusions to be drawn about whether a treatment is safe and whether it works or not. The researchers had two main reasons for this decision. First, patients from the same family or village often sought EVD treatment at the same time, and the researchers felt that it was ethically unacceptable to randomize such groups, with only some of them receiving the experimental drug. Second, the strict isolation procedures imposed to interrupt virus transmission had intensified fear in affected communities and fueled rumors of illicit drug experimentation and organ theft at the treatment centers. In this context, the researchers worried that a randomized study might increase distrust among the community and the reluctance of patients to seek care.

Rather than seeking definitive answers about the safety and efficacy of favipiravir in patients with EVD, the objectives of the study as it was designed were to test the feasibility and acceptability of an emergency trial in the context of a large Ebola outbreak and to learn lessons from the experience. In addition, the researchers planned to collect data on the safety and effectiveness of favipiravir in reducing mortality and viral load in patients with EVD in the hope that their preliminary findings could improve the design of subsequent trials and the chance to provide conclusive answers.

**What Did the Researchers Do and Find?:**

After 13 weeks of preparation, the trial took place from December 2014 to April 2015 at four separate Ebola treatment centers, three in rural areas and one in an urban setting. In addition to standard care (which included rehydration, antimalarial and antibacterial therapies, and medication to reduce fever, pain, and nausea), all participants were given favipiravir by mouth for ten days, at doses substantially higher than those recommended for patients with influenza. Outcomes measured were mortality, viral load changes over time (based on blood samples), and adverse events.

EVD was confirmed with an assay that used patient blood and provided an estimate of the viral load, that is, of how much virus the blood contained. Because viral load was known to influence the course of EVD, the researchers analyzed the participants in two groups, namely, those with a viral load estimate above a certain threshold and those with viral load estimate below the threshold. They also used existing data from Guinean patients diagnosed with Ebola earlier in the outbreak who had received only standard care and calculated an expected mortality rate for patients above and below the viral load threshold.

The researchers were able to enroll 126 participants in the trial. Of these, 111 were included in the final analysis. Of 99 adult and adolescent participants 13 years and older, 55 were in the lower viral load group and 44 in the higher viral load group. Mortality was 20% in the former and 91% in the latter. Neither mortality rate was significantly different from that of earlier patients who had received only standard care. The researchers also found that favipiravir was well tolerated. None of the patients stopped the course of treatment, vomiting following drug intake was rare, and no severe adverse events were attributed to the drug. The researchers did not see a difference in mortality between patients who reported onset of symptoms less than three days before the start of treatment and those whose symptoms had started more than three days the start of treatment.

**What Do these Findings Mean?:**

The report shows that it is possible to conduct an emergency trial during an outbreak in a low-resource setting. In fact, at the time of its acceptance, this paper reported on an Ebola treatment trial larger than any other yet published. The experience described should be useful for similar undertakings in the future. The following conditions contributed to the success of the trial: close collaboration between researchers, local health officials, and affected communities on one hand, and flexibility in design, conduct, and analysis based on close monitoring and interim assessments on the other. Besides using interim results to influence the conduct and analysis of their own trial, the researchers also shared these results with the scientific community in real time, and this feedback influenced other research during the outbreak.

The trial could not answer definitively whether favipiravir treatment was safe or reduced mortality in patients with EVD. The results suggest that the drug is unlikely to be beneficial for patients with very high viral loads, at least when given by itself. They also suggest that favipiravir is safe in patients with lower viral loads, and that in such patients additional efficacy studies are warranted. Intermediate analysis of various measurements in trial participants showed that the estimate of viral load from the field EVD diagnosis test is a good proxy for the actual viral load (determined after the samples were shipped to and analyzed in a reference laboratory in France) and suitable as a surrogate marker. The results also confirm that viral load is a strong predictor of mortality.

**Additional Information:**

This list of resources contains links that can be accessed when viewing the PDF on a device or via the online version of the article at http://dx.doi.org/10.1371/journal.pmed.1001967.

- The World Health Organization has pages on Ebola virus disease, trials of Ebola treatments and vaccines, and the current update of the list of suitable drugs for testing or use in patients infected with Ebola (originally compiled in September 2014)
- US Centers for Disease Control and Prevention has information on the Ebola outbreak in West Africa
- The European Centre for Disease Prevention and Control also has information on the Ebola outbreak in West Africa

## Introduction

Since December 2013, a large outbreak of Ebola virus disease (EVD) has occurred, principally affecting Guinea, Liberia, and Sierra Leone [1–3]. This has been the largest and deadliest EVD outbreak ever to be reported.

Once symptomatic, the disease rapidly moves toward a systemic inflammatory response with immune suppression and multi-organ failure, resulting in very high mortality rates [4,5]. In the absence of effective specific treatments, care is primarily aimed at managing complications [6–10]. In West Africa, care of patients with EVD is provided at clinics with limited facilities compared to those available in -higher income countries.

In September 2014, the World Health Organization (WHO) launched a fast-track process to identify potential anti-Ebola drugs, and identified four classes of products, namely, immunomodulators, immunoglobulins, small inhibitory RNA, and antivirals [11]. Three criteria were established for a drug to be acceptable as a candidate for clinical trials, namely, availability of safety data in humans, evidence for in vivo efficacy against Ebola virus (EBOV) from preclinical studies, and sufficient drug supply. Favipiravir, an RNA polymerase inhibitor, was the only antiviral to meet all three criteria. The drug, originally developed and approved in Japan for the treatment of severe influenza, had documented activity against EBOV in mice [12–15]. Tolerance had been demonstrated to be good in more than 2,000 healthy humans participating in phase I trials or humans with influenza participating in phase II or III trials of treatment of influenza with favipiravir (Carol Epstein, personal communication) [16]. The drug was readily available and had already been given through compassionate use to several health workers with EVD evacuated from West Africa to Europe [10].

In September 2014, our group decided to initiate a proof-of-concept trial of favipiravir in patients with EVD in Guinea. The objectives of the trial were to test the feasibility and acceptability of an emergency trial in the context of a large Ebola outbreak and to collect preliminary data on the safety and effectiveness of favipiravir in reducing mortality and viral load in patients with EVD.

## Methods

### Pre-trial Assumptions and Basic Requirements

This trial had to be thought out in highly unusual conditions. When designing the trial, we made the following basic assumptions.

#### Staff and patient safety issues

The trial was to be conducted in rural field centers integrated into the community and with little if any experience of conducting clinical trials. Particular attention had to be paid to the risks for healthcare personnel and patients. Blood collection and sample manipulation had to be restricted to the minimum. Data collection had to be simple, with no circulation of material from the inside towards the outside of the isolation zone. Any decisions regarding modification of current care practices had to be made because they were considered necessary to improve care, not just for the sake of the trial. Due to the limited availability of clinical and paraclinical resources for monitoring toxicity, particular attention had to be paid to mortality. Therefore, we decided that mortality should be reported to a data safety monitoring board (DSMB) on a daily basis and that intermediate analyses should be performed after inclusion of every 20 adult patients (or every ten children), with the trial to be stopped in case of futility or unanticipated high mortality.

#### The importance of early treatment

The efficacy of an antiviral treatment for acute viral diseases is likely to be optimal within the first hours following infection, when a high ratio of antiviral drug level to viral particles can still be achieved [15,17]. Thus, in order to have the highest chance to detect the potential efficacy of favipiravir, we would have preferred the trial to be conducted in patients starting treatment in the very early stages of the disease course. However, no historical data were available to determine empirically the optimal cutoff value for the time since first infection that would discriminate between effective and ineffective anti-EBOV drug treatment. Moreover, the only way of determining the disease duration was to use as a proxy the time since first symptoms as reported by the patient, which was a subjective variable that may not be reliable. Therefore, we decided arbitrarily that all individuals with no contraindication to favipiravir had to be proposed treatment, regardless of symptom duration, but that the main analysis would be conducted in patients starting treatment within 3 d of symptom onset.

#### The need for a high dose

For the treatment of influenza, favipiravir is given orally for 5 d, at the dose of 1,600 mg twice a day on the first day and of 600 mg twice a day for the subsequent 4 d [13,16]. Because higher concentrations of favipiravir were required to inhibit EBOV than H1N1 in preclinical studies, we knew that the dose for Ebola had to be higher than that used for influenza [14,15]. However, because no dose-finding trial had previously been conducted in EVD, we had to find a way to estimate the appropriate anti-Ebola dose. We used in vivo and in vitro data on favipiravir efficacy against EBOV and on favipiravir pharmacokinetics in uninfected mice and humans to estimate the target plasma concentration in humans with EVD, and then to model the dose to achieve the target [18]. We calculated the target time-weighted average plasma concentration (*C*_ave_u_) to be 52 μg/ml. Using this model, we estimated that the dose required to achieve this concentration in adults would be 6,000 mg on day 0, followed by 2,400 mg/d from day 1 to day 9. Doses in children were derived from adult doses and adapted for body weight [19]. Since the duration of the EBOV viremic phase had previously been estimated to be 10 d [20–22], and there may be a risk of a viral rebound due to EVD-related immunosuppression if an effective antiviral treatment is stopped too early, we decided empirically that the duration of treatment would be 10 d.

#### The issue of children

Previous clinical data on favipiravir for the treatment of influenza have been collected exclusively in adults. However, given the high EVD mortality rate and the good tolerance profile of the drug, we considered it unethical to restrict access to the drug to parents while their children would receive standard care only [23]. Therefore, we asked pediatricians to assess potential safety issues in children. The experts concluded that maturation of both renal function and the enzymes involved in the metabolism of favipiravir was achieved at the age of 12 mo, whereas favipiravir disposition would be impossible to predict in infants below this age. We thus decided to include all children ≥12 mo of age.

#### The choice of trial design

The conditions for rapidly putting together a trial were difficult for six reasons. First, there was an urgent need to identify drugs capable of decreasing EVD mortality, but none of the potential anti-Ebola drugs, including favipiravir, had undergone the full development program that usually precedes efficacy trials. Second, limited data were available on the evolution of biological parameters during the course of the disease [7,24,25]. Therefore, there was little evidence to base a trial design on. Third, care provision was difficult to organize because of the anticipated workload and the danger to non-governmental organization (NGO) personnel. For example, certain NGOs had decided not to administer treatments by the intravenous route if the number of patients to treat was high. In many treatment centers, medical follow-up was exclusively clinical and no biological monitoring was performed [26]. Fourth, the course of the current outbreak was unpredictable, which made it impossible to predict the recruitment capacity for a trial. Fifth, EVD often strikes in clusters, with many patients from the same family presenting at a given treatment center at the same time. Both the NGOs engaged in Ebola containment and treatment in Guinea and the JIKI trial investigators felt that, given the very high mortality rate of the disease, it was ethically unacceptable to randomize patients from within the same family or village, who appear together to seek care, to receive or not receive an experimental drug. Finally, EVD is a highly transmissible disease needing strict isolation measures that compound the fear generated by this disease in the communities it affects. This had led to frequent tensions in the community, including physical violence and rumors of illicit drug experimentation and organ stealing at Ebola treatment centers [27]. This also commonly led outreach teams to face the difficult situation of patients refusing to be transferred to an Ebola treatment center [28]. In this context, there was the fear that a randomized research design might lead the community to become even more distrustful, and patients more reluctant to seek care [29]. The first four reasons invited us to rapidly gather standardized preliminary data to guide further research. The two latter reasons made us decide that the conditions for running a randomized trial were not fulfilled.

Therefore, we chose to conduct a proof-of-concept trial, in which all patients would receive favipiravir as well as standardized care including intravenous fluid regulation, and in which virological and biochemistry parameters would be systematically monitored during treatment. The trial was not aimed at directly informing future guidelines on Ebola treatment but at quickly gathering standardized preliminary data to optimize the design of future studies.

### Design

The JIKI trial was a multicenter proof-of-concept non-comparative trial conducted in four Ebola treatment centers in Guinea.

### Endpoints

In a proof-of-concept trial, primary endpoints are usually pharmacokinetics, tolerance, and biological efficacy. Because of the particularly high case fatality rate in this disease, we chose mortality as the primary endpoint. We hypothesized that all deaths would occur within 14 d. Therefore, the prespecified primary endpoint was mortality within 14 d. Secondary endpoints were evolution of EBOV plasma RNA, occurrence of grade 3 or 4 clinical or biological adverse events, criteria for discharge, evolution of infectious loads, viral micro-diversity, and plasma trough concentrations of favipiravir. Criteria for discharge were the absence of fever and significant symptoms for four consecutive days, ability to feed and walk independently, and two consecutive negative blood EBOV reverse transcription PCR (RT-PCR) tests.

### Settings

**Fig 1:**
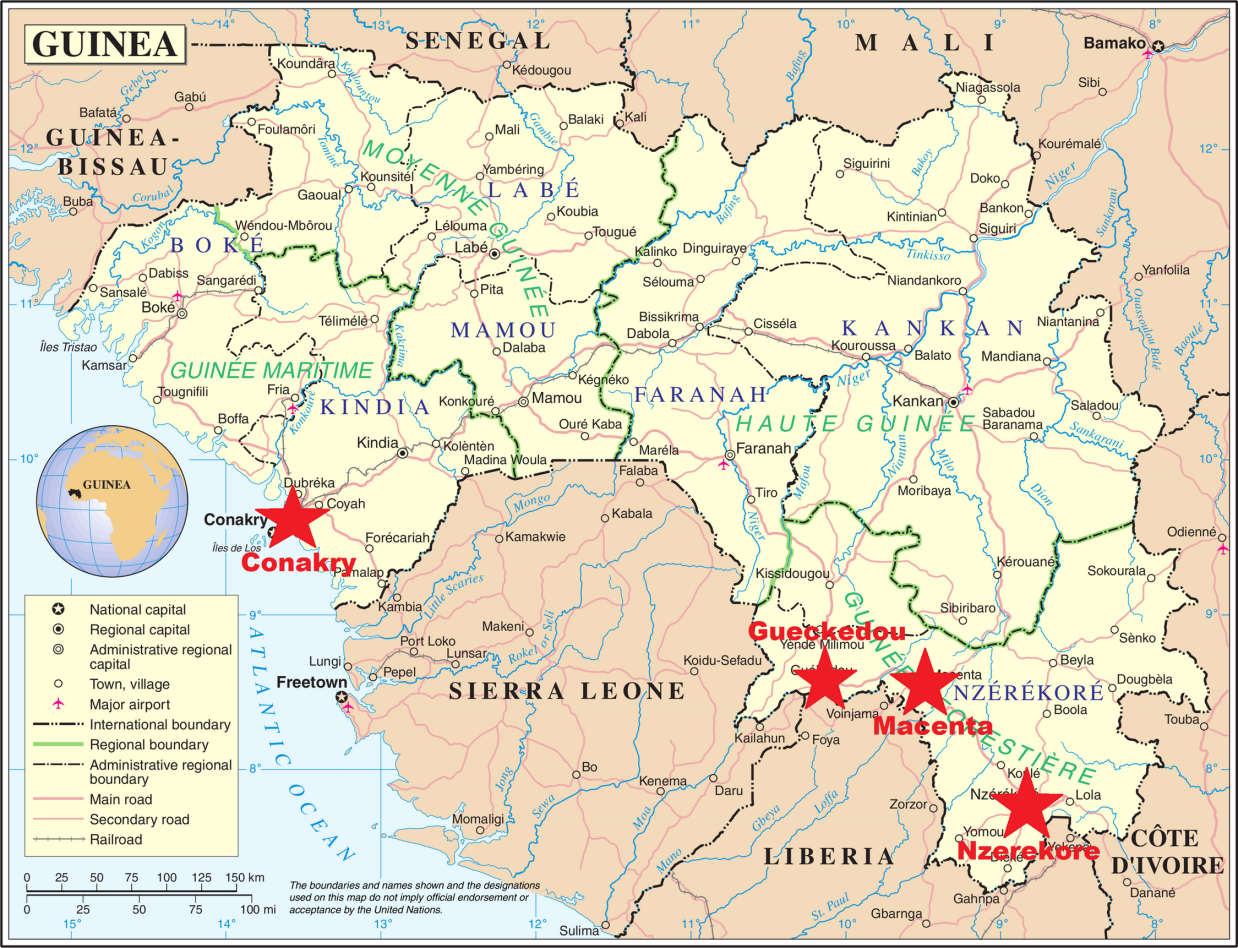
JIKI trial settings. The red stars indicate the four Ebola treatment centers that participated in the JIKI trial. Three were in the southern Guinean highlands (Gueckedou, Macenta, Nzerekore), and one was in coastal Guinea (Conakry). Source: Adapted from a Wikipedia map, derived from a United Nations map; public domain.

Three Ebola treatment centers were identified before initiation of the trial, all located in rural forested Guinea: one in Gueckedou, run by Médecins sans Frontières (MSF), one in Nzerekore, run by the Alliance for International Medical Action, and one in Macenta, run by the French Red Cross. After the trial was started, a fourth center was added, located at the capital’s international airport (Conakry) and run by the French military health service. This last center was specifically dedicated to the care of healthcare workers (Fig 1).

### Eligibility Criteria

All patients admitted to one of the four treatment centers during the study period were assessed for eligibility criteria. The inclusion criteria were age ≥ 1 y and body weight ≥ 10 kg, EVD confirmed by a positive RT-PCR test for EBOV RNA, and oral or signed informed consent. Exclusion criteria were pregnancy and inability to take the drug (encephalopathy or severe vomiting).

### Trial Treatment

All participants received standard care and favipiravir. Standard care included oral or intravenous rehydration, electrolyte correction, empiric antimalarial and antibacterial therapies, analgesics, and antiemetic drugs [6–10].

Favipiravir (Toyama Chemical, 200-mg tablets that can be crushed and mixed with liquid) was given orally. The treatment started as soon as consent was obtained (day 0) and was administered for 10 d. The adult dose was 6,000 mg on day 0 (first dose: 2,400 mg; second dose [8 h after the first dose]: 2,400 mg; third dose [8 h after the second dose]: 1,200 mg) and 2,400 mg (1,200 mg twice a day) on day 1 to day 9. For children, the dose was adapted according to body weight [19].

Patients remained hospitalized at the treatment center until day 14 or until they reached the criteria for discharge (see “Endpoints” above) if this occurred after day 14. After discharge, all patients were asked to return at day 30 for an end-of-study visit.

### Laboratory Tests

Blood samples were taken in EDTA and heparin tubes at day 0, day 2, day 4, end of symptoms, day 14, and day 30. Samples were immediately decanted. EDTA tubes were used for immediate determination of viremia, and heparin tubes for standard biochemical tests. The remaining serum was divided into aliquots and frozen at −20°C.

Immediate tests were performed at onsite laboratories in Gueckedou (European Mobile Laboratory Project), Macenta (Pasteur Institute), Nzerekore (Biological Light Fieldable Laboratory for Emergencies/Belgian First Aid and Support), and Conakry (French military health service).

Initial diagnosis of EVD was made using a semi-quantitative RT-PCR assay (RealStar Filovirus Screen RT-PCR Kit 1.0, Altona Diagnostics). The results were expressed in terms of “cycle threshold” (Ct), whose value is inversely proportional to viral load. The Ct cutoff value for positivity was <40. Biochemical assays were performed using either the Piccolo Xpress (Abaxis) or the i-STAT (Abbott Laboratories) point-of-care system. In all four centers, systematic biochemical tests included blood urea nitrogen, creatinine, sodium, potassium, and glucose. In three of the four centers (Gueckedou, Macenta, and Conakry), serum aspartate aminotransferase (AST), alanine aminotransferase (ALT), total bilirubin, amylase, albumin, creatine phosphokinase (CK), and C-reactive protein (CRP) were also assayed. During the trial, viral load (via Ct) and blood biochemistry results were provided to clinical teams in real time.

Frozen samples were then shipped to the Inserm Jean Mérieux BSL-4 Laboratory in Lyon. Viral RNA was extracted from 100 μl of plasma using a QIACube instrument and the QIAamp viral RNA isolation kit (Qiagen). Viral RNA was reverse transcribed, and the resulting DNA amplified in duplicates using a LightCycler 480 (Roche Diagnostics) with the OneStep RT-PCR Kit (Qiagen) and the primers and probe described by Huang et al. [30]. The absolute number of viral RNA copies was determined using a 655b RNA fragment synthesized with the RiboProbe Combination System–SP6/T7 (Promega). The detection limit of the assay was 2,500 RNA copies/ml of plasma.

### Ethics

Three ethics committees were approached, namely, the institutional review board of the Institut National de la Santé et de la Recherché Médicale (Inserm, France), the Médecins Sans Frontières International Ethics Committee, and the Guinean Comité National d’Éthique pour la Recherche en Santé. All three committees commented on the protocol and approved the final version and further amendments. Even though not asked for formal approval, because it was neither the sponsor nor investigator of the study, the WHO Ethics Research Committee received the trial protocol and provided important advice that helped improve it.

Informed consent forms were signed by the patients themselves for adults, or by the parents or guardians for children. When an adult gave oral consent, the form was signed by both the social worker and the physician who provided the pre- and post-test counseling and informed the patient about the trial [31]. The three institutional review boards approved the use of oral consent. The trial information sheets and consent forms are presented in S3 Text.

### Statistical Analysis

The statistical analysis plan evolved between the start of the trial in December 2014 and the interim analysis of the data in January 2015. This section describes these changes in the order in which they were made.

#### Initial principles

The study protocol established two principles. First, even if all patients received the same treatment and were followed under the same procedures, for the data analysis, they needed to be stratified into subgroups relating to variables that were expected to influence the effectiveness of favipiravir. This enabled a reduction in the number of participants to be included and thus a more rapid preliminary evaluation.

In the initial version of the protocol, this stratification was performed with respect to two variables. The first was age, with patients being divided into an adult subgroup, ≥18 y of age, and a children and adolescents subgroup, <18 y. The second stratification variable was the time since first symptoms, as reported by the patient, using a cutoff threshold of 72 h. This stratification was justified by the study hypothesis that favipiravir would be more effective in patients treated early in the disease course. Since the number of children and adolescents was expected to be low, stratification by time since first symptoms was not applied to this age group. Therefore, three analysis subgroups were considered: adults who started favipiravir within 72 h of first symptoms (Group A <72 h), adults who started favipiravir ≥72 h after symptom onset (Group A ≥72 h), and children and adolescents (Group C).

Second, data-derived mortality thresholds (“target values”) were defined a priori for each analysis subgroup. These target values could be used to decide whether to stop or continue the trial, and to guide analysis and interpretation of the data. If the interim analyses showed that the trial would have no chance of demonstrating a mortality rate significantly lower than the target value using the Whitehead triangular test [32], the trial would be stopped for futility [33]. The protocol specified that the target values would be chosen on the basis of the best available historical data (see below).

Before we found any historical database, we estimated that 60 participants in a given group would provide a power at least equal to 89% to conclude that mortality in the group was significantly lower than any target value if the trial mortality was 20% lower than the target value (S1 Table). For example, If the target value in a group was set to 60%, 60 participants in this group would provide 89% power to show that trial mortality was significantly lower than the target value if trial mortality in this group was ≤40%. Because the efficacy of favipiravir was expected to be greater in Group A <72 h, we initially decided that recruitment would continue in all groups until 60 patients had been recruited into Group A <72 h, unless early termination was recommended by the DSMB.

#### Historical data

In the middle of December 2014, before the first patient was enrolled in the study, we received a database including information on age, sex, date of first symptoms, outcome (died or recovered), and the date that the outcome was documented for all patients hospitalized in the Ebola treatment centers managed by MSF in forested Guinea since the beginning of the outbreak. Using this database, we analyzed the data for the 540 patients hospitalized between 15 September and 15 December 2014, the 3 mo preceding the start of the JIKI study. The characteristics of these patients are presented in S2 Table. In this database, the overall case fatality rate was 58%, there was no indication that the case fatality rate evolved over the 3-mo evaluation period (S1 Fig), and mortality was not significantly different between adults hospitalized <72 h or ≥72 h after first symptoms. Based on these findings, we set the target value for the JIKI trial analysis to 55% in all groups.

At the beginning of January 2015, a fortnight after starting the study, we obtained additional biological data on the 540 patients in the database and, notably, information on viral load expressed as EBOV RT-PCR Ct. We therefore combined the two databases and performed a multivariable regression analysis of variables associated with mortality [34]. We found that Ct value was independently and strongly associated with mortality, with a cutoff at Ct = 20 and an adjusted odds ratio of mortality of 11.1 (95% CI 7.2–17.1) in patients with baseline Ct < 20 compared to those with baseline Ct ≥ 20. Age was also associated with mortality, with two cutoffs, one at 6 y and one at 30 y. These cutoffs were determined by maximizing the Youden’s index (S3 Table; S2 Fig) [35].

#### Revised stratification and target values

The finding from the multivariable analysis of the historical database was considered as important new information, having a bearing on the conduct or interpretation of the study. It was presented to the trial’s scientific advisory board and DSMB on 14 January 2015. In consequence, it was decided that three new groups, based on age and baseline Ct value, would be considered in the final analysis, and a protocol amendment enshrining this decision was implemented. For patients >6 y of age, the amendment established two groups, the first with a baseline Ct value ≥ 20 (Group A Ct ≥ 20) and the second with a baseline Ct value < 20 (Group A Ct < 20). For young children (≤6 y of age), for whom the sample size was expected to be too small to allow stratification, the group included all patients irrespective of baseline Ct value (Group YC). In the historical database, the case fatality rate was 69% for patients ≤6 y, 30% for patients >6 y with baseline Ct ≥ 20, and 85% for patients >6 y with baseline Ct < 20 (S2 Table). Based on these findings, we set the JIKI trial target mortality threshold to 70% for Group YC, 30% for Group A Ct ≥ 20, and 85% for Group A Ct < 20.

An interim analysis in the first 80 participants enrolled in the JIKI trial confirmed that there was a striking difference in mortality between Group A Ct ≥ 20 and Group A Ct < 20, and suggested that the final results of the trial would be very likely to show that favipiravir would have no efficacy in Group A Ct < 20. In addition, the analysis showed that Group A Ct ≥ 20 and Group A Ct < 20 were strikingly different in terms of the frequency, seriousness, and reversibility of kidney failure. These interim results thus clearly suggested that trials of antiviral drugs in EVD should be stratified by Ct value and that trials of antiviral monotherapy should primarily target patients with a Ct ≥ 20. Since the JIKI trial was the only ongoing anti-Ebola trial at that time, and because these findings were considered to provide an important change in paradigm for designing antiviral drug trials in EVD, the investigators, together with the steering committee and the DSMB, made the decision to communicate the interim results while the trial was ongoing. The results were thus presented as a late-breaker oral presentation at the 22nd Conference on Retroviruses and Opportunistic Infections (Seattle, Washington, US; 23–26 February 2015; abstract 103-ALB) [36].

In this paper, we report the final results of the JIKI trial using the revised stratification, and expand and complement the messages delivered in February 2015.

#### Other statistical methods

Mortality rates are given with their 95% confidence interval, as estimated by using the Wilson method [33].

Among patients who survived, we analyzed the evolution of log_10_ viral load data using a linear mixed effect model assuming additive error. The model had two parameters, one for the baseline log_10_ viral load and one for the slope of evolution of log_10_ viral load under treatment, both with additive random effects. Parameters were estimated using the stochastic approximation expectation maximization (SAEM) algorithm implemented in Monolix 4.3 (Lixoft). For each parameter we report the estimated mean and standard deviation (SD) of inter-individual variability [37].

### Trial Implementation

**Fig 2:**
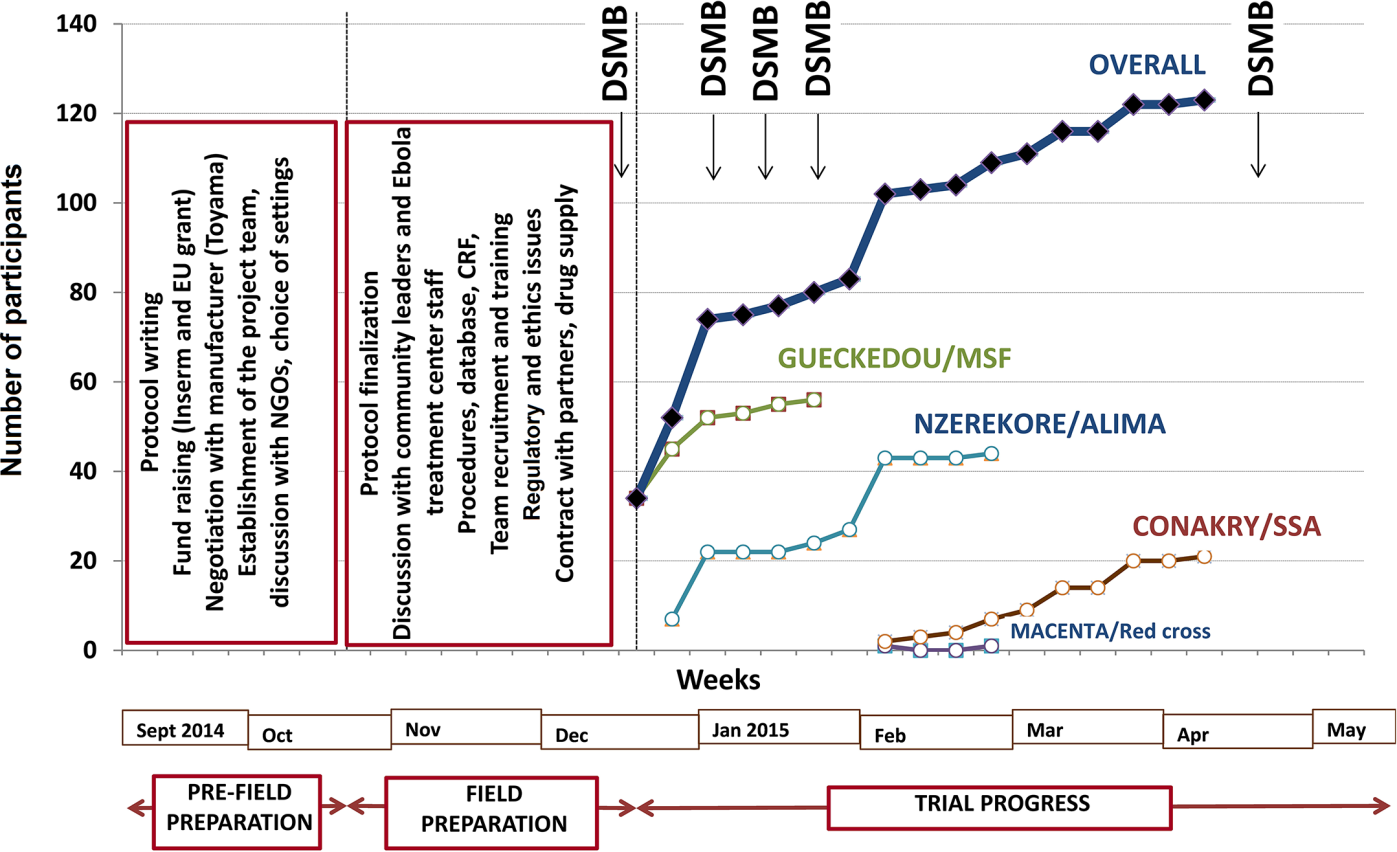
JIKI trial progress. ALIMA, Alliance for International Medical Action; CRF, Croix Rouge Française (French Red Cross); EU, European Union; Inserm, Institut National de la Santé et de la Recherche Médicale; SSA, Service de Santé des Armées (French military health service).

The decision to perform the trial was made on 7 September 2014. It took us 13 wk to open the first site and start recruitment. This time was dedicated to building the trial consortium; writing the protocol; raising funds; negotiating with the manufacturers of favipiravir; identifying the trial sites; seeking approval from the three ethics committees; preparing the trial case report form, procedures, and database; organizing the drug shipment; and defining and agreeing on clinical and laboratory procedures, in addition to preparing the trial in the field (Fig 2).

The first field action consisted of an intensive phase of communication with the community, both outside (community leaders) and inside (staff) the Ebola treatment centers, to present the trial and seek advice on procedures. Outside the treatment centers, we established a community monitoring board for the trial, including local political and community leaders. This committee was informed about the goals and procedures of the trial before enrollment started and was provided with regular updates on study progress once the trial had started. The committee was mandated to provide feedback from the community on perceptions of the study and to advise investigators on practical, social, and ethical issues arising from the conduct of the study, such as consent for participating children whose parents were not contactable. Within the treatment centers, we met all team members, including non-medical and paramedical personnel, during 19 meetings with a total of 326 individuals. The second action consisted of working out all aspects relating to informed consent, adherence, and communication with patients and family with the treatment center social workers. Further actions included logistical arrangements, recruitment of staff to compensate for the projected increased workload due to the trial (counseling, collecting information, obtaining consent, drug preparation and delivery, blood sample collection), and training sessions for all personnel involved with any trial procedure. The last pre-inclusion step consisted of two formal launch meetings with the DSMB and with the community monitoring board.

## Results

### Trial Progress

The trial sites were opened sequentially. Recruitment started on 17 December 2014 in Gueckedou, 26 December 2014 in Nzerekore, 1 February 2015 in Conakry, and 3 February 2015 in Macenta. As shown in Fig 2, recruitment had two rapid phases, one in mid-December in Gueckedou and one in late January in Nzerekore, during which 36 and 17 patients were recruited in 4 d and 2 d, respectively [38]. During the rest of the trial, recruitment was slow, with no further cluster effects. The three forested Guinea clinics enrolled patients until they were eventually closed because of the end of the outbreak in the region. On 26 May 2015, the only trial center remaining open, the Conakry center, had not admitted any patients since 8 April 2015. After seeking advice from the trial DSMB and scientific advisory board, the investigators decided to close the trial and analyze the data.

### Participants

**Fig 3:**
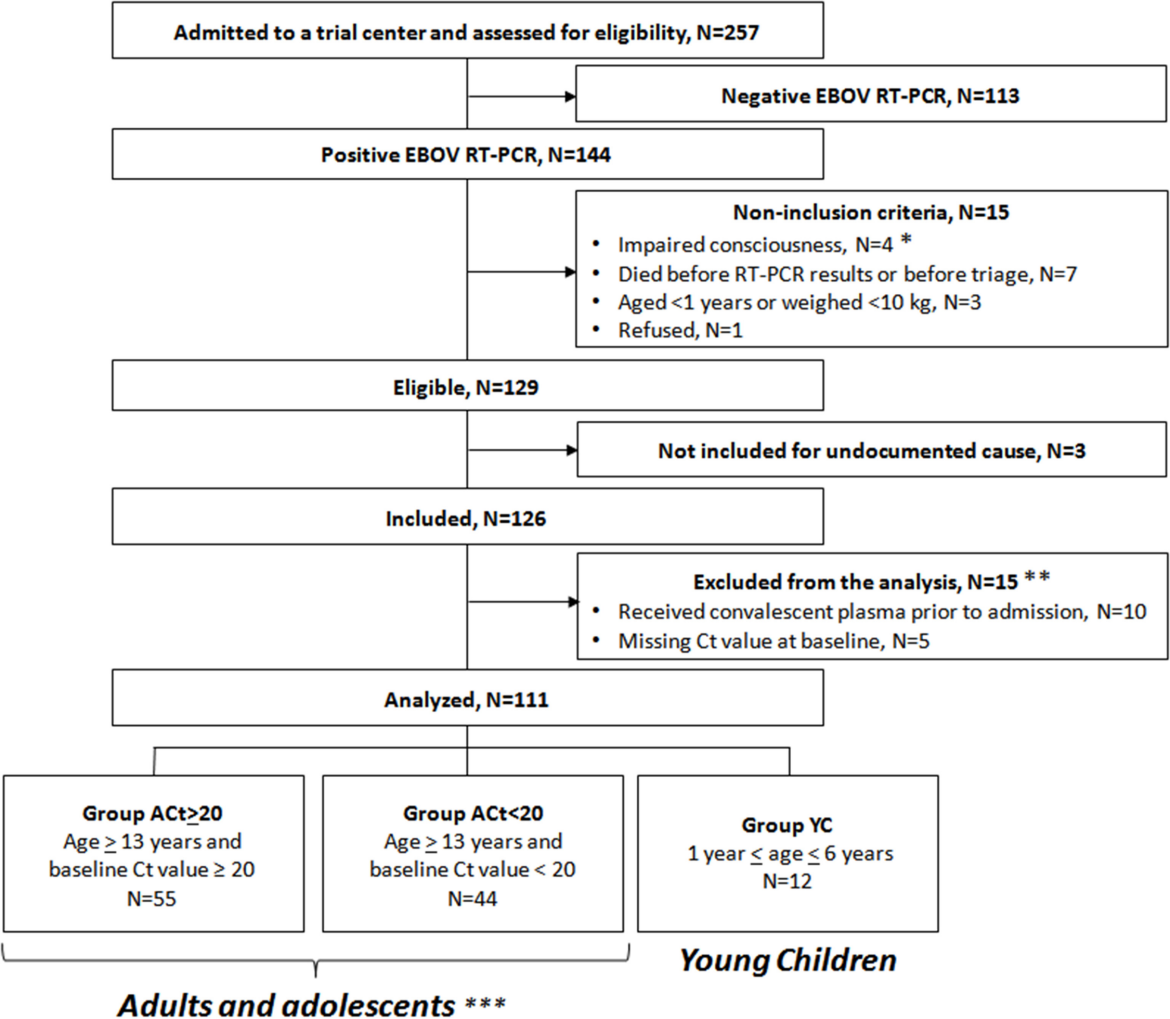
JIKI trial flow chart. *Including a pregnant woman. **Of the 15 patients excluded from the analysis, four died (received plasma: *n* = 3/10; missing Ct value, *n* = 1/5) and 11 survived. ***All aged ≥13 y because no child aged 7–12 y attended the trial centers during the study period.

During the study period, 129 individuals met the eligibility criteria, of whom 126 were included in the trial (Fig 3). Fifteen were subsequently excluded from the final analysis: ten because they had received convalescent plasma in another treatment center prior to enrollment in the trial, and five because they had no available Ct value at baseline and could not be classified according to the revised stratification. The remaining 111 patients were included in the analysis. Fifty-five belonged to Group A Ct ≥ 20, 44 to Group A Ct < 20, and 12 to Group YC. Their characteristics are presented in Table 1. As no child aged 7–12 y attended the trial centers during the study period, all patients in Group A Ct ≥ 20 and in Group A Ct < 20 were adults or adolescents aged ≥13 y. Therefore, throughout the following sections, Groups A Ct ≥ 20 and A Ct < 20 will be referred to as “adolescents and adults,” and Group YC as “young children.”

**Table 1:** JIKI trial: participants’ characteristics, according to age and baseline Ct value.

Characteristic	Young Children: Group YC (*n* = 12)	Adults and Adolescents		
		Group A Ct ≥ 20 (*n* = 55)	Group A Ct < 20 (*n* = 44)	*p*-Value^†^
**Female sex**	6 (50%)	38 (69%)	25 (57%)	0.22
**Age (years)** *	4.0 (3.5 to 5.0)	35.0 (20.0 to 50.0)	36.5 (26.5 to 52.5)	0.33
≤6 y	12 (100%)	—	—	1.00
13 to 29 y	—	22 (40%)	17 (39%)	
≥30 y, *n*	—	33 (60%)	27 (61%)	
**Time from first symptoms to admission (days)**	1.5 (1.0 to 2.5)	3.0 (2.0 to 6.0)	4.0 (2.0 to 7.0)	0.09
**Symptoms prior to admission**				
Fever	11 (92%)	49 (89%)	40 (91%)	1.00
Diarrhea	2 (17%)	24 (44%)	24 (55%)	0.32
Nausea/vomiting	3 (25%)	27 (49%)	20 (45%)	0.84
Hemorrhage	0 (0%)	2 (4%)	2 (5%)	1.00
Hiccup	0 (0%)	10 (18%)	7 (16%)	0.80
Extreme fatigue	11 (92%)	48 (87%)	43 (98%)	0.07
**Positive malaria rapid test** ^‡^	8 (67%)	6 (11%)	5 (12%)	1.00
**EBOV RT-PCR Ct value** ^§^	19.9 (16.8 to 21.4)	23.4 (21.9 to 26.6)	17.5 (16.1 to 18.4)	<0.001
<20	7 (63%)	—	44 (100%)	-
20 to 24.9	3 (27%)	33 (60%)	—	
≥25	1 (9%)	22 (40%)	—	
**EBOV viral load (log**_**10**_ **copies/ml)** **	8.8 (6.7 to 9.2)	6.8 (5.8 to 7.5)	8.7 (8.1 to 9.2)	<0.001
**Serum biochemistry**				
Creatinine (μmol/l)^ǁ^	50 (40 to 93)	108 (80 to 202)	285 (146 to 521)	<0.001
Creatinine < 110 μmol/l	6 (75%)	26 (52%)	4 (10%)	<0.001
Creatinine 110 to 299 μmol/l	2 (25%)	17 (34%)	19 (46%)	
Creatinine ≥ 300 μmol/l	0 (0%)	7 (14%)	18 (44%)	
BUN (mmol/l)	5.8 (5.2 to 12.1)	7.0 (4.3 to 14.2)	14.8 (9.0 to 25.8)	<0.001
BUN: creatinine ratio***	0.14 (0.10 to 0.21)	0.06 (0.05 to 0.08)	0.05 (0.04 to 0.07)	0.31
Sodium (mmol/l)^††^	131 (129 to 133)	132 (128 to 136)	132 (129 to 135)	0.88
Potassium (mmol/l)^‡‡^	4.8 (4.5 to 6.3)	3.8 (3.4 to 4.2)	3.9 (3.6 to 4.7)	0.34
Glucose (mmol/l)^§§^	6.7 (5.2 to 7.9)	5.0 (4.5 to 7.0)	6.0 (4.8 to 6.8)	0.98
AST (IU/l)^ǁǁ^	204 (69 to 731)	351 (118 to 1,002)	1,515 (318 to 2,000)	0.009
ALT (IU/l)^ǁǁ^	85 (21 to 313)	118 (43 to 291)	390 (267 to 581)	<0.001
ALT/AST ratio^ǁǁ^	0.23 (0.13 to 0.49)	0.32 (0.25 to 0.40)	0.21 (0.14 to 0.38)	0.02
CK (IU/l)^ǁǁ^	279 (190 to 342)	909 (490 to 2,882)	2,506 (1,002 to 4,487)	0.02
Total bilirubin (μmol/l)^ǁǁ^	103 (103 to 120)	188 (154 to 205)	205 (154 to 291)	0.09
Amylase (IU/l)^ǁǁ^	35 (24 to 37)	96 (70 to 144)	135 (85 to 195)	0.13
CRP (mg/l)^ǁǁ^	59 (36 to 64)	16 (5 to 37)	29 (15 to 49)	0.12
Albumin (g/l)^ǁǁ^	31 (29 to 34)	30 (27 to 35)	30 (29 to 36)	0.84
**Time from first symptoms to favipiravir initiation (days)**	3.0 (3.0 to 4.0)	5.0 (3.0 to 7.0)	5.0 (3.5 to 8.0)	0.24
**IV fluid rehydration during treatment**	9 (75%)	47 (85%)	44 (100%)	0.01

Data are *n* (percent) or median (interquartile range).

^†^ *p*-Values for comparison between Group A Ct ≥ 20 and Group A Ct < 20 (Fisher exact test or Wilcoxon rank sum test, as appropriate).

*As no child aged 7–12 y attended the trial centers during the study period, all patients in Group A Ct ≥ 20 and Group A Ct < 20 were adults or adolescents aged ≥13 y.

^‡^Two missing values (one in Group A Ct ≥ 20, one in Group A Ct < 20).

^§^One missing value in Group YC.

**21 missing values (six in Group YC, nine in Group A Ct ≥ 20, six in Group A Ct < 20).

^ǁ^12 missing values (four in Group YC, five in Group A Ct ≥ 20, three in Group A Ct < 20).

^¶^11 missing values (three in Group YC, five in Group A Ct ≥ 20, three in Group A Ct < 20).

***12 missing values (four in Group YC, five in Group A Ct ≥ 20, three in Group A Ct < 20).

^††^16 missing values (three in Group YC, six in Group A Ct ≥ 20, seven in Group A Ct < 20).

^‡‡^18 missing values (three in Group YC, six in Group A Ct ≥ 20, nine in Group A Ct < 20).

^§§^19 missing values (three in Group YC, nine in Group A Ct ≥ 20, seven in Group A Ct < 20).

^ǁǁ^Serum AST, ALT, CK, bilirubin, amylase, CRP, and albumin measurements were performed in three of the four trial centers (Gueckedou, Macenta, and Conakry) (*n* = 48 for AST: three in Group YC, 31 in Group A Ct ≥ 20, 14 in Group A Ct < 20; *n* = 57 for ALT: four in Group YC, 31 in Group A Ct ≥ 20, 22 in Group A Ct < 20; *n* = 57 for CK: five in Group YC, 31 in Group A Ct ≥ 20, 21 in Group A Ct < 20; *n* = 56 for bilirubin: five in Group YC, 31 in Group A Ct ≥ 20, 20 in Group A Ct < 20; *n* = 58 for amylase: five in Group YC, 31 in Group A Ct ≥ 20, 22 in Group A Ct < 20; *n* = 56 for CRP: five in Group YC, 30 in Group A Ct ≥ 20, 21 in Group A Ct < 20; *n* = 58 for albumin: five in Group YC, 31 in Group A Ct ≥ 20, 22 in Group A Ct < 20).

BUN, blood urea nitrogen; IU, international units.

### Treatment

All patients took favipiravir as per the protocol. The median time between first symptoms and first favipiravir intake was 4 d (interquartile range [IQR] 3;7). During follow-up, the 111 participants had 1,442 favipiravir intakes, including 71% with intact tablets and 29% with crushed tablets dispersed in water or fruit juice. Vomiting within 30 min of pill intake was reported in 30 instances (2%) in 21 patients, of whom 14 had one sole vomiting episode, six had two episodes, and one had four episodes.

### Mortality

Sixty participants died. The median time between favipiravir initiation and death was 3 d (IQR 2;5). All but one death occurred before day 9. The remaining death (Group A Ct ≥ 20) occurred at day 17. Our initial goal was to consider EVD mortality during the acute phase of the disease, and our hypothesis at the time the protocol was written was that all deaths would occur within 14 d. When we accessed the database containing the historical data, we found that 98% of pre-trial deaths occurred within day 14 (individuals with characteristics corresponding to Group A Ct ≥ 20, 95%; Group A Ct < 20, 100%; Group YC, 98%), and that the remaining 2% occurred between day 14 and day 21. We did not find any obvious reason to consider death before and after 14 d separately, as the goal of any treatment for EVD during the acute phase is to decrease the overall acute mortality. Therefore, even though the predefined trial endpoint was “mortality within 14 d,” throughout the following sections, we will consider “on-trial mortality,” which includes all 60 deaths. In the 51 patients who survived, the median time between favipiravir initiation and end of symptoms was 9 d (IQR 6; 12). One patient was lost to follow-up after discharge, and 50 attended the 30-d end-of-study visit. On day 30, all 50 patients were apyretic.

**Fig 4:**
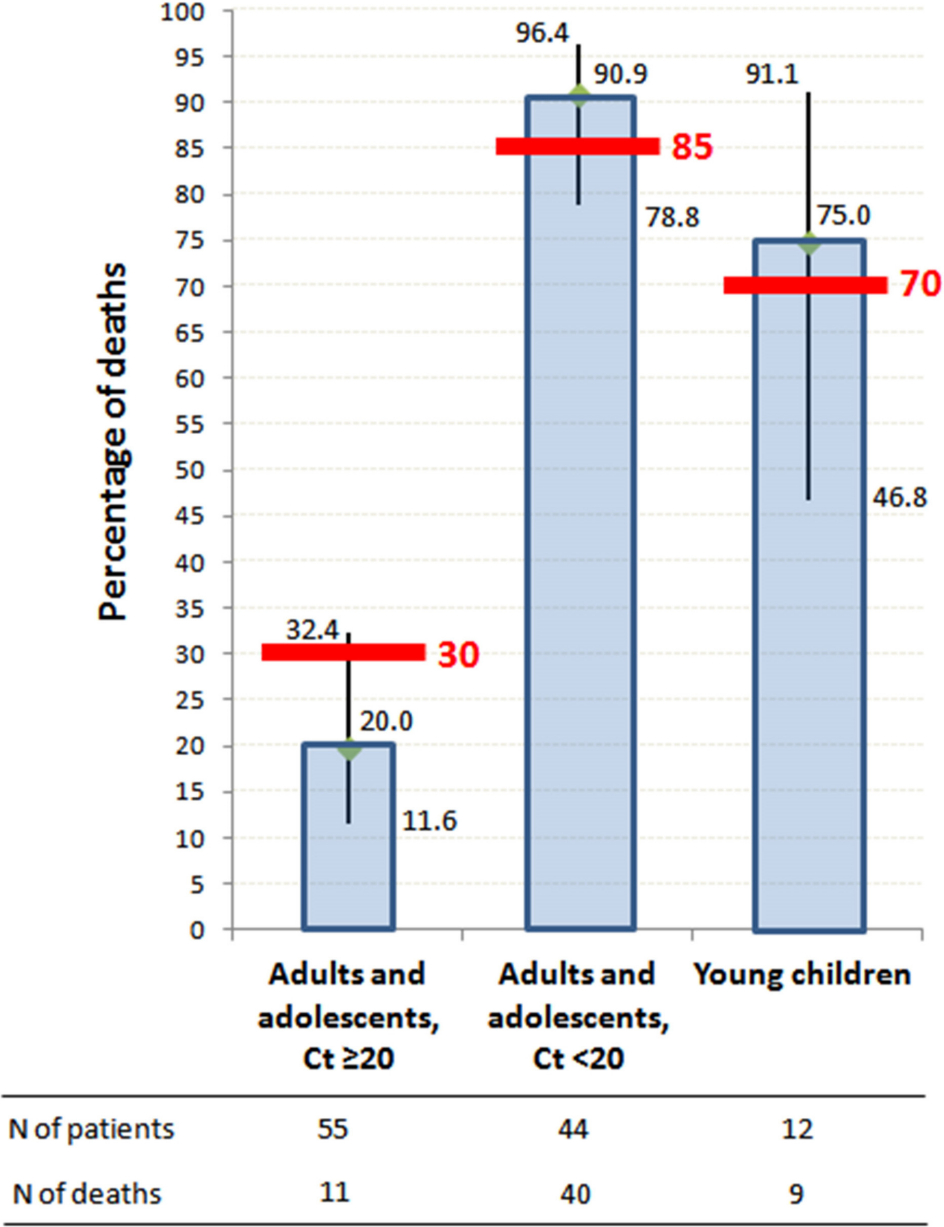
JIKI trial mortality, according to age and baseline RT-PCR Ct value. Histograms represent mortality percentages. Vertical bars represent 95% confidence intervals. Red bars represent target values. The RT-PCR assay was conducted using the RealStar Filovirus Screen RT-PCR Kit 1.0 (Altona Diagnostics).

As shown in Fig 4, on-trial mortality was 20.0% (95% CI 11.6%–32.4%) in Group A Ct ≥ 20, which is 33% lower than the target value (30%); 90.9% (95% CI 78.8%–96.4%) in Group A Ct *<* 20, which is 7% higher than the target value (85%); and 75.0% (95% CI 46.8%–91.1%) in Group YC, which is 7% higher than the target value (70%). The 95% confidence interval of the observed mortality rate included the target value for all groups. In Group A Ct ≥ 20, all deaths occurred in patients with Ct < 25, and no patient with baseline Ct ≥ 25 died.

### Secondary Endpoints in Adolescents and Adults

#### Virology

**Fig 5:**
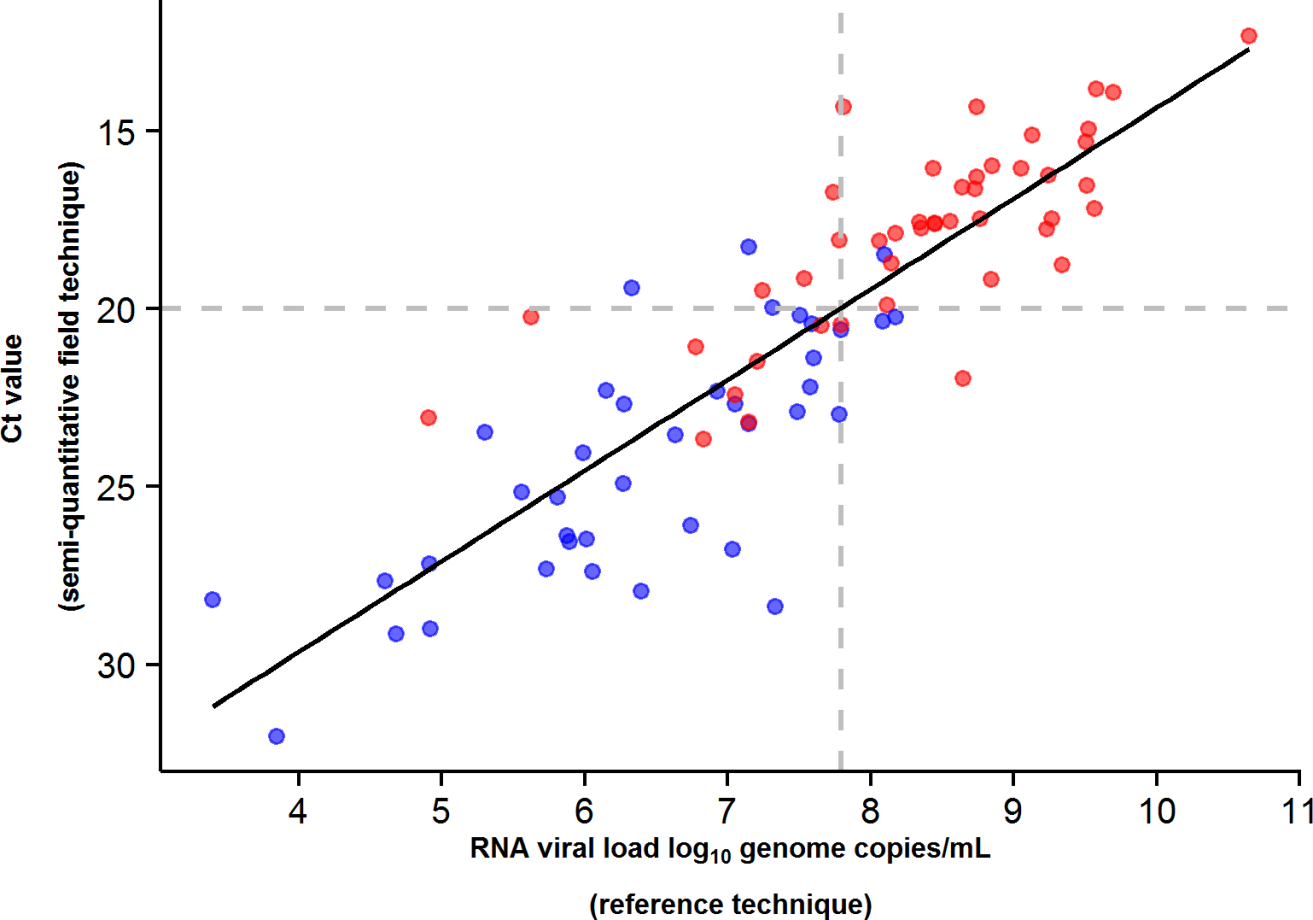
JIKI trial: correlation between EBOV RT-PCR Ct value and RNA viral load at baseline in adolescents and adults. The black line represents the regression line. The Spearman rank correlation coefficient was 0.88 (*p* < 0.001). A baseline Ct value of 20 corresponded to an RNA viral load of 5 × 10^7^ genome copies/ml of plasma (7.7 log_10_ copies/ml). Of the 99 adolescents and adults, 84 had both an RT-PCR Ct and an RNA viral load measurement at baseline. Red dots represent patients who died. Blue dots represent patients who survived.

**Fig 6:**
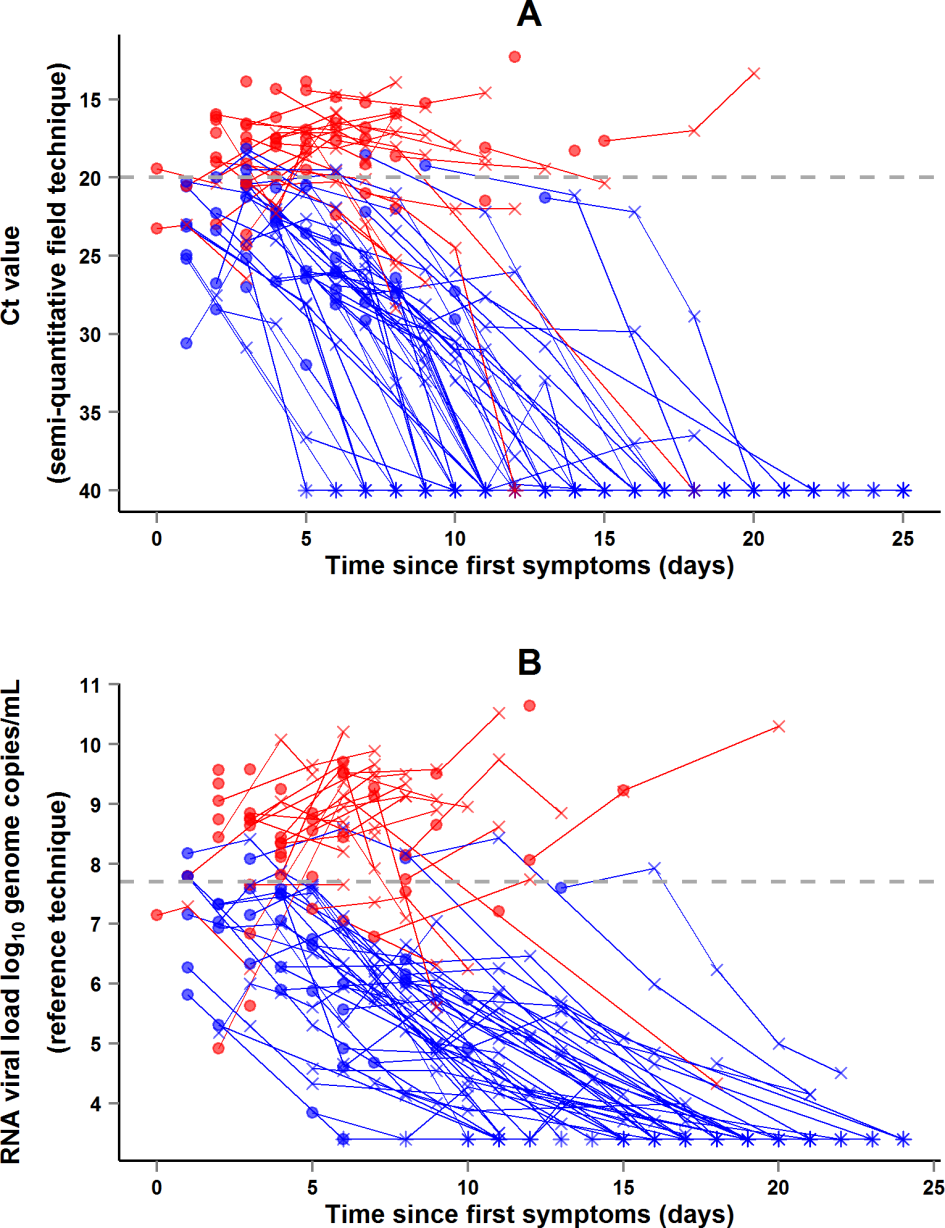
JIKI trial: evolution of RT-PCR Ct values and RNA viral load in adolescents and adults. (A) Evolution of RT-PCR Ct values. (B) Evolution of RNA viral loads. The *x*-axis represents the time since first symptoms (for example, for a patient whose first symptom occurred 5 d before admission to the treatment center, the baseline value dot is positioned at 5 d). Each line represents one patient. Dots represent baseline values, X’s represent follow-up values, and asterisks represent values below the limit of quantification. Red symbols represent patients who died; blue symbols represent patients who survived.

**Fig 7:**
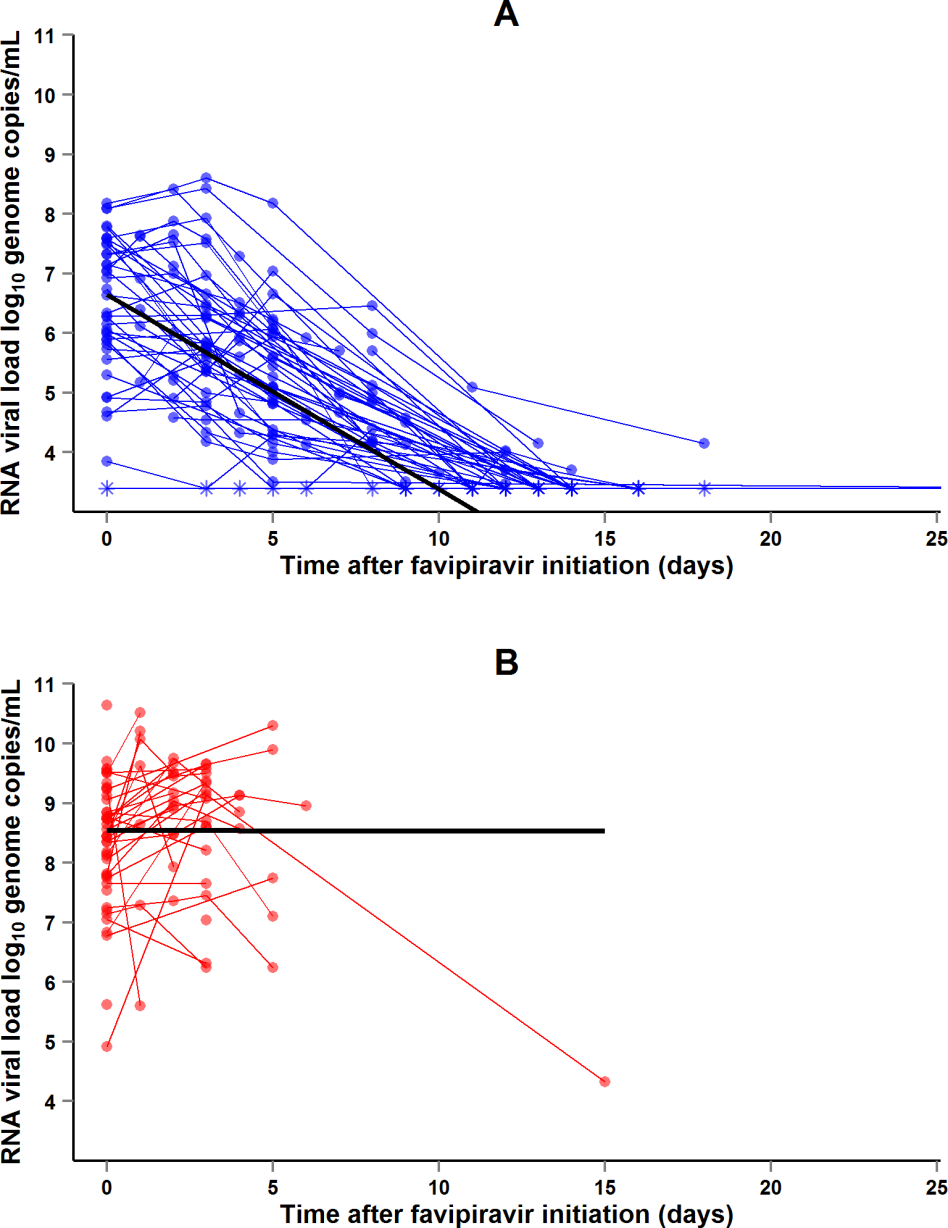
JIKI trial: evolution of RNA viral load in adolescents and adults. (A) Evolution of RNA viral load in adolescents and adults who survived. The *x*-axis represents the time since favipiravir initiation (day 0). Each blue line represents one patient (*n* = 48). Circles represent baseline and follow-up values. Asterisks represent values under the limit of detection (3.4 log_10_ copies/ml). The black line represents the mean log_10_ viral load decline estimated from a linear mixed effect model. The mean initial viral load was estimated at 6.65 log_10_ copies/ml (SD 0.86), and the mean slope of viral load evolution at −0.33 log_10_ copies/ml (SD 0.03) per day of follow-up. (B) Evolution of RNA viral load in adolescents and adults who died. The *x*-axis represents the time since favipiravir initiation (day 0). Each red line represents one patient (*n* = 49). Circles represent baseline and follow-up values. The black line represents the mean log_10_ viral load evolution estimated from a linear mixed effect model. The mean initial viral load was estimated at 8.54 log_10_ copies/ml (SD 0.21), and the mean slope of viral load evolution at −0.001 log_10_ copies/ml (SD 0.15) per day of follow-up.

During the entire follow-up period, there were 371 RT-PCR Ct measurements and 295 RNA viral load measurements. Baseline RT-PCR Ct and RNA viral load were well correlated (correlation coefficient 0.88, *p* < 0.001). A baseline Ct value of 20 corresponded to a RNA viral load of 5 × 10^7^ genome copies/ml of plasma (7.7 log_10_ copies/ml) (Fig 5). Mortality was much lower in patients with baseline Ct ≥ 20 compared to those with baseline Ct < 20 (20% versus 91%, *p* < 0.001), and in patients with baseline viral load ≤ 7.7 log_10_ copies/ml compared to those with viral load > 7.7 log_10_ copies/ml (23% versus 88%, *p* < 0.001). Fig 6 shows the dynamics of Ct values (Fig 6A) and RNA viral loads (Fig 6B) in patients who survived and in those who died. In all but two patients who died, there was no significant decrease in viral load before death. In patients who survived, the mean decrease in viral load was estimated at 0.33 (SD 0.03) log_10_ copies/ml per day of follow-up (Fig 7). All patients who survived had two negative RT-PCR tests before discharge.

#### Renal function

**Fig 8:**
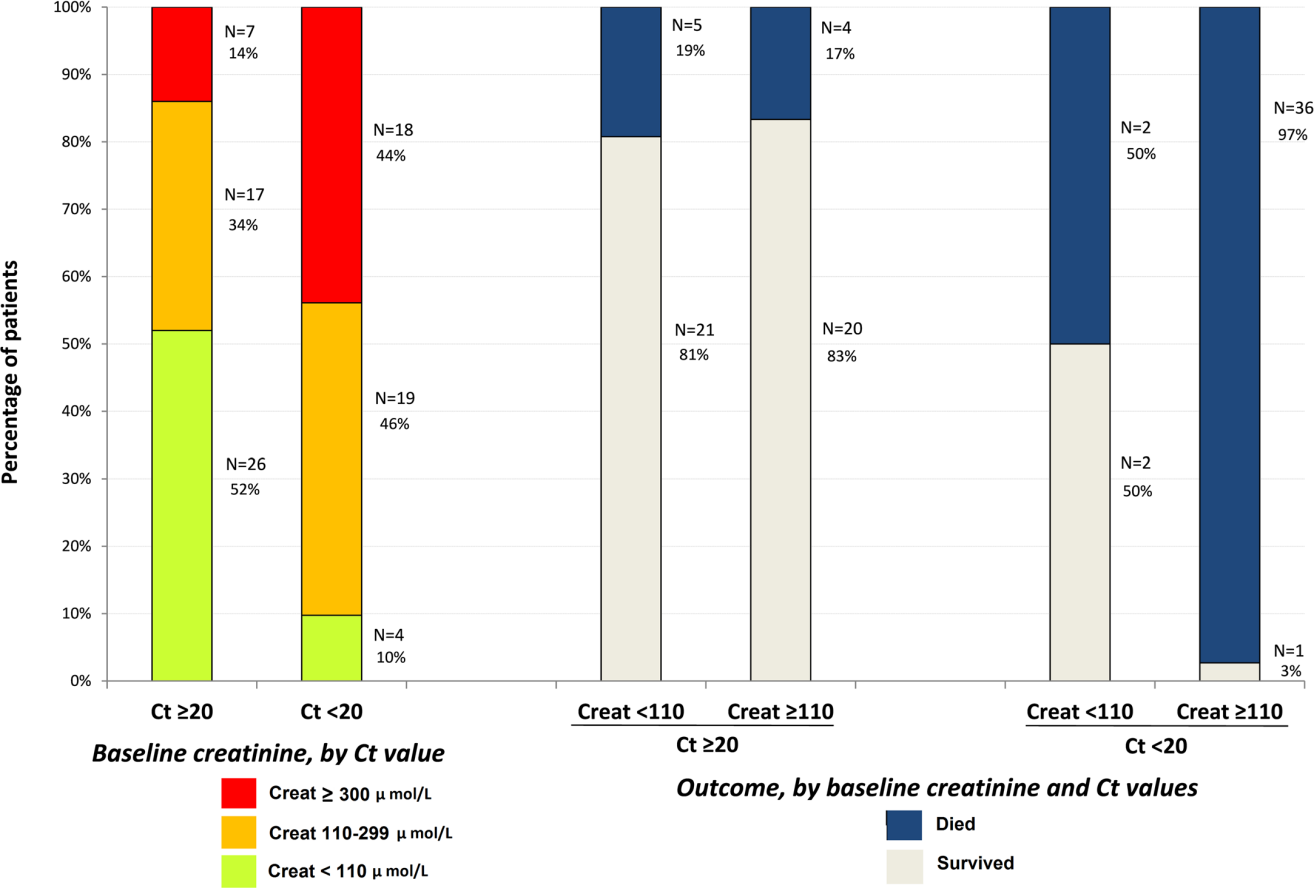
JIKI trial: baseline serum creatinine in adolescents and adults and outcomes according to baseline values. Creat, creatinine.

**Fig 9:**
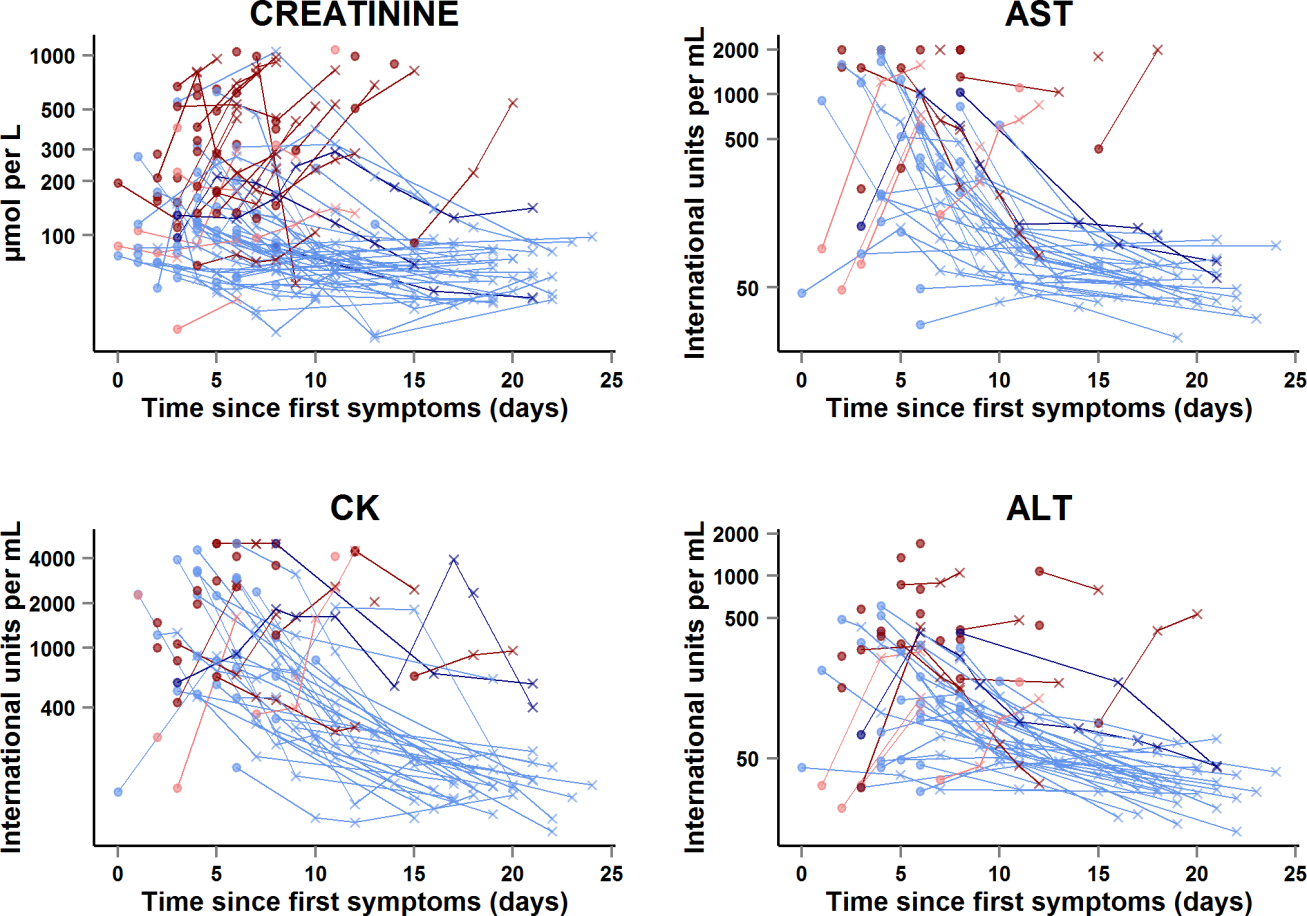
JIKI trial: evolution of serum creatinine, aspartate aminotransferase, alanine aminotransferase, and creatine phosphokinase in adolescents and adults. The *x*-axis represents the time since first symptoms (for example, for a patient whose first symptom occurred 5 d before day 0, the baseline value dot is positioned at 5 d). Each line represents one patient. Dots represent baseline values, and X’s represent follow-up values. Red symbols represent patients who died; blue symbols represent patients who survived. Dark red lines represent patients with baseline Ct < 20 who died, light red lines represent patients with baseline Ct ≥ 20 who died, dark blue lines represent patients with baseline Ct < 20 who survived, and light blue lines represent patients with baseline Ct ≥ 20 who survived. Samples obtained more than 25 d after onset of symptoms are not represented.

During the entire follow-up period, there were 289 serum creatinine measurements. Renal function impairment was much more frequent, advanced, and related to death in Group A Ct < 20 than in Group A Ct ≥ 20. In Group A Ct < 20, baseline serum creatinine was ≥110 μmol/l in 90% of patients, including 44% with creatinine ≥ 300 μmol/l, and 97% of patients whose baseline creatinine was ≥110 μmol/l died. In Group A Ct ≥ 20, baseline creatinine was ≥110 μmol/l in 48% of patients, including 14% with creatinine ≥ 300 μmol/l, and 17% of patients with a baseline creatinine ≥ 110 μmol/l died (Fig 8). Creatinine levels returned to normal values before day 30 in all patients who survived (Fig 9).

#### Transaminases and creatine phosphokinase

In the subset of 58 adults and adolescents who were hospitalized at the Gueckedou, Macenta, or Conakry trial centers, there were 151 AST measurements, 164 ALT measurements, and 160 CK measurements during the entire follow-up period (Fig 9). Abnormal values at baseline were more frequent, advanced, and related to death in Group A Ct < 20 than in Group A Ct ≥ 20. In Group A Ct < 20, AST was ≥1,000 IU/l in 12/18 (67%) patients, of whom 11 (92%) died, and CK was ≥4,000 IU/l in 7/23 (30%) patients, of whom six (86%) died. In Group A Ct ≥ 20, AST was ≥1,000 IU/l in 8/31 (26%) patients, of whom one (12%) died, and CK was ≥4,000 IU/l in 4/32 (12%) patients, of whom two (50%) died. The median ALT/AST ratio was 0.29 (IQR 0.21; 0.40), with no significant difference between Group A Ct < 20 and Group A Ct ≥ 20.

#### Drug tolerance

No grade 3 or 4 clinical event was considered to be related to the drug by the investigators. All deaths were associated with uncontrolled EBOV viremia and disease progression.

Many patients had grade 3 or 4 impaired biochemical values at baseline, that is, before they received favipiravir. Therefore, we detail here all available biochemical parameters at baseline and during follow-up, in order to show the evolution in all patients, including in those with very high values at baseline.

**Fig 10:**
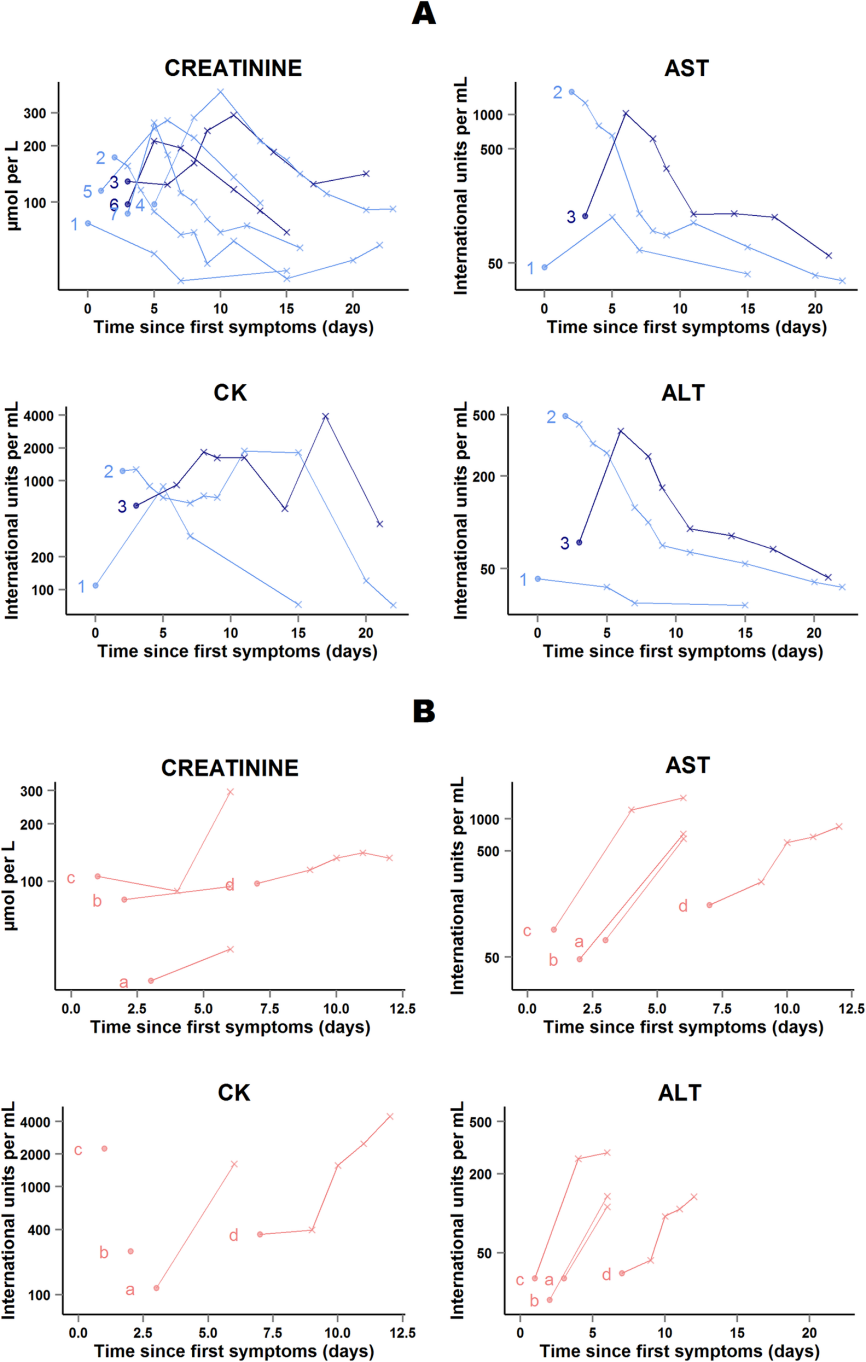
JIKI trial: evolution of serum creatinine, transaminases, and creatine phosphokinase in the 11 adolescents and adults who had worsening in at least one biochemical parameter on favipiravir. (A) Patients who survived (*n* = 7). (B) Patients who died (*n* = 4). Each line represents one patient. All patients are identified with an ID number (from 1 to 7) or letter (from a to d) throughout the figures. For all 11 patients, all available data are shown. Dots represent baseline values, and X’s represent follow-up values. Dark blue lines represent patients with baseline Ct < 20 who survived. Light blue lines represent patients with baseline Ct ≥ 20 who survived. Light red lines represent patients with baseline Ct ≥ 20 who died. The *x*-axis represents the time since first symptoms (for example, for a patient whose first symptom occurred 5 d before admission to the treatment center, the baseline value dot is positioned at 5 d). Samples obtained more than 25 d after onset of symptoms are not represented. All 11 patients continued favipiravir.

In 41 of the 48 patients who survived, abnormal serum levels of creatinine and, when available, transaminases and CK rapidly improved on treatment. The remaining seven patients presented transient on-treatment increases in one or another of these markers. All patients continued favipiravir, and levels normalized before discharge (Fig 10A).

In four of the 11 patients who died in group A Ct ≥ 20, serum creatinine and, when available, transaminases increased before death (Fig 10B). The cause of death was considered to be EVD in each case. Their last available EBOV plasma RNA values were 9.4, 9.1, 9.0, and 7.7 log_10_ copies/ml; their last available ALT measures were 112, 135, 290, and 134 IU/l; and their last available AST measures were 650, 724, 1,579, and 848 IU/l. Only two of these four patients presented on-treatment increase in creatinine before death. The last available creatinine values for these two patients were 296 and 132 μmol/l.

### Early Treatment

Of the 99 adults and adolescents for whom data were analyzed, 31 started favipiravir within <72 h of first symptoms and 68 started favipiravir ≥72 h after first symptoms. There was no significant difference between adults who started favipiravir within <72 h and those who started favipiravir ≥72 h after first symptoms in terms of baseline Ct (median 20.5 [IQR 18.7; 23.4] versus median 20.2 [IQR 17.5; 24.7], *p* = 0.6), baseline RNA viral load (median 7.6 log_10_ copies/ml [IQR 7.0; 8.6] versus median 7.6 log_10_ copies/ml [IQR 6.1; 8.6], *p* = 0.7), and mortality (45.2% [95% CI 27.3–64.0] versus 54.4% [95% CI 41.9–66.5], *p* = 0.5). All other baseline and follow-up characteristics were similar between patients who started favipiravir <72 h versus ≥72 h after first symptoms, with the exception of baseline creatinine (median 115 μmol/l [IQR 78; 208] versus median 170 μmol/l [IQR 109; 398], *p* = 0.01).

### Baseline and Follow-Up Characteristics of Patients Who Survived and Those Who Died

Baseline and follow-up characteristics of patients who survived and those who died are shown in S4 Table. Compared to patients who survived, those who died were older, had higher median RNA viral load, lower median Ct value, and higher serum creatinine and blood urea nitrogen levels at baseline, and more severe diarrhea, more severe pain, more frequently impaired consciousness, and more frequent hemorrhage during their hospital stay.

### Secondary Endpoints in Young Children

Of the 12 children ≤6 y of age (Group YC), eight had a positive malaria rapid test at baseline; seven had a baseline Ct < 20, four had a baseline Ct ≥ 20, and one had no available Ct value at baseline. Baseline serum AST was >1,000 IU/l in one child. The median ALT/AST ratio was 0.23. No child presented a baseline CK value > 4,000 IU/l. Baseline serum creatinine value was available for eight children, of whom two had a creatinine value between 110 and 300 μmol/l. In the three children who survived, the baseline Ct values were 19.9, 28.4, and unavailable, and the baseline viral RNA load was 9.10 log_10_ copies per ml and unavailable (two children). Baseline and follow-up Ct, RNA viral load, creatinine, transaminase, and CK values in children ≤6 y are detailed in S3 Fig

### Other Secondary Endpoints

The protocol mentioned three additional secondary endpoints: evolution of infectious loads, viral micro-diversity, and plasma trough concentrations of favipiravir. Because the amount of plasma we were able to collect in the biobank was not sufficient to measure these outcomes in all patients after viral loads were measured, and because of technical challenges that will require time to deal with, all three outcomes will be measured secondarily in selected samples. Plasma trough concentrations will be included in a model using data from various studies, including this trial and animal studies, to update our simulation of drug doses and draw updated conclusions on the most appropriate dose of favipiravir to reach the target plasma concentration.

## Discussion

This trial was put together in 13 wk, in remote treatment centers, in an environment of community tensions, and in a country that had little experience in clinical trials. A first lesson to be drawn from this experience is that, although difficult, emergency trials are feasible in the context of a deadly contagious disease outbreak. This trial was achieved as a result of good collaboration between investigators, health authorities, NGOs, community leaders, and patients.

The JIKI trial is, to our knowledge, the first to evaluate a specific anti-EVD treatment and the largest that has been performed in EVD to date. This trial presented many challenges from inception to the final analysis, which have been met pragmatically. The results of the study, as well as the challenges that were encountered, provide useful information for future clinical research in EVD.

### Is Favipiravir Effective in the Treatment of EVD?

From the outset, we were aware that the JIKI trial would not be able to answer this question, but rather to identify trends and to orientate the design of future studies that may provide a definitive answer. The results of the study indicate that monotherapy with favipiravir is unlikely to be effective in patients with very high viremia and merits further investigation in patients with intermediate to high viremia. This conclusion is based on two findings, namely, the observed mortality rates and the dynamics of EBOV RNA load on treatment. In Group A Ct < 20, mortality was 7% higher than the target value and viral loads did not decrease. This suggests that any future trial is unlikely to demonstrate any benefit of favipiravir in these patients. In Group A Ct ≥ 20, mortality was 33% lower than the target value and viremia decreased rapidly on treatment. The trial was non-randomized, the statistical power was low, and the 95% CI of mortality included the target value. Therefore, this finding does not prove that favipiravir was effective in these patients but only suggests that the question remains open and gives some indication on how to better address it.

A third set of findings related to biological markers of organ damage also supports the pertinence of stratification of patients according to viral load. Patients with very high viremia had levels of creatinine, transaminases, and CK that suggested established organ failures requiring intensive care, while patients with a medium to high viral load had less frequent and, when present, reversible organ failures. In the context of limited resource availability, the former type of patients is unlikely to benefit from any specific monotherapy, while the latter is more likely to benefit from specific interventions.

The lesson that we draw from this is that future drug trials in EVD should systematically stratify analyses by baseline level of viremia, and that future research on favipiravir should focus on patients with a Ct value ≥ 20. The utility of such a stratification became apparent in January 2015 from an interim analysis of the first 80 patients included in the study [36], and the protocol and statistical analysis plan of the JIKI trial were amended accordingly.

### Would It Have Been Better to Perform a Randomized Study at the Outset in Order to Demonstrate Efficacy Unequivocally?

Some authors have criticized non-randomized studies and have recommended performing randomized controlled trials (RCTs) at the outset [39,40]. This is a central debate. There is no doubt that randomized designs should be used whenever possible, as they are the best way to unequivocally demonstrate the efficacy and safety of an experimental drug. During this recent outbreak, however, we were faced with two arguments that made us decide not to use a randomized design at the outset. The first argument was the perception by both Ebola care workers and researchers in the field that it would be unethical to allocate patients from within the same family or village to receive or not receive an experimental drug, using a randomization process that would be unlikely to be understood by very sick and/or distrustful patients. The second argument was the need to build a partnership with a community that had no experience in emergency research. In the context of Ebola—with countrywide disruption of normal routines, isolation measures perceived as coercive [28], and rumors about and distrust of Ebola treatment centers—a randomized trial was deemed likely to worsen the situation and lead even more patients to refuse to seek care [29].

Both arguments were strong, in the context of an outbreak at its peak, with care centers being crowded and having no previous experience in Ebola research. As time passed by, the outbreak weakened, the rate of patients showing up at Ebola treatment centers fell below one per day, the centers gained experience with research, and the possibility that a randomized trial would become eventually ethically acceptable increased. Timeliness, experience, and community perception are key elements to the debate surrounding the design of trials in emergency situations [29].

During the recent outbreak, three non-randomized trials were launched at the outset [36,41,42], and one RCT was initiated later, in March 2015. The RCT was a trial of ZMapp, a combination of monoclonal antibodies [43]. The amount of preclinical data available for this treatment, notably in non-human primates, was larger than for favipiravir [44]. The March 2015 version of the ZMapp RCT protocol specified a stratification of inclusion and data analysis by Ct value [43], on the basis of the preliminary data of the JIKI trial presented in February 2015 at the 22nd Conference on Retroviruses and Opportunistic Infections [36]. Because the ZMapp trial started within the late phase of the outbreak, the rate of patients showing up for care was low. Its results, when published, will tell us whether it had been possible to recruit sufficient patients in this RCT in order to conclude on the efficacy of the treatment. Even if this was not the case, however, the randomized design will add strength to the results, making them more likely to allow valid inferences to be made regarding drug efficacy and tolerance and to inform the design of future studies.

Cooper et al. recently proposed a multi-stage approach including both non-randomized and randomized elements, triaging treatments into those with no effect that should be discarded quickly, those with clear benefits that should be used immediately, and those with promise, requiring evaluation in an RCT. This triage approach uses initial single-arm studies where all patients receive the treatment, which is what we did in the JIKI trial [45].

The findings of the JIKI trial provide data on potential treatment effect size that can be used to design a future randomized trial of favipiravir based on realistic a priori hypotheses. In such a trial, the target population should consist of patients with a Ct value ≥ 20 (or an equivalent threshold using a different PCR technique), the primary outcome variable should be mortality, the anticipated mortality rate in the control group should be 30%, and the trial should have sufficient statistical power to demonstrate a reduction in mortality in the favipiravir group of 30%. To this end, the trial would need to include at least 784 patients overall (392 by arm). This simple sample size estimation suggests that it will be difficult to recruit enough patients with EVD for a trial assessing any treatment with interesting potential clinical efficacy that is nevertheless around 30% in a group of patients in which the reference mortality rate is expected to be around 30%. The scientific community still needs to consider the best way to determine unequivocal proofs of efficacy in such conditions [45,46].

### Is High-Dose Favipiravir Well Tolerated?

Conclusions regarding safety can only be drawn from observations in Group A Ct ≥ 20. In Group A Ct < 20, most of the patients died rapidly, and, even though these deaths were all attributed to EVD, it is not possible with the available paraclinical data to affirm unequivocally the absence of toxicity of favipiravir.

In contrast, the findings in Group A Ct ≥ 20 provide a convincing suggestion of the good tolerability of favipiravir. In this group, the patients who died all had a high viral load together with clinical and biochemical abnormalities that were clearly consistent with uncontrolled EVD. All the other patients in this group, even those with very abnormal biochemical markers at baseline or who developed highly abnormal values during follow-up, survived. None of these patients needed to discontinue favipiravir treatment, and all achieved normal values for biochemical markers on treatment. These findings suggest that favipiravir can be used at a daily dose twice higher and for a duration twice longer than that used for the treatment of influenza. If necessary, it would be acceptable to test higher doses.

### What Did We Learn about the Physiopathology of EVD?

Proof-of-concept trials can rapidly provide new findings that can be shared with the scientific community and help increase knowledge to facilitate clinical research in the emergency setting. When the JIKI trial was initiated, little information was available on the relationship between EBOV and renal function [7,24,25]. Similarly, although a few studies had reported data on viral load in EVD [22,47,48], no trial had used Ct as a clinical marker in the field. In February 2015, after we found interesting results in an interim analysis of the JIKI trial, we reported the frequency of renal failure, the relationship between renal failure and low Ct values, and the powerful prognostic value of low Ct values [36]. Since our communication, these results have been confirmed by others, and creatinine and Ct have become the two most important markers used in the field for evaluating patient prognosis [49,50].

### Ct Value and RNA Viral Load

We used a single semi-quantitative RT-PCR test in the field to obtain Ct values and then re-tested all samples with a quantitative reference technique measuring the number of genome copies per milliliter in a centralized reference laboratory. As the correlation between Ct and RNA genome copy values was strong, we are confident that field Ct values were measured homogeneously. We conclude that the Ct value is acceptable as a real-time surrogate marker of real viral load. To our knowledge, this is the first study to validate such a correlation in trial conditions.

Using the pre-trial database, we found that a threshold of 20 cycles was a highly sensitive cutoff to separate two groups of patients, with patients with Ct < 20 having an adjusted odds ratio of mortality of 11.1 compared to those with Ct ≥ 20. A powerful predictor yielding such a high odds ratio is very rare in clinical practice. During the trial, the 20-cycle threshold was robust and had similar strong predictive value for mortality. However, because the Ct value may vary depending on technique and technician experience, the 20-cycle threshold that we identified may not be universally replicable. Virologists should work at the international level to standardize techniques and validate a robust field reference.

Finally, we recorded a rapid decrease in viral load in patients in Group A Ct ≥ 20. Since there were no historical reference data on the evolution of EBOV viral load in large patient cohorts, it was not possible to interpret this as related to the drug. However, to our knowledge, this is the first study after the report by Towner et al. [22] in 2004 to report the evolution of viral load in EVD systematically using repeated measures. We estimated a mean decrease of 0.35 log_10_ genome copies/ml per day of follow-up. This may be useful as a benchmark to compare the dynamics of EBOV viral load in future drug trials [51].

### Renal Function

We found a higher than expected rate of renal dysfunction and acute kidney injury. Most highly viremic patients had elevated creatinine values and died. Renal function in patients with intermediate viremia was less frequently impaired, and most of these patients survived with fluid management. These findings suggest both a reduction in circulating volume due to vomiting and diarrhea, and acute kidney injury resulting from the visceral consequences of the disease [7]. The latter may include acute tubular necrosis, microvascular thrombi, or sepsis-like kidney injury occurring in the context of a systemic inflammatory response with multi-organ failure [5,52,53]. The high frequency of elevated transaminases, mainly affecting AST and associated with normal bilirubin values and elevated CK values, was more evocative of muscular tissue injury than liver injury. Such myositis could be due to direct viral invasion of myocytes or to virus-mediated inflammatory mechanisms involving myotoxic cytokines [54–56]. A leakage of muscle cell contents, including sarcoplasmic proteins, in the circulation could also contribute to kidney injury [57–59].

### Is it Important to Treat Early?

We found no difference between patients who showed up within 3 d of first symptoms and those who did not in terms of viral load or mortality, both in the trial and in the historical database. This may suggest that using self-report of symptom onset as a proxy of early clinic attendance is unreliable, or that peak viral load and the consequences of the disease may be present within hours of first symptoms in some patients, even if we know that this is not the case in others. This tells us nothing about the efficacy of favipiravir, as our study is not conclusive on this point. If favipiravir or any other treatment is shown to be effective in the future, this lack of difference also does not argue against treating patients as early as possible, but rather suggests that it may be necessary to treat patients within hours rather than days of infection. Further research should address rapid testing techniques, accelerated test-and-treat procedures, and preventive treatment for high-risk contacts [60].

### Conclusion

Almost two years after the start of the EVD epidemic in West Africa, no effective treatment has yet be demonstrated. In future epidemics, it will be important to integrate clinical research into standard care. All patients with EBOV infections should be eligible for large, internationally coordinated trials of experimental treatments in order to identify effective treatments with the best level of evidence of efficacy possible.

In the midst of an Ebola outbreak, researchers may be faced with elements that make them feel that randomizing patients to receive either standard care or standard care plus an experimental drug is not ethically acceptable. In these rare circumstances, it can be decided to not run a trial and to wait for more favorable conditions, or to run a non-randomized trial. In this pilot experience, we did the latter. Our conclusions are nuanced. On the one hand, we cannot conclude on the efficacy of the drug, and our conclusions on tolerance, although encouraging, cannot be as firm as they would have been if we could have used randomization. On the other hand, we learned a lot about how to quickly set up and run a trial in such unusual circumstances and in close relationship with the community and NGOs, we integrated research into care so that it improved care, we rapidly generated and shared with the scientific community intermediate data that were useful for designing Ebola research, and we gathered evidence that will allow researchers to base further trials on strong preliminary assumptions.

Our study provided no evidence that favipiravir monotherapy at this dose might have a favorable benefit/risk ratio in patients with very high viral load. This does not prove that favipiravir could not provide a benefit in a person with very high viral load, but suggests that further studies in the same context will be very unlikely to show that monotherapy with favipiravir decreases mortality in patients with very high viral load. In contrast, in patients with a Ct ≥ 20 or a viral load below 10^8^ genome copies/ml, future research on favipiravir monotherapy would be merited. As the changes that occur in patients with severe sepsis can influence pharmacokinetic parameters [61,62], it may also be of interest to evaluate higher doses than those used in the JIKI trial.

## Supporting Information

S1 Fig Historical database: pre-trial EVD weekly case fatality rates from MSF Ebola treatment centers in forested Guinea during the 3 mo preceding JIKI trial start. The historical data include all patients with confirmed EVD who presented at the MSF Ebola treatment centers in forested Guinea from 15 September 2014 (week 38) to 14 December 2014 (week 50) (*n* = 540); the black line is the observed case fatality rate per week; the red dotted line is the centered moving average case fatality rate of order 3 (trend based on a 3-wk moving average).
(TIF)

S2 Fig Historical database: pre-trial mortality in patients with EVD according to baseline RT-PCR Ct values. The historical data include all patients with confirmed EVD who presented at the MSF Ebola treatment centers in forested Guinea during the 3 mo preceding JIKI trial start (15 September 2014 to 14 December 2014). (A) Pre-trial mortality rate in each stratum of baseline RT-PCR Ct value. (B) Specificity, sensitivity, and Youden’s index (sensitivity + specificity − 1) of baseline RT-PCR Ct value for mortality. The Youden index was maximum for a baseline RT-PCR Ct value of 20.2.
(TIF)

S3 Fig JIKI trial: evolution of RT-PCR Ct values, RNA viral load, creatinine, AST, ALT, and CK in young children (≤6 y of age). The *x*-axis represents the time since first symptoms (for example, for a patient whose first symptom occurred 5 d before admission to the treatment center, the baseline value dot is positioned at 5 d). Each line represents one patient. Dots represent baseline values, X’s represent follow-up values, and asterisks represent values below the limit of quantification. In all panels, red symbols represent patients who died, and blue symbols represent patients who survived. Dark red lines represent patients with baseline Ct < 20 who died, light red lines represent patients with baseline Ct ≥ 20 who died, dark blue lines represent patients with baseline Ct < 20 who survived, and light blue lines represent patients with baseline Ct ≥ 20 who survived. Lines with no initial dot represent patients with unavailable baseline values (only follow-up values are available). Samples obtained more than 25 d after onset of symptoms are not represented.
(TIF)

S1 Table JIKI trial preparation: power provided by a sample size of 60 participants to demonstrate that mortality in the trial is significantly below a target value, according to different hypotheses of target values and mortality. (PDF)

S2 Table Historical database: characteristics of patients with EVD hospitalized in MSF Ebola treatment centers in forested Guinea during the 3 mo preceding JIKI trial start (15 September 2014 to 14 December 2014). (PDF)

S3 Table Historical database: factors associated with pre-trial mortality in patients with EVD hospitalized in MSF Ebola treatment centers in forested Guinea during the 3 mo preceding JIKI trial start (15 September 2014 to 14 December 2014). (PDF)

S4 Table JIKI trial: baseline and follow-up characteristics in adult and adolescents who died and in those who survived during the trial. (PDF)

S5 Table Raw data used to generate Figs 5, 6, 7, 9 and 10. (XLSX)

S1 Text Trial protocol. (PDF)

S2 Text CONSORT statement. (PDF)

S3 Text Infomation sheets and consent forms. (PDF)

## Abbreviations

ALT: alanine aminotransferase
AST: aspartate aminotransferase
CK: creatine phosphokinase
CRP: C-reactive protein
Ct: cycle threshold
DSMB: data safety monitoring board
EBOV: Ebola virus
EVD: Ebola virus disease
IQR: interquartile range
MSF: Médecins sans Frontières
NGO: non-governmental organization
RCT: randomized controlled trial
RT-PCR: reverse transcription PCR
SD: standard deviation

## References

[1] Baize S, Pannetier D, Oestereich L, Rieger T, Koivogui L, Magassouba N, et al Emergence of Zaire Ebola virus disease in Guinea—preliminary report. N Engl J Med. 2014;371:1418–1425. 10.1056/NEJMoa1404505 24738640

[2] Kuhn J, Andersen K, Baize S, Bào Y, Bavari S, Berthet N, et al Nomenclature- and database-compatible names for the two Ebola virus variants that emerged in Guinea and the Democratic Republic of the Congo in 2014. Viruses. 2014;6:4760–4799. 10.3390/v6114760 25421896 PMC4246247

[3] World Health Organization. Ebola virus disease (EVD) in West Africa: an extraordinary epidemic. Wkly Epidemiol Rec. 2015;90:89–96. 25745678

[4] Leroy E, Baize S, Volchkov V, Fisher-Hoch S, Georges-Courbot M, Lansoud-Soukate J, et al Human asymptomatic Ebola infection and strong inflammatory response. Lancet. 2000;355:2210–2215. 10881895 10.1016/s0140-6736(00)02405-3

[5] Feldmann H, Geisbert TW. Ebola haemorrhagic fever. Lancet. 2011;377:849–862. 10.1016/S0140-6736(10)60667-8 21084112 PMC3406178

[6] West TE, von Saint André-von Arnim A. Clinical presentation and management of severe Ebola virus disease. Ann Am Thorac Soc. 2014;11:1341–1350. 10.1513/AnnalsATS.201410-481PS 25369317

[7] Schieffelin JS, Shaffer JG, Goba A, Gbakie M, Gire SK, Colubri A, et al Clinical illness and outcomes in patients with Ebola in Sierra Leone. N Engl J Med. 2014;371:2092–2100. 10.1056/NEJMoa1411680 25353969 PMC4318555

[8] Stephens DS, Ribner BS, Gartland BD, Feistritzer NR, Farley MM, Larsen CP, et al Ebola virus disease: experience and decision making for the first patients outside of Africa. PLoS Med. 2015;12:e1001857 10.1371/journal.pmed.1001857 26218574 PMC4517924

[9] Lyon GM, Mehta AK, Varkey JB, Brantly K, Plyler L, McElroy AK, et al Clinical care of two patients with Ebola virus disease in the United States. N Engl J Med. 2014;371:2402–2409. 10.1056/NEJMoa1409838 25390460

[10] Mora-Rillo M, Arsuaga M, Ramirez-Olivencia G, de la Calle F, Borobia AM, Sanchez-Seco P, et al Acute respiratory distress syndrome after convalescent plasma use: treatment of a patient with Ebola virus disease contracted in Madrid, Spain. Lancet Respir Med. 2015;3:554–562. 10.1016/S2213-2600(15)00180-0 26041403

[11] World Health Organization. Categorization and prioritization of drugs for consideration for testing or use in patients infected with Ebola. 2015 Jan 19 [cited 15 Sep 2015]. Available: http://www.who.int/medicines/ebola-treatment/cat_prioritization_drugs_testing/en/.

[12] Smither SJ, Eastaugh LS, Steward JA, Nelson M, Lenk RP, Lever MS. Post-exposure efficacy of oral T-705 (favipiravir) against inhalational Ebola virus infection in a mouse model. Antiviral Res. 2014;104:153–155. 10.1016/j.antiviral.2014.01.012 24462697

[13] Furuta Y, Gowen BB, Takahashi K, Shiraki K, Smee DF, Barnard DL. Favipiravir (T-705), a novel viral RNA polymerase inhibitor. Antiviral Res. 2013;100:446–454. 10.1016/j.antiviral.2013.09.015 24084488 PMC3880838

[14] Furuta Y, Takahashi K, Fukuda Y, Kuno M, Kamiyama T, Kozaki K, et al In vitro and in vivo activities of anti-influenza virus compound T-705. Antimicrob Agents Chemother. 2002;46:977–981. 11897578 10.1128/AAC.46.4.977-981.2002 PMC127093

[15] Oestereich L, Lüdtke A, Wurr S, Rieger T, Muñoz-Fontela C, Günther S. Successful treatment of advanced Ebola virus infection with T-705 (favipiravir) in a small animal model. Antiviral Res. 2014;105:17–21. 10.1016/j.antiviral.2014.02.014 24583123

[16] MDVI. Phase 3 efficacy and safety study of favipiravir for treatment of uncomplicated influenza in Adults. ClinicalTrials.gov. 2013 Dec 8 [cited 15 Sep 2015]. Available: https://clinicaltrials.gov/ct2/show/NCT02008344.

[17] Madelain V, Oestereich L, Graw F, Nguyen T, de Lamballerie X, Mentré F, et al Ebola virus dynamics in mice treated with favipiravir. Antiviral Res. 2015;123:70–77. 10.1016/j.antiviral.2015.08.015 26343011

[18] Mentre F, Taburet AM, Guedj J, Anglaret X, Keita S, de Lamballerie X, et al Dose regimen of favipiravir for Ebola virus disease. Lancet Infect Dis. 2014;15:150–151. 10.1016/S1473-3099(14)71047-3 25435054

[19] Bouazza N, Treluyer J-M, Foissac F, Mentré F, Taburet A-M, Guedj J, et al Favipiravir for children with Ebola. Lancet. 2015;385:603–604. 10.1016/S0140-6736(15)60232-X 25706078

[20] Rowe AK, Bertolli J, Khan AS, Mukunu R, Muyembe-Tamfum JJ, Bressler D, et al Clinical, virologic, and immunologic follow-up of convalescent Ebola hemorrhagic fever patients and their household contacts, Kikwit, Democratic Republic of the Congo. J Infect Dis. 1999;179:S28–S35. 9988162 10.1086/514318

[21] Ksiazek T, Rollin P, Williams A, Bressler D, Martin M, Swanepoel R, et al Clinical virology of Ebola hemorrhagic fever (EHF): virus, virus antigen, and IgG and IgM antibody findings among EHF patients in Kikwit, Democratic Republic of the Congo, 1995. J Infect Dis. 1999;179:S177–S187. 9988182 10.1086/514321

[22] Towner JS, Rollin PE, Bausch DG, Sanchez A, Crary SM, Vincent M, et al Rapid diagnosis of Ebola hemorrhagic fever by reverse transcription-PCR in an outbreak setting and assessment of patient viral load as a predictor of outcome. J Virol. 2004;78:4330–4341. 15047846 10.1128/JVI.78.8.4330-4341.2004 PMC374287

[23] Kourtis AP, Appelgren K, Chevalier MS, McElroy A. Ebola virus disease: focus on children. Pediatr Infect Dis J. 2015;34:893–897. 10.1097/INF.0000000000000707 25831417 PMC4666536

[24] Rollin PE, Bausch DG, Sanchez A. Blood chemistry measurements and D-dimer levels associated with fatal and nonfatal outcomes in humans infected with Sudan Ebola virus. J Infect Dis2007;196:S364–S71. 10.1086/520613 17940972

[25] Kortepeter MG, Bausch DG, Bray M. Basic clinical and laboratory features of filoviral hemorrhagic fever. J Infect Dis. 2011;204:S810–S816. 10.1093/infdis/jir299 21987756

[26] Bah EI, Lamah M-C, Fletcher T, Jacob ST, Brett-Major DM, Sall AA, et al Clinical presentation of patients with Ebola virus disease in Conakry, Guinea. N Engl J Med. 2015;372:40–47. 10.1056/NEJMoa1411249 25372658

[27] ACAPS. Ebola in West Africa—Guinea: resistance to the Ebola response. ACAPS Thematic Note. 2015 Apr 24 [cited 15 Sep 2015]. Available: http://acaps.org/img/documents/t-acaps_ebola_guinea-resistance-to-ebola-response_24-april-2015.pdf.

[28] Calain P, Poncin M. Reaching out to Ebola victims: coercion, persuasion or an appeal for self-sacrifice? Soc Sci Med. 2015;147:126–133. 10.1016/j.socscimed.2015.10.063 26561947

[29] Presidential Commission for the Study of Bioethical Issues. Ethics and Ebola: public health planning and response. 2015 Feb [cited 28 Jan 2015]. Available: http://bioethics.gov/sites/default/files/Ethics-and-Ebola_PCSBI_508.pdf.

[30] Huang Y, Wei H, Wang Y, Shi Z, Raoul H, Yuan Z. Rapid detection of filoviruses by real-time TaqMan polymerase chain reaction assays. Virol Sin. 2012;27:273–277. 10.1007/s12250-012-3252-y 23001480 PMC8218046

[31] Tam NT, Huy NT, Thoa LTB, Long NP, Trang NTH, Hirayama K, et al Participants understanding of informed consent in clinical trials over three decades: systematic review and meta-analysis. Bull World Health Organ. 2015;93:186–198H. 10.2471/BLT.14.141390 25883410 PMC4371493

[32] Whitehead J. The design and analysis of sequential clinical trials 2nd ed. Chichester: John Wiley & Sons; 1997.

[33] Newcombe R. Two-sided confidence intervals for the single proportion: comparison of seven methods. Stat Med. 1998;17:857–872. 9595616 10.1002/(sici)1097-0258(19980430)17:8<857::aid-sim777>3.0.co;2-e

[34] Hidalgo B, Goodman M. Multivariate or multivariable regression. Am J Public Health. 2013;103:39–40. 10.2105/AJPH.2012.300897 23153131 PMC3518362

[35] Copas J. Plotting p against x. Appl Stat. 1983;32:25–31.

[36] Sissoko S, Folkesson E, Abdoul M, Beavogui A, Gunther S, Shepherd S, et al. Favipiravir in patients with Ebola virus disease: early results of the JIKI trial in Guinea. Abstract 103-ALB. 22nd Conference on Retroviruses and Opportunistic Infections; 23–26 Feb 2015; Seattle, Washington, US. Available: http://www.croiconference.org/sessions/favipiravir-patients-ebola-virus-disease-early-results-jiki-trial-guinea.

[37] Kuhn E, Lavielle M. Maximum likelihood estimation in nonlinear mixed effects models. Comput Stat Data Anal. 2005;49:1020–1038.

[38] Victory KR, Coronado F, Ifono SO, Soropogui T, Dahl BA, Centers for Disease Control and Prevention. Ebola transmission linked to a single traditional funeral ceremony—Kissidougou, Guinea, December, 2014–January 2015. MMWR Morb Mortal Wkly Rep. 2015;64:386–388. 25879897 PMC5779538

[39] Lanini S, Zumla A, Ioannidis JPA, Caro AD, Krishna S, Gostin L, et al Are adaptive randomised trials or non-randomised studies the best way to address the Ebola outbreak in west Africa? Lancet Infect Dis. 2015;15:738–745. 10.1016/S1473-3099(15)70106-4 25881871 PMC7129402

[40] Joffe S. Evaluating novel therapies during the Ebola epidemic. JAMA. 2014;312:1299–1300. 10.1001/jama.2014.12867 25211645

[41] Chimerix. An open-label, multicenter study of the safety and anti viral activity of brincidofovir (BCV, CMX001) for Ebola virus disease. ClinicalTrials.gov. 2014 Oct 7 [cited 15 Sep 2015]. Available: https://clinicaltrials.gov/ct2/show/NCT02271347.

[42] van Griensven J, Edwards T, de Lamballerie X, Semple MG, Gallian P, Baize S, et al Evaluation of Convalescent Plasma for Ebola Virus Disease in Guinea. N Engl J Med. 2016 1 7;374(1):33-42. 10.1056/NEJMoa1511812 26735992 PMC5856332

[43] National Institute of Allergy and Infectious Diseases. Putative investigational therapeutics in the treatment of patients with known Ebola infection. ClinicalTrials.gov. 2015 Feb 13 [cited 15 Sep 2015]. Available at: https://clinicaltrials.gov/ct2/show/NCT02363322.

[44] Qiu X, Wong G, Audet J, Bello A, Fernando L, Alimonti JB, et al Reversion of advanced Ebola virus disease in nonhuman primates with ZMapp. Nature. 2014;514:47–53. 10.1038/nature13777 25171469 PMC4214273

[45] Cooper BS, Boni MF, Pan-ngum W, Day NP, Horby PW, Olliaro P, et al Evaluating clinical trial designs for investigational treatments of Ebola virus disease. PLoS Med. 2015;12:e1001815 10.1371/journal.pmed.1001815 25874579 PMC4397078

[46] Adebamowo C, Bah-Sow O, Binka F, Bruzzone R, Caplan A, Delfraissy J-F, et al Randomised controlled trials for Ebola: practical and ethical issues. Lancet. 2014;384:1423–1424. 10.1016/S0140-6736(14)61734-7 25390318 PMC4392883

[47] Sanchez A, Lukwiya M, Bausch D, Mahanty S, Sanchez AJ, Wagoner KD, et al Analysis of human peripheral blood samples from fatal and nonfatal cases of Ebola (Sudan) hemorrhagic fever: cellular responses, virus load, and nitric oxide levels. J Virol. 2004;78:10370–10377. 15367603 10.1128/JVI.78.19.10370-10377.2004 PMC516433

[48] McElroy AK, Erickson BR, Flietstra TD, Rollin PE, Nichol ST, Towner JS, et al Ebola hemorrhagic fever: novel biomarker correlates of clinical outcome. J Infect Dis. 2014;210:558–566. 10.1093/infdis/jiu088 24526742 PMC4172044

[49] Fitzpatrick G, Vogt F, Moi Gbabai OB, Decroo T, Keane M, De Clerck H, et al The contribution of Ebola viral load at admission and other patient characteristics to mortality in a Medecins Sans Frontieres Ebola case management centre, Kailahun, Sierra Leone, June–October 2014. J Infect Dis. 2015;212:1752–1758. 10.1093/infdis/jiv304 26002981 PMC4633764

[50] Hunt L, Gupta-Wright A, Simms V, Tamba F, Knott V, Tamba K, et al Clinical presentation, biochemical, and haematological parameters and their association with outcome in patients with Ebola virus disease: an observational cohort study. Lancet Infect Dis. 2015;15:1292–1299. 10.1016/S1473-3099(15)00144-9 26271406

[51] Schibler M, Vetter P, Cherpillod P, Petty TJ, Cordey S, Vieille G, et al Clinical features and viral kinetics in a rapidly cured patient with Ebola virus disease: a case report. Lancet Infect Dis. 2015;15:1034–1040. 10.1016/S1473-3099(15)00229-7 26201298

[52] Angus DC, van der Poll T. Severe sepsis and septic shock. N Engl J Med. 2013;369:840–851. 10.1056/NEJMra1208623 23984731

[53] Bellomo R, Kellum JA, Ronco C. Acute kidney injury. Lancet. 2012;380:756–766. 10.1016/S0140-6736(11)61454-2 22617274

[54] Crum-Cianflone NF. Bacterial, fungal, parasitic, and viral myositis. Clin Microbiol Rev. 2008;21:473–494. 10.1128/CMR.00001-08 18625683 PMC2493084

[55] Konrad RJ, Goodman DB, Davis WL. Tumor necrosis factor and coxsackie B4 rhabdomyolysis. Ann Intern Med. 1993;119:861. 10.7326/0003-4819-119-8-199310150-00024 8397486

[56] Naylor C, Jevnikar A, Witt N. Sporadic viral myositis in two adults. CMAJ. 1987;137:819–821. 2832046 PMC1267353

[57] Bosch X, Poch E, Grau JM. Rhabdomyolysis and acute kidney injury. N Engl J Med. 2009;361:62–72. 10.1056/NEJMra0801327 19571284

[58] Ramanan P, Shabman RS, Brown CS, Amarasinghe GK, Basler CF, Leung DW. Filoviral immune evasion mechanisms. Viruses. 2011;3:1634–1649. 10.3390/v3091634 21994800 PMC3187693

[59] Ramanan P, Edwards MR, Shabman RS, Leung DW, Endlich-Frazier AC, Borek DM, et al Structural basis for Marburg virus VP35-mediated immune evasion mechanisms. Proc Natl Acad Sci U S A. 2012;109:20661–20666. 10.1073/pnas.1213559109 23185024 PMC3528546

[60] Broadhurst M, Kelly J, Miller A, Semper A, Bailey D, Groppelli E, et al ReEBOV Antigen Rapid Test kit for point-of-care and laboratory-based testing for Ebola virus disease: a field validation study. Lancet. 2015;386:867–874. 10.1016/S0140-6736(15)61042-X 26119838

[61] Gowen BB, Sefing EJ, Westover JB, Smee DF, Hagloch J, Furuta Y, et al Alterations in favipiravir (T-705) pharmacokinetics and biodistribution in a hamster model of viral hemorrhagic fever. Antiviral Res. 2015;121:132–137. 10.1016/j.antiviral.2015.07.003 26186980 PMC4536130

[62] Varghese JM, Roberts JA, Lipman J. Antimicrobial pharmacokinetic and pharmacodynamic issues in the critically ill with severe sepsis and septic shock. Crit Care Clin. 2011;27:19–34. 10.1016/j.ccc.2010.09.006 21144984

