# Correction: Experimental Treatment with Favipiravir for Ebola Virus Disease (the JIKI Trial): A Historically Controlled, Single-Arm Proof-of-Concept Trial in Guinea

**DOI:** 10.1371/journal.pmed.1002009

**Published:** 2016-04-05

**Authors:** Daouda Sissoko, Cedric Laouenan, Elin Folkesson, Abdoul-Bing M’Lebing, Abdoul-Habib Beavogui, Sylvain Baize, Alseny-Modet Camara, Piet Maes, Susan Shepherd, Christine Danel, Sara Carazo, Mamoudou N. Conde, Jean-Luc Gala, Géraldine Colin, Hélène Savini, Joseph Akoi Bore, Frederic Le Marcis, Fara Raymond Koundouno, Frédéric Petitjean, Marie-Claire Lamah, Sandra Diederich, Alexis Tounkara, Geertrui Poelart, Emmanuel Berbain, Jean-Michel Dindart, Sophie Duraffour, Annabelle Lefevre, Tamba Leno, Olivier Peyrouset, Léonid Irenge, N’Famara Bangoura, Romain Palich, Julia Hinzmann, Annette Kraus, Thierno Sadou Barry, Sakoba Berette, André Bongono, Mohamed Seto Camara, Valérie Chanfreau Munoz, Lanciné Doumbouya, Souley Harouna, Patient Mumbere Kighoma, Fara Roger Koundouno, Réné Lolamou, Cécé Moriba Loua, Vincent Massala, Kinda Moumouni, Célia Provost, Nenefing Samake, Conde Sekou, Abdoulaye Soumah, Isabelle Arnould, Michel Saa Komano, Lina Gustin, Carlotta Berutto, Diarra Camara, Fodé Saydou Camara, Joliene Colpaert, Léontine Delamou, Lena Jansson, Etienne Kourouma, Maurice Loua, Kristian Malme, Emma Manfrin, André Maomou, Adele Milinouno, Sien Ombelet, Aboubacar Youla Sidiboun, Isabelle Verreckt, Pauline Yombouno, Anne Bocquin, Caroline Carbonnelle, Thierry Carmoi, Pierre Frange, Stéphane Mely, Vinh-Kim Nguyen, Delphine Pannetier, Anne-Marie Taburet, Jean-Marc Treluyer, Jacques Kolie, Raoul Moh, Minerva Cervantes Gonzalez, Eeva Kuisma, Britta Liedigk, Didier Ngabo, Martin Rudolf, Ruth Thom, Romy Kerber, Martin Gabriel, Antonino Di Caro, Roman Wölfel, Jamal Badir, Mostafa Bentahir, Yann Deccache, Catherine Dumont, Jean-François Durant, Karim El Bakkouri, Marie Gasasira Uwamahoro, Benjamin Smits, Nora Toufik, Stéphane Van Cauwenberghe, Khaled Ezzedine, Eric D’Ortenzio, Louis Pizarro, Aurélie Etienne, Jérémie Guedj, Alexandra Fizet, Eric Barte de Sainte Fare, Bernadette Murgue, Tuan Tran-Minh, Christophe Rapp, Pascal Piguet, Marc Poncin, Bertrand Draguez, Thierry Allaford Duverger, Solenne Barbe, Guillaume Baret, Isabelle Defourny, Miles Carroll, Hervé Raoul, Augustin Augier, Serge P. Eholie, Yazdan Yazdanpanah, Claire Levy-Marchal, Annick Antierrens, Michel Van Herp, Stephan Günther, Xavier de Lamballerie, Sakoba Keïta, France Mentre, Xavier Anglaret, Denis Malvy, JIKI Study Group

The 103^rd^ author's name is spelled incorrectly. The correct name is: Eric D’Ortenzio.
