# Supplementary figures and images for: Experimental Treatment with Favipiravir for Ebola Virus Disease (the JIKI Trial): A Historically Controlled, Single-Arm Proof-of-Concept Trial in Guinea
Source: PLoS Med. 2016 Mar 1;13(3):e1001967. doi: 10.1371/journal.pmed.1001967 (PMC4773183; doi:10.1371/journal.pmed.1001967)

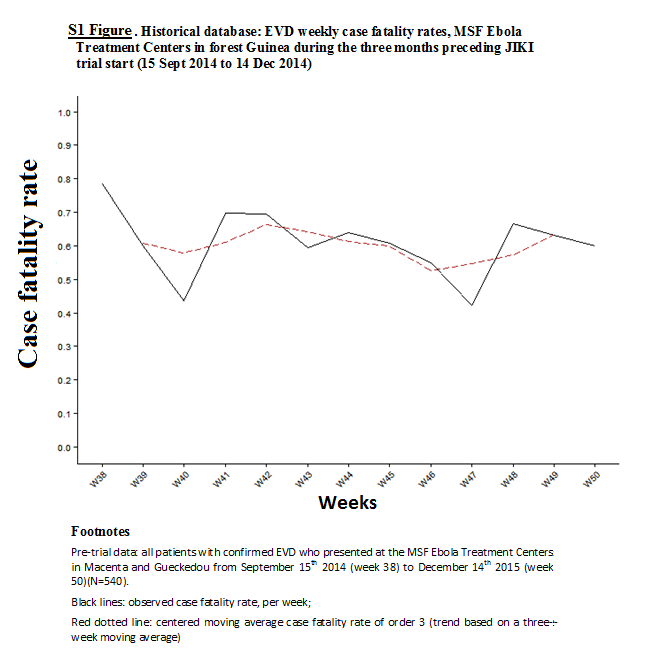

Supplement: S1 Fig — The historical data include all patients with confirmed EVD who presented at the MSF Ebola treatment centers in forested Guinea from 15 September 2014 (week 38) to 14 December 2014 (week 50) (n = 540); the black line is the observed case fatality rate per week; the red dotted line is the centered moving average case fatality rate of order 3 (trend based on a 3-wk moving average). (TIF) [file pmed.1001967.s001.tif]

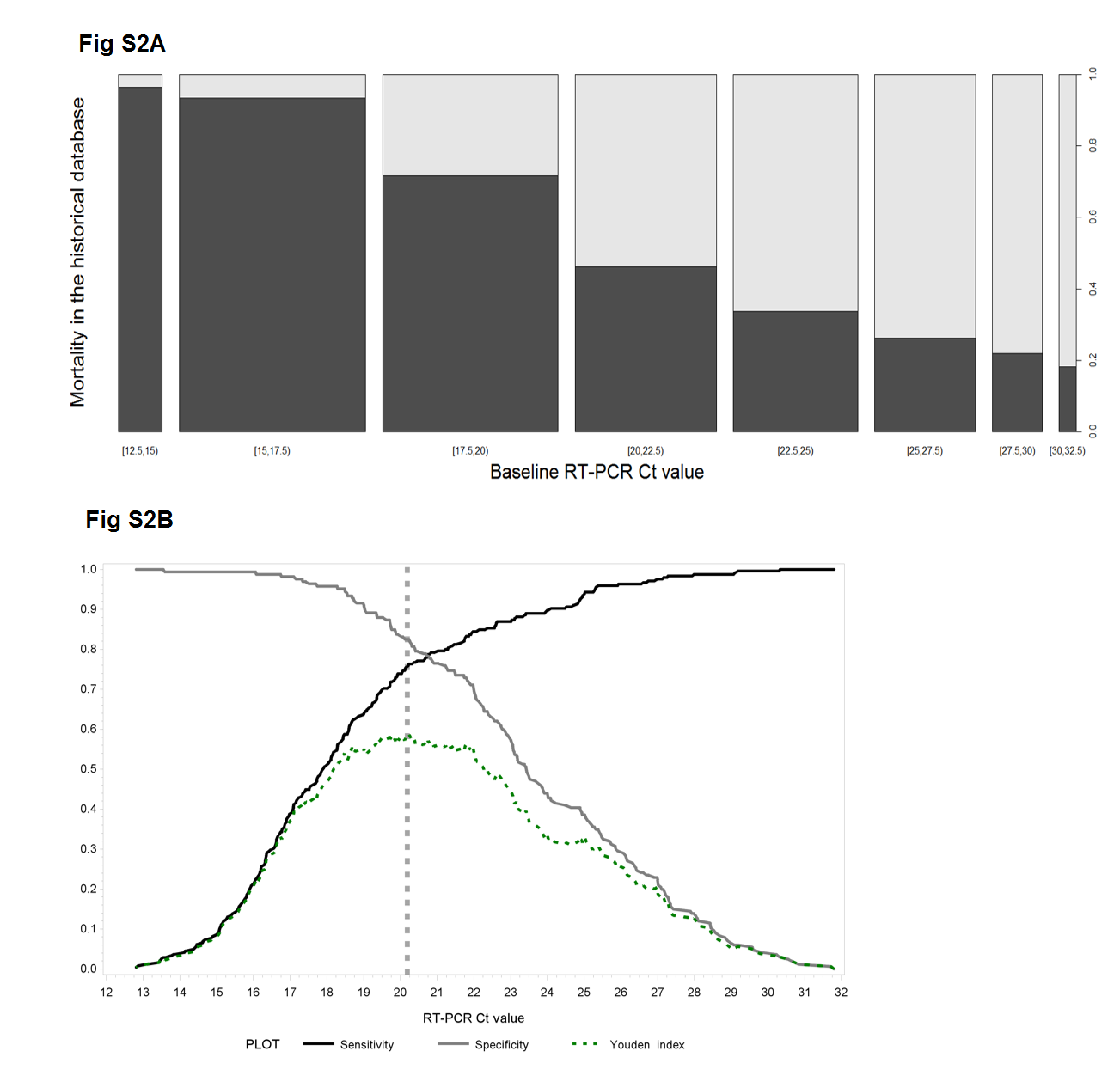

Supplement: S2 Fig — The historical data include all patients with confirmed EVD who presented at the MSF Ebola treatment centers in forested Guinea during the 3 mo preceding JIKI trial start (15 September 2014 to 14 December 2014). (A) Pre-trial mortality rate in each stratum of baseline RT-PCR Ct value. (B) Specificity, sensitivity, and Youden’s index (sensitivity + specificity − 1) of baseline RT-PCR Ct value for mortality. The Youden index was maximum for a baseline RT-PCR Ct value of 20.2. (TIF) [file pmed.1001967.s002.tif]

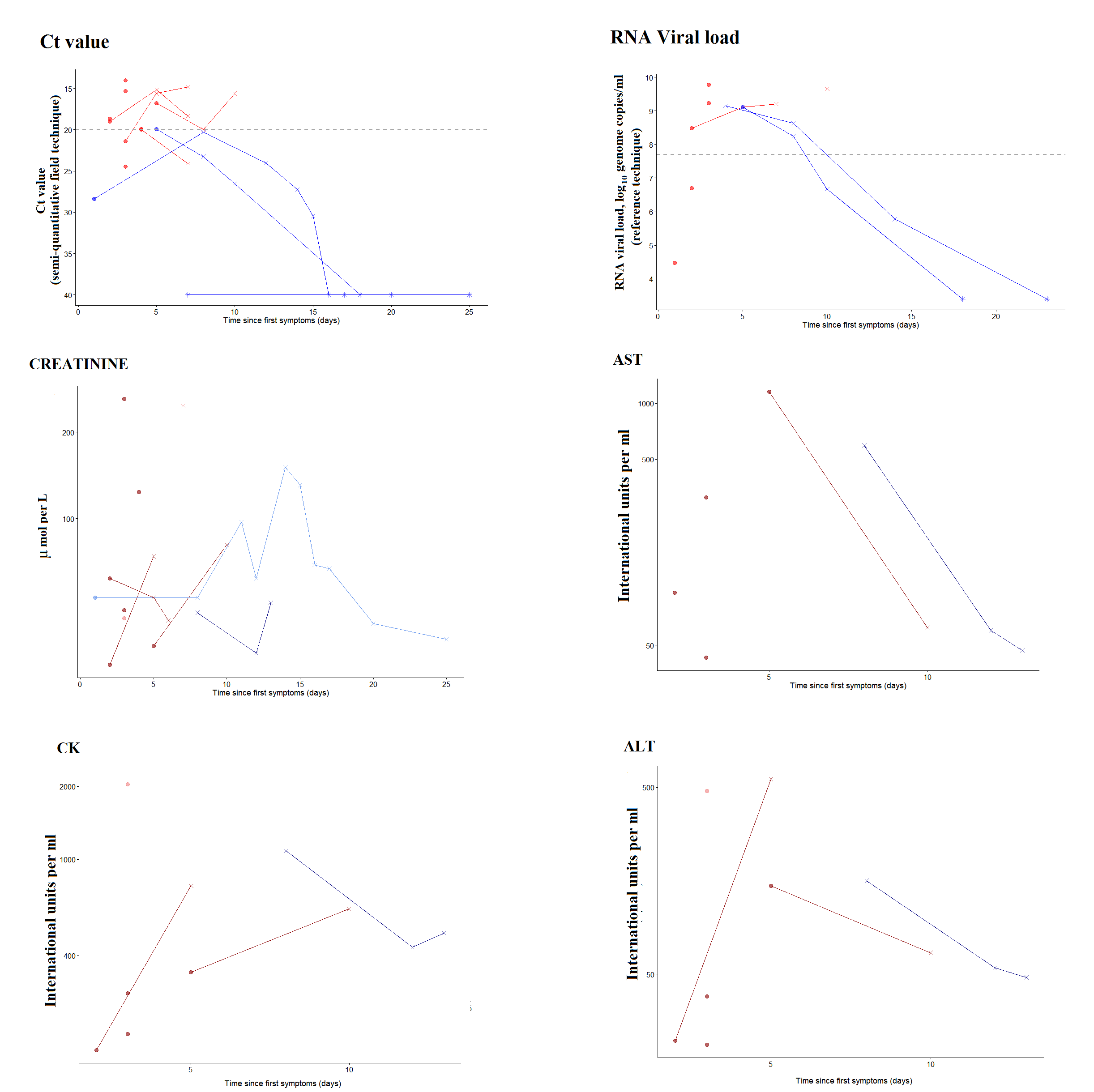

Supplement: S3 Fig — The x-axis represents the time since first symptoms (for example, for a patient whose first symptom occurred 5 d before admission to the treatment center, the baseline value dot is positioned at 5 d). Each line represents one patient. Dots represent baseline values, X’s represent follow-up values, and asterisks represent values below the limit of quantification. In all panels, red symbols represent patients who died, and blue symbols represent patients who survived. Dark red lines represent patients with baseline Ct < 20 who died, light red lines represent patients with baseline Ct ≥ 20 who died, dark blue lines represent patients with baseline Ct < 20 who survived, and light blue lines represent patients with baseline Ct ≥ 20 who survived. Lines with no initial dot represent patients with unavailable baseline values (only follow-up values are available). Samples obtained more than 25 d after onset of symptoms are not represented. (TIF) [file pmed.1001967.s003.tif]
