# Supplementary material for: Experimental Treatment with Favipiravir for Ebola Virus Disease (the JIKI Trial): A Historically Controlled, Single-Arm Proof-of-Concept Trial in Guinea
Source: PLoS Med. 2016 Mar 1;13(3):e1001967. doi: 10.1371/journal.pmed.1001967 (PMC4773183; doi:10.1371/journal.pmed.1001967)
Supplement: S1 Table — (PDF) [file pmed.1001967.s004.pdf]

<u>S1Table</u>. JIKI Trial Preparation: Statistical power provided by a sample size of 60 participants to detect a difference between mortality in the trial and a target value, according to different hypotheses of target values and of on-trial mortality

Target value	Absolute difference between on-trial mortality and target value		
	-10%	-15%	-20%
60%	OTM: 50%<br>Pw: 36%	OTM: 45%<br>Pw: 66%	OTM: 40%<br>Pw: 89%
50%	OTM: 40%<br>Pw: 34%	OTM: 30%<br>Pw: 65%	OTM: 30%<br>Pw: 89%
40%	OTM: 30%<br>Pw: 34%	OTM: 25%<br>Pw: 68%	OTM: 20%<br>Pw: 93%
30%	OTM: 20%<br>Pw: 38%	OTM: 15%<br>Pw: 77%	OTM: 10%<br>Pw: 98%

Footnotes to S1Table:

OTM: On-trial mortality; Pw: Statistical power.

Interpretation: with N = 60 participants, with a two-tailed α of 5%, the statistical power (1-β) was greater than 80% to detect that on-trial mortality (TM) was -20% lower than the target value (within a range a target values from 30% to 60%).
