# Supplementary material for: Experimental Treatment with Favipiravir for Ebola Virus Disease (the JIKI Trial): A Historically Controlled, Single-Arm Proof-of-Concept Trial in Guinea
Source: PLoS Med. 2016 Mar 1;13(3):e1001967. doi: 10.1371/journal.pmed.1001967 (PMC4773183; doi:10.1371/journal.pmed.1001967)
Supplement: S2 Table — (PDF) [file pmed.1001967.s005.pdf]

S2 Table. Historical database: characteristics of patients with EVD hospitalized in MSF Ebola treatment centers in forest Guinea during the three months preceding JIKI trial start (15 Sept 2014 to 14 Dec 2014)

	Young Children <6 years	Adults and Children ≥6 years
Number of patients in database	62	478
**Baseline characteristics**		
Female sex, n (%)*	34 (55)	242 (51)
Age (years), median (IQR)	3 (1 to 5)	32 (24 to 49)
Time from first symptoms to admission (days), median (IQR) †	3 (2 to 5)	5 (3 to 7)
< 3 days, n (%)	36 (58)	145 (30)
≥ 3 days, n (%)	26 (42)	333 (70)
EBOV RT-PCR Ct value, median (IQR)	20.8 (17.4 to 25.1)	20.1 (17.2 to 23.9)
< 20, n (%)	29 (47)	232 (49)
[20-25[, n (%)	17 (27)	154 (32)
≥ 25, n (%)	16 (26)	92 (19)
**Outcome**		
Survived, n (%)	19 (30.6)	206 (43.1)
Died, n (%)	43 (69.4)**	272 (56.9)
Death by baseline Ct value		
< 20, n (%)	23/29 (79.3)	197/232 (84.9) ††
[20-25[, n (%)	12/17 (70.6)	57/154 (37.0) †††
≥ 25, n (%)	8/16 (50.0)	18/92 (19.6) †††

Footnotes to S2Table:

* 1 missing value;
† 4 outlier values (>20 days) were imputed to 20 days;
**42 out of the 43 (98%) deaths in children <6years occurred within 14 days
†† all 197 deaths (100%) in patients with baseline Ct ≤20 occurred within 14 days
††† 71 out of the 75 (95%) deaths in patients with baseline Ct ≥20 occurred within 14 days, and 75 out of 75 (100%) occurred within 21 days;
Ct: cycle threshold, RealStar® Filovirus Screen RT-PCR kit 1.0, Altona Diagnostic
