# Supplementary material for: Experimental Treatment with Favipiravir for Ebola Virus Disease (the JIKI Trial): A Historically Controlled, Single-Arm Proof-of-Concept Trial in Guinea
Source: PLoS Med. 2016 Mar 1;13(3):e1001967. doi: 10.1371/journal.pmed.1001967 (PMC4773183; doi:10.1371/journal.pmed.1001967)
Supplement: S3 Table — (PDF) [file pmed.1001967.s006.pdf]

S3 Table. Historical database: Factors associated with mortality in patients with EVD hospitalized in MSF Ebola treatment centers in forest Guinea during the three months preceding JIKI trial start (15 Sept 2014 to 14 Dec 2014)

	N	Death		Univariable			Multivariable		
		n	(%)	OR	[95%CI]	*P*	aOR	[95%CI]	*P*
**Sex***									
Male	263	155	(58.9)	1.1	[0.8-1.5]	0.8	1.1	[0.8-1.7]	0.5
Female	276	159	(57.6)	1			1		
**Time first symptoms-admission (days)**†									
≤3	181	109	(60.2)	1.1	[0.8-1.6]	0.5	1.2	[0.7-1.8]	0.5
>3	359	206	(57.4)	1					
**Baseline RT-PCR CT value**									
<20	261	220	(84.3)	10.4	[6.9-15.7]	$<10^{-16}$	11.1	[7.2-17.1]	$<10^{-16}$
≥20	279	95	(34.1)	1			1		
**Age (years)**									
≤6	62	43	(69.4)	3.0	[1.6-5.5]	0.0005	3.8	[1.9-7.9]	0.0002
>6 and <30	192	83	(43.2)	1			1		
≥30	286	189	(66.1)	2. 6	[1.8-3.7]	$<10^{-6}$	2.9	[1.8-4.5]	$<10^{-6}$

Footnotes to S3 Table:

* 1 missing value; † 4 outlier values (>20 days) were imputed to 20 days; Ct: cycle threshold, RealStar® Filovirus Screen RT-PCR kit 1.0, Altona Diagnostics; OR, Odds Ratio; aOR, adjusted Odds Ratio; CI: confidence interval.
