# Supplementary material for: Experimental Treatment with Favipiravir for Ebola Virus Disease (the JIKI Trial): A Historically Controlled, Single-Arm Proof-of-Concept Trial in Guinea
Source: PLoS Med. 2016 Mar 1;13(3):e1001967. doi: 10.1371/journal.pmed.1001967 (PMC4773183; doi:10.1371/journal.pmed.1001967)
Supplement: S4 Table — (PDF) [file pmed.1001967.s007.pdf]

**S4 Table. JIKI trial: Baseline and follow-up characteristics in adult and adolescents who died and in those who survived**

| | N | Died (n=51) | | Survived (n=48) | | *p* |
|---|---|---|---|---|---|---|
| ***Baseline*** | | | | | | |
| Sex, female, n (%) | 99 | 31 | (61) | 32 | (67) | 0.68 |
| Age (years) , median (IQR) | 99 | 40 | (28-55) | 31 | (19-47) | 0.05 |
| Mode of contamination, n (%) | 99 | | | | | 0.25 |
| Funerals | | 30 | (59) | 20 | (42) | |
| Contact with EVD patient | | 20 | (39) | 24 | (50) | |
| Unknown | | 1 | (2) | 4 | (8) | |
| RT-PCR Ct value, median (IQR) | 99 | 17.7 | (16.3-19.5) | 23.8 | (21.0-26.9) | <0.001 |
| Viral load ($log_{10}$ copies/ml), median (IQR) | 84 | 8.4 | (7.8-9.1) | 6.4 | (5.8-7.5) | <0.001 |
| Positive malaria rapid test, n (%) | 97 | 5 | (10) | 6 | (13) | 0.75 |
| Creatinine (μmol/L), median (IQR) | 91 | 285 | (134-521) | 108 | (79-165) | <0.001 |
| BUN (mmol/l), median (IQR) | 91 | 14.6 | (8.4-25.9) | 6.8 | (4.3-12.2) | <0.001 |
| BUN:creatinine ratio, median (IQR) | 91 | 0.05 | (0.04-0.07) | 0.06 | (0.04-0.08) | 0.22 |
| AST (IU/l), median (IQR) | 46 | 1312 | (232-2000) | 374 | (139-1002) | 0.12 |
| ALT (IU/ l), median (IQR) | 54 | 344 | (160-536) | 130 | (74-320) | 0.05 |
| CK (IU/l), median (IQR) | 53 | 2119 | (735-4099) | 1176 | (590-2977) | 0.31 |
| Bilirubin (mg/dl), median (IQR) | 51 | 12.0 | (9.0-19.0) | 10.5 | (9.0-11.5) | 0.08 |
| Amylase (IU/l), median (IQR) ¶ | 53 | 133.0 | (96.0-185.0) | 92.0 | (71.0-141.0) | 0.11 |
| CRP (mg/l), median (IQR) ¶ | 51 | 29.5 | (14.2-58.6) | 15.2 | (5.0-37.4) | 0.11 |
| Albumin (g/l), median (IQR) ¶ | 53 | 30.0 | (29.0-36.0) | 30.0 | (27.5-34.5) | 0.93 |
| Sodium (mmol/L), median (IQR) | 86 | 132 | (128-135) | 132 | (129-136) | 0.92 |
| Potassium (mmol/L), median (IQR) | 84 | 3.8 | (3.5-4.6) | 3.9 | (3.4-4.3) | 0.87 |
| Glucose (mmol/L), median (IQR) | 83 | 5.75 | (4.42-6.80) | 5.8 | (4.88-6.99) | 0.72 |
| ***Treatment*** | | | | | | |
| Time from first symptoms to favipir. initiation (days), median (IQR) | 99 | 5 | (3-7) | 5 | (3-7) | 0.53 |
| IV fluid rehydration, n (%) | 99 | 50 | (98) | 41 | (85) | 0.03 |

**<u>S4 Table</u> (continued). JIKI trial: Baseline and follow-up characteristics in adult and adolescents who died and in those who survived**

| | N | Died (n=51) | | Survived (n=48) | | *p* |
|---|---|---|---|---|---|---|
| ***Signs and symptoms during hospital stay*** | | | | | | |
| Maximal temperature (°C), median (IQR) | 99 | 39.3 | (38.8-40.0) | 39.1 | (38.1-39.9) | 0.17 |
| Vomiting, maximal grade, n (%) | 99 | | | | | 0.16 |
| 0 | | 9 | 18% | 3 | 6% | |
| 1 | | 28 | 55% | 34 | 71% | |
| 2 | | 10 | 20% | 6 | 13% | |
| 3 | | 4 | 8% | 1 | 2% | |
| Diarrhea, maximal grade, n (%) | 99 | | | | | 0.003 |
| 0 | | 0 | 0% | 2 | 4% | |
| 1 | | 6 | 12% | 18 | 38% | |
| 2 | | 16 | 31% | 14 | 29% | |
| 3 | | 29 | 57% | 14 | 29% | |
| Impaired consciousness, maximal grade, n (%) | 99 | | | | | <0.001 |
| 0 | | 20 | 39% | 42 | 88% | |
| 1 | | 25 | 49% | 5 | 10% | |
| 2 | | 3 | 6% | 1 | 2% | |
| 3 | | 3 | 6% | 0 | 0% | |
| Any pain, maximal grade, n (%) | 99 | | | | | <0.001 |
| 0 | | 1 | 2% | 0 | 0% | |
| 1 | | 26 | 51% | 44 | 92% | |
| 2 | | 18 | 35% | 3 | 6% | |
| 3 | | 6 | 12% | 1 | 2% | |
| Any hemorrhage, at least once, n (%) | 99 | 36 | 71% | 21 | 44% | 0.01 |
| Hiccup, at least once, n (%) | 99 | 8 | 16% | 9 | 19% | 0.79 |
| Dehydration, at least once, n (%) | 99 | 41 | 80% | 30 | 63% | 0.07 |
| Icterus, n (%) | 99 | 6 | 12% | 4 | 8% | 0.74 |
| Edema, at least once, n (%) | 99 | 7 | 14% | 7 | 15% | 1.00 |

## Footnotes to S4 Table:

N=number of patients with available value. For biochemical tests, all four trial centers, systematicly measured creatinine, BUN, sodium, potassium and glucose. Three of the four centers (Gueckedou, Macenta, Conakry), also measured serum AST, ALT, total bilirubin, amylase, albumin, CK and C-RP.

BUN: Blood urea nitrogen ; AST: aspartate aminotransferase. ALT: alanine aminotransferase; CK: creatine phosphokinase; CRP: C-reactive protein; IU: international units. Favipir.: favipiravir.

1$^{st}$ sympt-FVP : time between the onset of first symptom and first dose of favipiravir

Signs and symptoms were documented twice a day during the overall hospital stay. Diarrhea, consciousness, pain, and vomiting were graded 0 to 3; for other signs/symptoms, only the presence or absence of the sign/symptom (yes/no) was noted.
