# Supplementary material for: Experimental Treatment with Favipiravir for Ebola Virus Disease (the JIKI Trial): A Historically Controlled, Single-Arm Proof-of-Concept Trial in Guinea
Source: PLoS Med. 2016 Mar 1;13(3):e1001967. doi: 10.1371/journal.pmed.1001967 (PMC4773183; doi:10.1371/journal.pmed.1001967)
Supplement: S1 Text — (PDF) [file pmed.1001967.s009.pdf]

ITMO Santé publique

Inserm

Institut national
de la santé et de la recherche médicale

RESEARCH PROTOCOL

Title: Efficacy of favipiravir in reducing mortality in individuals with Ebola Virus Disease in Guinea

Short title: JIKI

N° Promotor	N° clinicaltrials.gov
C14-63	NCT02329054

Approuved by National ethic comity of Guinea on 28/11/2014
Approuved by the Inserm IRB 00003888/FWA00005831, France on 25/11/2014
Approuved by the MSF ERB on 17/12/2014

VERSION N°1.6 - 03/03/2015

Sponsor:
Inserm - ITMO Santé Publique - Pôle Recherche Clinique (PRC)
Biopark, Bâtiment A, 8 rue de la Croix Jarry, 75013 Paris
Contact:
LEVY-MARCHAL Claire, Chef de projet Inserm
Email: claire.levy-marchal@inserm.fr
Tel: +331 44 23 67 55 / Fax: +331 44 23 67 10

Principal Investigators:
1/ Pr Denis Malvy and 2/ Dr Sakoba Keita
Institute/Organization/Address:
1/ Head of Infectious diseases department, Bordeaux Univ Hospital & Inserm unit 897; Centre Inserm 897, ISPED, Université de Bordeaux, F-33076 Bordeaux cedex, France;
2/ Guinea's national coordinator for the fight against Ebola, Ministère de la Santé, Conakry, Guinée.
Tel: 1/ +33 (0)6 85945772 ; 2/ +224 622931390
Email: denis.malvy@chu-bordeaux.fr ; keita_sakoba@yahoo.fr

Scientific Managers:
1/ Dr Xavier Anglaret and 2/ Pr France Mentré
Institute/Organization/Address:
1/ Inserm unit 897, ISPED, Université de Bordeaux, F-33076 Bordeaux cedex, France & Programme PACCI/site ANRS, Abidjan, Côte d'Ivoire.
2/ Inserm unit 1137 IAME, Université Paris Diderot, 16 rue Henri Huchard, F-75018 Paris, France.
Tel: 1/ +33 557571258 ; 2/ +33 157277759
Email: xavier.anglaret@isped.u-bordeaux2.fr ; france.mentre@inserm.fr

Table of Contents

1. Synopsis 4

2. Justification 7

2.1 Assessing treatment for Ebola: an adaptive process 7

2.2 Study design issues 8

2.3 The importance of earliness 8

2.4 Children 8

2.5 Influence of baseline viral load on mortality 9

2.6 Dose issues 9

2.6.1 Adults 9

2.6.2 Children 10

2.7 Treatment duration 10

2.8 Toxicity issues 11

2.8.1 Adults 11

2.8.2 Children 11

2.9 Context implications 11

3. Objectives 12

4. Study design 12

5. Study sites 12

6. Participants 13

6.1 Inclusion and non-inclusion criteria 13

6.2 Groups 13

6.3 Sample size and power 13

7. Outcomes 14

8. Trial conduct 14

8.1 Sponsor and coordination 14

8.2 Trial drug 14

8.3 Trial development and scheduled assessment 14

8.4 Biology 16

8.5 Trial management 17

8.5.1 Monitoring 17

8.5.2 Data management 17

8.5.3 Pharmacovigilance and safety management 18

8.6 Study medication manufacturing, packaging and shipping 18
8.7 Trial surveillance 18
8.7.1 Safety monitoring board 18
8.7.2 Steering Committee and Scientific Advisory Board 19
9. Statistical analysis 19
9.1 Mortality 19
9.2 Other outcomes 19
9.3 Intermediate analyses and stopping rules for futility 20
10. Trial termination 20
11. Ethical and regulatory issues 20
11.1 Communication with regulatory authorities and ethics committees 20
11.2 Informed consent 21
11.3 Safety 21
11.4 Social aid 21
11.5 Partnership 21
11.6 Community involvement 22
11.7 Communication of results 23
12. Timeline 23
13. References 24

1. Synopsis

Objective: to assess the efficacy of high-dosed favipiravir in reducing mortality in humans with Ebola Virus Disease (EVD).

Hypotheses:
1) The efficacy of antivirals in patients with EVD should correlate negatively with time since first symptoms. Thus, in this proof of concept trial, the main analysis will be done in adult patients with early symptoms in whom the efficacy is expected to be the highest;
2) Favipiravir for EVD should be given at higher doses than that previously tested in humans with influenza. For this trial, the dose was calculated based on pharmacokinetics simulations, to rapidly reach plasma concentrations associated with anti-EBOV activity.
3) A third assumption was made, given recent pre-trial data showing first a strong link between baseline viral load and mortality and second a higher mortality in children below 6 years: the efficacy of antivirals in patients with EVD should correlate negatively with viral load at start of treatment and with young age. Thus a secondary objective is the efficacy of favipiravir in adult patients and children >6 years with moderate baseline viral load (i.e. cycle threshold [Ct] ≥20 measured by RT-PCR) in whom the efficacy is expected to be the highest.

Design: non-comparative, proof-of-concept, phase II trial.

Settings: Several EVD care centers in Guinea.

Inclusion criteria: age ≥1 year and weighting ≥ 10kg, EVD confirmed by a positive qualitative PCR test, signed informed consent.

Group definition: Group A1: adults ≥18 years with time between first symptoms and first dose of favipiravir ≤72h; group A2: adults ≥18 years with time between first symptoms and first dose of favipiravir >72h; group C: all children ≥1 year.
The division in groups is a matter of analysis, and will not be perceptible during the trial process by the patients. Patients in the three groups will receive the same treatment and will be followed with the same procedures Daily dosages will be adapted to the body weight in group C.

Given recent pre-trial data, a second definition of groups was also used: group AC1: adults and children >6 years with baseline Ct ≥20; group AC2: adults and children >6 years with baseline Ct values <20; group YC: young children ≤6 years and ≥1 year.

Non inclusion-criteria: pregnancy*, inability to take the drug (encephalopathy; severe vomiting). *(*for pregnant women, see the ethics section).*

Primary outcome: mortality by Day-14 Day-0 is the day of the first dose of favipiravir.

Secondary outcomes: mortality by Day-14 according to the second group definition (AC1, AC2, YC), evolution of EBOV plasma RNA and infectious loads; grade 3-4 adverse events; viral micro-diversity of EBOV (including resistance mutations); trough concentrations of favipiravir; criteria for cure at Day-30.

Trial treatment: Favipiravir (oral administration, 200 mg light yellow, round-shaped, coated divisible tablets that can be crushed and mixed with liquid):

- Adults (groups A1 and A2): Day-0, h0: 2400 mg; h8: 2400 mg; h16: 1200 mg. Day-1 to Day-9: 1200 mg bid.
- Children (group C): dosages will be adapted to the body weight:

Weight (kg)	Day-0 (mg)			Day-1 to Day-9 (mg)		
	0h	8h	16h	0h	8h	16h
**10 - 15**	500	500	200	200	200	200

	Day-0 (mg)			Day-1 to Day-9 (mg)	
	**0h**	**8h**	**16h**	**0h**	**12h**
**16 - 21**	800	800	400	400	400
**22 - 35**	1200	1200	600	600	600
**36 - 45**	1600	1600	800	800	800
**46 - 55**	2000	2000	1000	1000	1000
**> 55 (adult)**	2400	2400	1200	1200	1200

From Day-0 to end of follow-up, all participants will receive the standard package of care, as defined by the care providers. This will be the object of a data collection, but will not be imposed by the protocol.

Follow-up: All patients will be followed a minimum of 30 days. They will be hospitalized in the EVD care center until Day-14, including those who reach criteria for cure before Day-14. Patients who meet criteria for cure before Day-14 or at Day-14 will be discharged at Day-14. Other patients will be hospitalized until criteria for cure are met. Patients discharged before Day-30 will be asked to show up at the EVD care center at Day-30 for the end-of-follow-up visit.

Criteria for cure will be the following:
- 4 days without fever or significant symptoms AND;
- able to feed and walk independently AND;
- two consecutive negative PCR.

Blood collection:

- Adults (groups A1 and A2): blood will be collected in 4 ml trial tubes during: (i) routine care venepuncture (Day-0; end of symptoms (EOS)+72h); (ii) additional trial venepuncture at: Day-2, Day-4, Day-13 and Day-30.
- Children (group C): blood will be collected in 4 ml trial tubes during: (i) routine care venepuncture (Day-0, EOS+72h); (ii) additional trial venepuncture at Day-2 Day-4, Day-13 and Day-30.

Biology: *In trial settings laboratories*: At Day-0, Day-2 Day-4, Day-13 the following tests will be done: qualitative PCR (Altona qualitative RT-PCR commercial kit); and biochemical tests (creatininemia and others tests according to local choice), if biochemical tests are doable in the laboratory. At Day-30 the following tests will be done: biochemical tests (creatininemia and others tests according to local choice) only if tests at Day-13 were abnormal *Outside trial settings laboratories*: all trial tube will be shipped frozen in triple-layer packaging to the Lyon-Mérieux BSL4 laboratory, where the following tests will be done: quantitative PCR (standard real-time RT-PCR procedures, synthetic RNA quantified external calibrators), infectious viral load (standard procedure with serial sera dilutions and infection of Vero cells), viral subpopulations (next-generation sequencing methods), drug concentrations measurement (HPLC), uricemia, and other biochemical tests (transaminases, creatininemia, natremia and kaliemia), if biochemical tests have not been done in the trial setting laboratory.

Sample size: recruitment in the trial will be kept opened in all groups* until group A1 reaches 60 participants *(*unless early termination is recommended by the DSMB).*

Statistical analysis for primary outcome: mortality by Day-14 with exact 95%CI will be reported overall and in each group separately.

In group A1, if the number of deaths is <24 (40%) out of 60 participants, the upper bound of the 95%CI will be <55%.

In group A2, if the number of deaths is <54 (45%) out of 120 participants, the upper bound of the 95%CI will be <55%.

In group C, if the number of deaths is <17 (38%) out of 45 participants, the upper bound of the 95%CI will be <55%.

In each group (A1, A2, C), we will conclude that favipiravir decreases mortality if the upper bound of the 95% CI does not include the observed pre-trial mortality (55%) in untreated patients with same duration of symptoms prior to trial initiation.

Statistical analysis for secondary outcomes: mortality by Day-14 with exact 95%CI will be reported according to the second group definition (AC1, AC2, YC). In each group, we will conclude that favipiravir decreases mortality if the upper bound of the 95% CI does not include the observed pre-trial mortality (30%, 85% and 70%, respectively) in untreated patients with same characteristics prior to trial initiation.

All the following outcomes will be analysed overall and separately by the two group definitions (A1, A2, C or AC1, AC2, YC): the evolution of EBOV viral load between Day-0 and end of follow-up will be described in each patient; the numbers, proportions and exact 95% CI will be reported for grade 3-4 clinical or biological adverse events, resistance mutations and criteria for cure at Day-30; mean and inter and intra patient variability of trough concentrations of favipiravir will be described; factors associated with mortality by Day-14, cure at Day-30 and grade 3-4 clinical or biological adverse events (including evolution of viral load, time since first symptoms, trough concentrations of favipiravir) will be studied.

Stopping rules for futility: there will be intermediate analyses of mortality for futility only, performed every 20 adults in each group and every 10 children:
- **Adults**: recruitment in the two groups A1 and A2 will be prematurely stopped if the results show that the trial is unlikely to prove that mortality with favipiravir is <55%;
- **Children**: recruitment in the group C will be prematurely stopped if the results show that the trial is unlikely to prove that mortality with favipiravir is <55%.
Toxicity: deaths will be reported to the sponsor and to the DSMB on a daily basis; SAE other than death will be reported to the sponsor and to the DSMB on a weekly basis.

Surveillance: investigators, sponsor, SAB and DSMB will be closely coordinated, so that new facts can be discussed rapidly and the research plan (epidemiological data, design, dosage, drugs, combinations) adapted accordingly.

Ethics: the final protocol version (v1.2) will be submitted for approval to three ethics committees: the Inserm IRB, the MSF ethics committee, and the Guinean ethics committee for health research. The Inserm IRB already approved a previous version (v0.4), and strongly recommended that pregnant women with EVD receive favipiravir. As the drug is not recommended during pregnancy, the sponsor could not find any insurance company keen to insure pregnant women participating in the trial. Given the outcomes of EVD in pregnant women (death or miscarriage), the benefit/risk ratio of receiving the drug has been deemed to be likely positive for both the mother and the baby. Thus, emergency use of favipiravir in pregnant women outside of the trial is envisaged and under evaluation.

Further steps: This trial is the first phase of a research aiming at finding the best antiviral treatment for patients with EVD. There are three scenarios for the outcome of this trial: (i) the trial is not terminated preliminarily by the DSMB and the conclusions suggest that favipiravir reduces mortality; (ii) the trial is not terminated preliminarily by the DSMB and the conclusions do not suggest that favipiravir reduces mortality; (iii) the trial is terminated preliminarily by the DSMB for futility. Depending on the results of this first phase, further phases will consist of replacing favipiravir with another drug (e.g. brincidofovir) or intervention (e.g. convalescent plasma), or combining drugs and/or interventions. Regardless of the scenario, further steps will be prepared ahead of study termination, so that there is no gap.

2. Justification

2.1 Assessing treatment for Ebola: an adaptive process

There is no specific treatment for Ebola Virus Disease (EVD). Current EVD care are supportive, and includes intravenous or oral rehydration, nutrition, pain killers, treatment of coinfections with antibacterial and antimalarial drugs, and blood transfusion when appropriate. Despite these interventions, mortality remains high (Feldmann *et al*. 2011; Fowler *et al*. 2014; Baize *et al*. 2014).

Moreover, recent epidemiological data showed that children accounted for a significant proportion of patients admitted to the West-African medical centers for EVD. For example, 3.6-6.3% and 7.5-17.4% of patients recently admitted to the MSF Ebola management centers were <5 and 5-15 year old children, respectively. Moreover, the mortality reported in patients admitted in MSF treatment centers for a confirmed EVD seems to be significantly higher in young children (<5 years) compared to the 5-40 year old patients (unpublished data). Therefore, there is an urgent need to study the efficacy of potential anti-Ebola specific interventions, not only in adults, but also in the pediatric population.

Potential anti-Ebola specific interventions include convalescent plasma, monoclonal and polyclonal antibodies, small inhibitory RNA (siRNA), synthetic adenosine analogues or RNA polymerase inhibitors. All these interventions are considered investigational due to lack of data in humans with EVD.

In this study, we chose to study the efficacy of favipiravir because this drug:
- showed anti-Ebola efficacy in immunodeficient murine models;
- has been studied in thousands of adult humans participating in anti-influenza trials, with good tolerance; it has been approved for treating novel or resistant influenza infections in Japan (Appendix 1: favipiravir efficacy);
- is immediately available;
- can be used orally, and can be easily given in both adults and children because pills can be crushed and mixed in food or liquids;
- has recently been used in Europe for treating several patients with EVD; the French drug safety agency (ANSM) has reviewed published data as well as data provided by the firm (Toyama Chemical Co., Ltd) and approved its compassional use in EVD.

Appendix 2 shows current knowledge on other potential therapies for EVD.

Knowledge on the best treatment to use and/or the availability of treatments may evolve rapidly. For example, new data may become available on the feasibility and medical effectiveness for collection and use of convalescent plasma that has been prioritized by WHO; or, ZMapp, a combination of 3 monoclonal antibodies that has been tested in animals and in several patients since the beginning of the current epidemic but is currently no more available, may become available (Qiu X *et al*. 2014; Olinger GG Jr *et al*. 2012); or pre-clinical toxicology and phase 1 data regarding BCX4430, a nucleoside analog that blocks viral RNA synthesis, with promising results in rodents and monkeys, may become available (Warren TK *et al*. 2014).

In the present trial JIKI (means "Hope" in Kissi language), investigators, sponsor, scientific advisory board and safety monitoring board will be coordinated in a very reactive way, so that any new fact can be discussed rapidly and the research plan can be adapted accordingly (change in drug dosage; use of drug combination; combination with another strategy such as passive immunization with convalescent plasma, etc.).

2.2 Study design issues

A comparative trial of favipiravir against a standard package of care (with or without placebo) has been deemed not appropriate because of: i) the highly sensitive social and political context; ii) the need to collect rapidly basic phase II evidence on the efficacy of high dosed favipiravir on EVD before choosing the best intervention(s) to be tested in phase III (favipiravir alone or in combination with other drugs; other therapeutic options including convalescent plasma).

Therefore, we propose a non-comparative, proof-of-concept, phase II trial in patients with EVD, which will allow concluding within a few weeks:

- **that mortality in patients starting favipiravir within 72 hours after the onset of first symptoms is inferior to mortality without treatment prior to the trial initiation;**
- **or that there is no trend that favipiravir brings significant benefit in terms of survival.**

2.3 The importance of earliness

The efficacy of antivirals in acute viral infections is optimal when used at the early stages of the disease, as evidenced by previous studies using neuraminidase inhibitors for the treatment of influenza. This corresponds to situations where the ratio (antiviral molecules available)/(viral targets) is high. When the treatment is initiated later in the course of the disease, it might be impossible to reach similar ratio values and the impact on the kinetics of virus propagation is likely to be less significant.

A first important consequence is that the efficacy of any antiviral in reducing EVD mortality is expected to correlate negatively to the delay between first symptoms and treatment initiation. Thus in this trial, all patients with the exception of pregnant women, with a qualitative PCR positive to EVD, able to take the drug and giving informed consent (in case of children, consent will be given by the parents/adult guardians) will receive the treatment, irrespective of the duration of symptoms prior to admission. However, groups will be constituted according to age and to the duration of symptoms: Group A1: adults with time between first symptoms and first dose of favipiravir ≤72h; Group A2: adults with time between first symptoms and first dose of favipiravir >72h and Group C: all children ≥1 year and weighting ≥10kg. Because the number of potentially eligible children is expected to be lower than that of adults, all children will be included in the same group irrespective of the duration of symptoms. However, the duration of symptoms will be taken into account as a continuous covariable in the pediatric main analysis, and the main analysis will be done in Group A1. The division in groups is a matter of analysis, and will not be perceptible by the patients during the trial process.

A second important consequence is that the treatment should be provided as soon as possible to patients with EVD confirmed by a positive qualitative PCR test. Therefore, particular efforts should be made to shorten the time between first symptoms and blood collection, as well as the time between blood collection and the result of the test.

A third important consequence is that the medication loading dose should allow reaching efficient anti-Ebola plasma concentrations as rapidly as possible.

2.4 Children

Even if there are no previous data on the use of favipiravir in children, we chose to include children ≥1 year and weighting ≥10kg in the trial.

There are four arguments for this:

First, during the current outbreak, 3.6-6.3% and 7.5-17.4% of patients admitted to the MSF Ebola care centers were <5 and 5-15 year old children, respectively. Of note, mortality was higher in those <5 years of age as compared to older children (MSF, personal communication).

Second, maturation profiles of both the renal function and the enzymes (mainly aldehyde oxydase) involved in the metabolic pathway of favipiravir are fully achieved at the age of 12 months (Tayama Y *et al*. 2007; Tayama Y *et al*. 2012). Below 12 months of, favipiravir disposition is difficult to predict.

Third, EBOV-infected children older than 1 year old could weight less than 10 kg, especially in case of malnutrition and/or dehydration. Because these clinical situations could expose these children to a potential risk of drug overdose, we will only include children weighing ≥10 kg.

Fourth, this is in line with the ICH E11 document on Clinical investigation of medicinal products in the pediatric population (2000), which states that: *"The presence of a serious or life-threatening disease for which the product represents a potentially important advance in therapy suggests the need for relatively urgent and early initiation of pediatric studies. In this case, medicinal product development should begin early in the pediatric population, following assessment of initial safety data and reasonable evidence of potential benefit."*

It is important to state, though, that this trial is primarily powered to conclude on the efficacy of favipiravir in adults. We thought it would be unethical not to include children because we had reasonable reasons to believe that the benefits/risk ratio in children will be favorable (except below 12 months of age) and because we could not envisage to treat adults and not to treat children in the same EVD care centers. We also thought that providing favipiravir to children should be carefully monitored within the framework of a trial, and that a compassionate use was not the best approach. In this trial, even if the power to conclude on favipiravir efficacy in children will be weak, data on favipiravir safety profile will be of utmost importance for its future use in children.

2.5 Influence of baseline viral load on mortality

After the start of the trial two publications (Bah EI *et al*. 2015, Schieffelin JS *et al.* 2014) showed the importance of age and viral load on mortality.

Also, we obtained on January 9 2015, additional information on the pre-trial data of the MSF Macenta EVD Transit Center and the MSF Gueckedou EVD Care Center from Sept 15, 2014 to Dec 14, 2015, that were used to defined mortality boundary. The additional information measured is the EBOV viral load at arrival in the Ebola treatment center, measured by RT-PCR and given as cycle threshold [Ct]. Analysis of these additional data confirmed the very strong link between baseline viral load and mortality, and showed that there is higher mortality in children below 6 years.

Therefore a second definition of group was also used: group AC1: adults and children >6 years with baseline Ct ≥20; group AC2: adults and children >6 years with baseline Ct values <20; group YC: young children ≤6 years and ≥1 year.

2.6 Dose issues

2.6.1 Adults

The recommended dose of favipiravir for influenza in the US is 1800 mg bid on Day-0 followed by 800 mg bid from Day-1 to Day-4. The $IC_{50/90}$ of the drug, as estimated in *in vitro* studies, is higher for EBOV than for H1N1. To propose a dosage regimen in this trial we used the following approach which combined data on favipiravir efficacy against EBOV *in vitro* and *in vivo* with data provided by Toyama Chemical on favipiravir pharmacokinetics in uninfected mice and humans.

First we used the dosage regimen in successfully treated mice to estimate plasma favipiravir concentrations to be targeted in humans. Using data provided by the manufacturer, we determined that 150 mg/kg every twelve hours led in mice to daily minimal (C_{min}, 12 hours post-dosing), average concentrations (C_{ave}, defined by $AUC_{0-24}/24$), half-life ($t_{1/2}$), and maximal concentrations (C_{max}) equal to 5 µg/mL, 58 µg/mL, 1.8 hr and 200 µg/mL on average, respectively. Given that favipiravir plasma protein binding in mice is 10%, unbound average and minimum concentrations C_{min_u} and C_{ave_u} were therefore targeted to 4.5 µg/mL and 52 µg/mL, respectively. Of note the latter is higher than the IC_{99} with Zaire EBOV Mayinga 1976 strain, estimated to 29 µg/mL (Oestereich *et al.* 2014). Plasma protein binding in human being equal to 54%, plasma C_{min} and C_{ave} were therefore targeted to 10 and 113 µg/mL, respectively.

Second we used the pharmacokinetic model developed by the manufacturer in humans with the parameters values estimated in US healthy volunteers to evaluate dosage regimen that could achieve these targeted concentrations. Simulations were performed with various doses of 1000, 1200 and 1800

mg bid and led to median (90% prediction interval) C_{ave} at steady-state equal to 66.8 [57.2, 76.5] µg/mL, 83.3 [72.2, 95.2] µg/mL and 134.4 [115.9, 152.0] µg/mL, respectively. Although the dose of 1200 mg bid gave slightly lower C_{ave} than targeted, it allowed minimizing the chance of relapse (C_{min} of 57 µg/mL) and remained in the range of exposures previously evaluated in humans with good tolerance.

Moreover and given that viral spread has to be blocked as soon and as strongly as possible after appearance of the first symptoms, several loading dose strategies on Day-0 were evaluated to rapidly achieve high levels of exposure. Given the short $t_{1/2}$ of favipiravir, a dose of 2400 mg bid led to a low median C_{min} equal to 4.3 [0.6, 15.6] µg/mL. Rather, concentrations achieved with a regimen of 2400/2400/1200 mg every eight hours allowed to achieve C_{min} at 8 and 16 hours of 9.8 [2.9, 23.5] and 45.2 [12.9, 85.1] µg/mL, respectively.

A dose of 6000 mg on Day-0 (h0: 2400, h8: 2400, h16:1200), followed by 1200 mg bid from Day-1 to Day-9 was therefore found appropriate to reach satisfactory concentration rapidly. Higher dosages were not retained because of the lack of high-dosed related tolerance studies in humans.

Justification of the choice of dosage regimen in adults has been accepted for publication (Mentré *et al.*, 2014).

2.6.2 Children

In children weighing ≥10 kg, the doses will be adapted to the body weight. Using the population model developed by Toyama Chemical, the disposition in pediatric patients aged more than one year is predictable from adults using weight-based allometric scaling. Weight-based favipiravir doses for children were then determined in order to reproduce similar adult drug levels (to reach quick satisfactory concentration and lead to a median state trough concentration of 57 µg/ml) are shown in Table 1.

Table 1. Recommended Weight-based favipiravir doses in children with EVD.

Weight (kg)	Day-0 (mg)			Day-1 to Day-9 (mg)		
	0h	8h	16h	0h	8h	16h
**10 - 15**	500	500	200	200	200	200
	**Day-0 (mg)**			**Day-1 to Day-9 (mg)**		
	**0h**	**8h**	**16h**	**0h**	**12h**	
**16 - 21**	800	800	400	400	400	
**22 - 35**	1200	1200	600	600	600	
**36 - 45**	1600	1600	800	800	800	
**46 - 55**	2000	2000	1000	1000	1000	
**> 55 (adult)**	2400	2400	1200	1200	1200	

Justification of the choice of dosage regimen in children has been accepted for publication (Bouazza N *et al.,* 2015).

2.7 Treatment duration

The duration of the viremic period in patients with EVD is 10 days (Ksiazek *et al.* 1999). In addition, in severely affected patients, profound immunosuppression may occur early after the disease onset, and we do not know whether this would still be the case or not in patients receiving the antiviral. Therefore, we chose to provide favipiravir during 10 days, to ensure optimal probability of success. If the control of viremia occurs early and leads to substantial clinical improvement, shorter duration could be considered in further studies.

2.8 Toxicity issues

2.8.1 Adults

So far, about 2000 subjects received at least one dose of favipiravir during the clinical development of the drug for the treatment of influenza. Among them, several hundred received a loading dose of 3600 mg (1800 mg bid or 2400 mg, 600 mg, 600 mg administered on an 8h schedule). Side effects were rare, and included diarrhea (2.8%), asymptomatic hyperuricemia (1.5%), asymptomatic increase in liver enzyme (AST, ALT, gamma-GT) (1.3%), asymptomatic decrease in neutrophil counts and white blood cells, headache, asymptomatic increase in triglycerides (1%), vomiting, dizziness and proteinuria (0.9% each). Details from the investigator's brochure are available in Appendix 6.

Based on non clinical data, three additional issues have been raised:

- QT prolongation: positive findings were shown on the hERG channels. Consequently a dedicated QT/QTc study was performed in healthy adult volunteers with a single dose of 2400 mg. Although this study was negative, the potential risk of QT prolongation cannot be strictly ruled out in the context of EVD (higher doses, diarrhea with dyskaliemia).
- Pregnancy: early embryonic deaths and teratogenicity were reported in animal studies (Baranovich *et al*. 2013). In consequence, favipiravir is not recommended in women known to be pregnant, and it is recommended that women of child bearing potential be tested for pregnancy before receiving the drug. Because of this, the sponsor could not find any insurance company keen to insure pregnant women participating in the trial. However, in the largest case series published (Mupata *et al*. 1999) the mortality in EVD-infected pregnant women was 93% (14 out of 15 pregnant patients died in an outbreak in DRC). The one patient who survived delivered a preterm dead fetus. The perinatal mortality in this publication was 100%. One infant was born alive but died after three days (Mupata *et al.* 1999). Johnson *et al.* also in DRC in 1978 reported a mortality rate in pregnant women of 89 % (73 out of 82 pregnant women with EVD died) and there were no survivors among the neonates. Up till now no reports have been published of neonates born to mothers with EVD surviving more than 3 days (Jamieson *et al.* 2014). Given these outcomes of EVD in pregnant women and MSF unpublished data from the current epidemic (mother death, or spontaneous abortion if the mother survives), the benefit/risk ratio of receiving the drug has been deemed to be likely positive for both the mother and the baby. Thus, emergency use of favipiravir in pregnant women outside of the trial is envisaged and under evaluation.
- Fertility: favipiravir passes into the sperm. Testicular findings were reported in animal, which led to perform a testicular study in human (1200 mg bid on Day-1, followed by 800 mg bid for 4 Days). The results did not show significant impairment on seminal parameters.

2.8.2 Children

So far no children have been exposed to favipiravir. Juvenile toxicity animal’s studies have shown higher level of toxicity with lower exposure as compared to adult animals. However, it is difficult to extrapolate these findings to children.

Below 12 months of age and below 10 kg, drug disposition is difficult to predict for favipiravir. Therefore, we will not include infants below 12 months or below 10 kg in order to avoid inaccurate prediction of drug levels and limit the potential risk of drug overdose.

2.9 Context implications

The investigators of the present trial subscribe to the MSF principle that a research program should not have negative impact on patients care and need to be time/context adapted from start to end.

This trial takes place in a very unusual context:

- A highly contagious fatal disease where every additional medical gesture entails additional risks to health care staff;

- A huge complexity to organize effective prevention and care with multiple factors and conditions of care that may evolve within time according to programmatic decisions;
- A volatile emergency epidemic situation, in which the number of cases in a given center can change very fast;
- An international community mobilization that could lead knowledge to evolve rapidly, a situation to which researchers should adapt in real-time.

This context influences the trial in the following ways:

- The trial has to fit in a simplest possible way into the conditions organized by the care givers and organizers (Ministry of health, NGO, other institutions). In particular:
    - additional body fluid collection and sample manipulations prior to cryoconservation, should be limited to the bare minimum;
    - data collection should be simple, with no circulation of material (such as CRFs) from the inside towards the outside of the centers;
    - statistical power considerations impose to conduct the main analysis on data from the group of patients in whom the treatment benefit is expected to be the greatest; however, any individual with EVD and no contraindication to favipiravir who is taken care of in the same trial center should have access to the drug if s/he may potentially benefit from it, even if s/he does not belong to the group in whom the treatment benefit is expected to be the greatest;
    - once favipiravir has been added to the current care, any other decisions regarding modification of the current care practices should be taken with the care actors, and only if they are considered necessary to improve care (not for the very sake of the trial).
- Particular attention should be paid to the risks for trial staff and health care personal. This imposes to eventually consider post-exposure prophylaxis with favipiravir, even in the absence of previous evidence of its efficacy, and to have a clear procedure for high standard of care in the event of an exposed personal becoming infected with EBOV. The best known post-exposure prophylaxis regimen is under evaluation.

3. Objectives

Primary objective: to assess the efficacy of high-dosed favipiravir in reducing mortality in humans with EVD

Secondary objectives: to assess the efficacy of high-dosed favipiravir in reducing mortality in humans with EVD according to age and baseline viral load; to assess the evolution of EBOV plasma RNA and infectious loads under treatment; the tolerance of favipiravir; the viral micro-diversity of EBOV; the trough concentrations of favipiravir; and factors associated with mortality and toxicity.

4. Study design

Non-comparative, proof-of-concept, phase II trial.

5. Study sites

The trial will take place in several EVD care centers in Guinea.
It will start in December 2014 in the MSF EVD care center in Gueckedou.
Depending of the inclusion rate and on the evolution of the outbreak, other EVD care centers will join in and become trial centers in January 2015: the French Red Cross EVD care centre in Macenta, the ALIMA EVD care centre in Nzerekore, the caregivers treatment center (STC) managed by the health services of the French aries, the MSF EVD care center in Donka, the MSF EVD care center in Cancan, and the EVD care center in Coyah.

6. Participants

6.1 Inclusion and non-inclusion criteria

Inclusion criteria: age ≥1 year and weighting ≥10kg, EVD confirmed by a positive qualitative PCR test, signed informed consent (signed by the parents/adults guardians in case of minor patient).

Non inclusion-criteria: pregnancy*, inability to take the drug (encephalopathy, severe vomiting). (**for pregnant women, see sections 2.8*).

6.2 Groups

In this protocol, we will refer to the following groups according to age and duration of symptoms*: Group A1: adults with time between first symptoms and first dose of favipiravir ≤72h; Group A2: adults with time between first symptoms and first dose of favipiravir >72h and Group C: all children ≥1 year and weighting ≥10kg. Time of first symptom refers to the time of the beginning of any symptom considered to be related to EVD.

* Symptoms to be considered will be: acute onset of fever, severe headache, myalgia, extreme fatigue, vomiting, diarrhoea, abdominal pain, or unexplained hemorrhage.

The division in groups is a matter of analysis, and will not be perceptible by the patients during the trial process. Patients in the three groups will receive the same treatment and will be followed under the same procedures. Daily dosages will be adapted to the body weight in group C.

A second definition of group was also used: group AC1: adults and children >6 years with baseline Ct ≥20; group AC2: adults and children >6 years with baseline Ct values <20; group YC: young children ≤6 years and ≥1 year.

6.3 Sample size and power

Because we expect the efficacy of the treatment to be the maximum in patients with early symptoms, the sample size calculation was based on the analysis of mortality in group A1.

The observed pre-trial mortality was estimated using the three months pre-trial data of the MSF Macenta EVD Transit Center and the MSF Gueckedou EVD Care Center from Sept 15, 2014 to Dec 14, 2015. Given those observations, and to remain pragmatic and conservative, we set the pre-trial mortality to 55% for groups A1, A2, C, and to 30%, 85%, 70% for groups AC1, AC2, YC, respectively.

With 60 participants in group A1, the power to conclude that mortality in the trial will be -20% inferior to pre-trial mortality will be equal to 89%.

Recruitment will be kept opened in all groups until 60 patients are recruited in Group A1, unless otherwise recommended by the DSMB (*see stopping rules in section 9.3*). Once 60 patients are included in Group A1, recruitment will stop in all groups, irrespective of the number of patients included in other groups. When recruitment stops, new patients who show up next will either receive favipiravir or another treatment within the framework of the next phase of the research (*see section 10*). There will be no gap between the present trial and the next phase of the research.

We estimate that 80% of adults with suspected EVD and symptoms will be confirmed to have EVD with a positive PCR. Thus, 75 patients will be eligible to group A1 (including 15 with negative PCR who will not be included, and 60 with positive PCR who will be included in analysis). The number of patients in groups A2 and C will depend on the proportion of adults showing up with ≤72h symptoms and on the proportion of children in the population of patients with EVD referred to the three trial settings during the study period.

7. Outcomes

Primary outcome: mortality by Day-14. Day-0 is the day of the first dose of favipiravir.
Secondary outcomes:

- Mortality by Day-14 according to the second group definition (AC1, AC2, YC).

All the following outcomes will be analyzed overall and separately by the two group definitions (A1, A2, C or AC1, AC2, YC):

- Evolution of EBOV plasma RNA and infectious loads (sampling time depending of the group);
- Occurrence of grade 3 or 4 clinical or biological adverse events (Common Terminology Criteria for Adverse Events, CTAE, v3.0);
- Evolution of viral micro-diversity of EBOV (including potential resistance mutations);
- Plasma trough concentrations of favipiravir (sampling time depending of the group);
- Criteria for cure at Day-30 (end of follow-up visit);
- Factors associated with mortality and toxicity (including time from first symptoms to treatment initiation; evolution of EBOV RNA and infectious loads after treatment initiation and trough concentrations of favipiravir).

8. Trial conduct

8.1 Sponsor and coordination

The trial sponsor is the INSERM. The international coordinating, monitoring and data management centre will be the Mereva clinical trial unit (CTU), an international team with members affiliated to the Inserm 897 Unit, University of Bordeaux and to the Pacci/ANRS research site in Abidjan. Trial coordinating, monitoring and data management activities will be coordinated by an International Clinical Project Manager (CPM). In Guinea, where the trial will be conducted, a trial country coordination center (CCC) will be put in place, lead by a country CPM who will work in close collaboration with the international CPM and with the participating clinical centres.

The trial will be conducted and monitored according to a Standard Operating Procedures (SOPs) manual.

8.2 Trial drug

Favipiravir tablets are light yellow, round-shaped, coated divisible tablets containing contain 200 mg of favipiravir drug substance. They can be crushed and mixed with liquid, provided this is done just before the administration.

Favipiravir is rapidly and completely absorbed after oral administration. The major metabolite is inactive and excreted predominately in the urine. Food does not appear to alter the absorption of the current tablet formulation.

Drug-drug interactions were identified with pyrazinamide, repaglinide, theophylline, and sulindac. None of these drugs are part of the standard package of care currently used in EVD care centres.

The investigator's brochure is attached to the protocol.

8.3 Trial development and scheduled assessment

Once the trial is opened, all patients who attend the trial centers will be assessed for inclusion and non-inclusion criteria. Those who fulfill inclusion criteria will be informed and proposed to participate. Those who accept will be asked for informed consent.

- **Trial treatment**:
  - Adults (groups A1 and A2): Day-0, H0: 2400 mg (12 tablets); H8: 2400 mg (12 tablets); H16: 1200 mg (6 tablets). Day-1 to Day-9: 1200 mg (6 tablets) bid.
  - Children (group C): The loading dose (Day 0) and the dose from Day-1 to Day-9 will be adapted to the body weight (*see Table 1*).

- **Other treatments**: From Day-0 to end of follow-up, all participants will receive the standard package of care, as defined by the care providers. This will be the object of a data collection, but will not be imposed by the protocol.
- **Blood collection** (*see Tables 2 and 3*):
  - Adults (groups A1 and A2): blood will be collected in 4 ml trial tubes during: (i) routine care venepuncture (Day-0, end of symptoms (EOS)+72h); (ii) additional trial venepuncture at: Day-2, Day-4, Day-13 and Day-30.
  - Children (group C): blood will be collected in 4 ml trial tubes during: (i) routine care venepuncture (Day-0, EOS+72h); (ii) additional trial venepuncture at Day-2, Day-4, Day-13 and Day-30.

Table 2: assessment schedule in adults

	Clinical examination	Favipiravir (mg)	Blood collection	Additional venepuncture**	Additional laboratory manipulation ***
Day-0	x	6000 ‡	1 RCT + 1 TT *	No	Yes
Day-1	x	1200 x 2	No	No	No
Day-2	x	1200 x 2	1 TT *	Yes	Yes
Day-3	x	1200 x 2	No	No	No
Day-4	x	1200 x 2	1 TT *	Yes	Yes
Day-5 to Day-9	x	1200 x 2	No	No	No
Day-10 to discharge	x	No	No	No	No
EOS+72h	x	No	1 RCT + 1 TT *	No	Yes
Day-13	x	No	1 RCT	No	No
Day-30	x	No	1 TT *	Yes	Yes

EOS: end of symptoms; ‡ H0: 2400 mg; H8: 2400 mg; H16: 1200 mg;
** RCT: routine care tube; TT: trial tube (4 ml EDTA tubes); ** Venepuncture not included in routine care;*
**** Tube centrifugation and cryoconservation.*

Table 3: assessment schedule in children

	Clinical examination	Favipiravir	Blood collection	Additional venepuncture**	Additional laboratory manipulation ***
Day-0	x	Yes ‡	1 RCT + 1 TT *	No	Yes
Day-1	x	Yes ‡	No	No	No
Day-2	x	Yes ‡	1 TT *	Yes	Yes
Day-3	x	Yes ‡	No	No	No
Day-4	x	Yes ‡	1 TT *	Yes	Yes
Day-5 to Day-9	x	Yes ‡	No	No	No
Day-10 to discharge	x	No	No	No	No
EOS+72h, or Day-12	x	No	1 RCT + 1 TT *	No	Yes
EOS+96h, or Day-13	x	No	1 RCT *	No	No
Day-30	x	No	1 TT *	Yes	Yes

EOS: end of symptoms; ‡ Dosages adapted to the body weight (see Table 1);
** RCT: routine care tube; TT: trial tube (4 ml EDTA tubes);** Venepuncture not included in routine care;*
**** Tube centrifugation and cryoconservation.*

According to the European ethical recommendations, the trial-related blood loss will not exceed 3% of the total blood volume overall (2.4 ml blood per kg body weight) and will not exceed 1% at any single time.

- **Hospital stay:**

  All patients will be hospitalized in the isolation ward of the EVD care center until Day-14, including patients who reach criteria for cure before Day 14. Patients who meet criteria for cure before Day 14 or at Day 14 will be discharged at Day 14. Other patients will be hospitalized until criteria for cure are met.

  Criteria for cure are the following:
  - 4 days without fever or significant symptoms AND;
  - able to feed and walk independently AND;
  - two consecutive negative qualitative PCR.

- **End-of-follow-up visit**:

  Patients discharged before Day-30 will be asked to show up at the trial center at Day-30 to undergo the end-of-follow-up visit. The visit will consist of a clinical examination and a blood sample collection. In the event of a patient being symptomatic at this visit, examination-guided tests will be performed and appropriate treatments will be given. If patients fail to report at Day-30, they will be contacted by phone or visited at home.

8.4 Biology

In trial settings laboratories:

- At Day-0, Day-2 Day-4, Day-13 the following tests will be done: qualitative PCR (Altona qualitative RT-PCR commercial kit); and biochemical tests (creatininemia and others tests according to local choice), if biochemical tests are doable in the laboratory.

- At Day-30 the following tests will be done: biochemical tests (creatininemia and others tests according to local choice) only if tests at Day-13 were abnormal.

- EDTA tubes will be decanted, and the serum will be divided into two aliquots of up to 1 ml. Aliquots will be frozen at -20 °C.

Outside trial settings laboratories: all trial samples fill be centrifuged and frozen on the day of collection. They will be used for virological tests that are currently not available in Guinea and that need to be done in a Biosafety level 4 laboratory. Therefore, they will be shipped frozen to the Lyon-Mérieux BSL4 laboratory, using international standard procedures, including triple-layer packaging (collection tube, plastic waterproof boxes, special cartoon box with the international label for transport of Infectious Substances: UN6.2 class A, UN2814), and disinfection procedures for the outside of the tube and inside/outside of the box and of the cartoon. A Material Transfer Agreement will be obtained by the sponsor.

In the BSL4 laboratory, Quantitative RT-PCR will be performed using a standard real-time RT-PCR procedure and a set of synthetic RNA quantified external calibrators. The infectious load will be evaluated by determining the number of infectious viral particles using a standard procedure relying on serial dilutions of sera and infection of Vero cells. Evolution studies will rely on the analysis of the kinetics of viral subpopulations as determined using next-generation sequencing methods. In brief, a series of overlapping PCR products spanning the complete genome will be generated using a high fidelity amplification protocol and sequenced using the Ion Torrent NGS platform. After quality check and trimming primer sequences, complete genome sequences will be rebuilt and used for sub-population analysis.

The following tests will also be made in the Lyon-Mérieux BSL4 laboratory:

- Concentrations measurement will be made by HPLC using methods provided by the manufacturer of the drug.
- Uricemia
- Other biochemical tests: liver enzymes, creatininemia, natremia and kaliemia (expect if the tests have been made in the setting laboratory).

These tests will be completed within the six months following collection. Any remaining material will be stored in Lyon-Mérieux BSL4 laboratory for 10 years. Any future use will be approved by the committees that gave approval to the initial study. These samples will only be used in the framework of non-commercial research.

8.5 Trial management

8.5.1 Monitoring

The Mereva CTU and CCC will coordinate all study activities of the Project Team. This includes in particular to:

- Set up and monitor workflows, timelines, milestones and tracking tools;
- Conduct regular team meetings to discuss project status, activities, and address issues;
- Maintain daily contact with principal investigators, sponsor and clinical sites;
- Provide status reports to principal investigator, DSMB, and SAB;
- Support the study sites with study related questions;
- Ensure that study drug is being stored, dispensed, and accounted for according to specifications;
- Check the completeness of patient records, the accuracy of entries in the database, the adherence to the protocol, SOPs and to Good Clinical Practice. Monitoring standards require full verification for the presence of informed consent, adherence to the inclusion/exclusion criteria, documentation of serious adverse events (SAEs), and the recording of data that will be used for all primary and safety variables.

The investigator must give the CCC access to all relevant source documents to confirm their consistency with the CRF and database entries. For this particular trial, some documents filed in within the isolation ward will have to be destroyed in order to avoid contamination. These documents will be scanned or photographed prior to destruction.

Investigators at each clinical site will keep a hard copy of the Trial Master File (TMF), containing all essential technical (protocol, SOPs, etc.), and regulatory (insurance, IRB approvals, task delegation descriptions, bio-sketches, agreements, etc.) trial documents. The country CPM will be responsible for checking that the files containing the hard copy of the TMF documents at each trial clinic and at the CCC are up-to-date. All TMF documents will also be made available online to investigators on a private website ("eTMF"). The international CPM will be responsible for routinely updating the documentation on the trial website.

8.5.2 Data management

The Mereva CTU will develop by December 1st, 2014:

A case report form (CRF): data will be collected on hard copy CRF in the EVD center. It will then be scanned or photographed prior to destruction. The medical investigator or his/her designated representative will be responsible for filling out the forms.

A database: data will be entered online in a system that will ensure data encryption, restricted access to the database, daily back-up, and tracking of edits.

A Data Management Plan (DMP) and Data Validation Plan (DVP), including the quality control process to ensure completeness, validity, consistency, timeliness and accuracy.

Training will be provided to all members of the study team involved in data collection data entry, data check and monitoring.

The Mereva CTU data managing team will conduct data review and handle queries on a weekly basis, and reconcile the safety database with the clinical database.

8.5.3 Pharmacovigilance and safety management

Due to the international standard procedures for the prevention of EBOV transmission, body fluid collection will be limited to the bare minimum and tests such as X-ray or electrocardiogram will not be doable.

The following events will be defined as SAE, provided they occur after the first drug administration:

- death or life-threatening events,
- prolonged hospitalization, defined of a patients aggravation following improvement,
- grade 4 CTAE adverse events,
- grade 3 CTAE adverse event of special interest (rash, jaundice, clinical suspicion of anemia, petechiae, external bleeding, bradycardia, tachycardia, arrhythmia),
- and any events considered as medically important by the investigator.

Deaths will be reported to the sponsor and to the DSMB on a daily basis; SAE other than deaths will be reported to the sponsor and to the DSMB on a weekly basis. In addition, the DSMB will receive the results of all biological tests as soon as they are available.

8.6 Study medication manufacturing, packaging and shipping

The Investigational Medicinal Product (favipiravir) will be manufactured according to GMP requirements by Toyama, Inc. Quality control testing has been performed to confirm that the study medication complies with all the defined quality standards for human use. Manufacturing records and a Certificate of Analysis will be provided to the trial investigators.

The study medication will be packaged in aluminum pouches including 240 tablets (24 tablets x 10).

A defined quantity of study medication will be shipped to the Japanese Embassy in Guinea in appropriate conditions. Relative humidity and temperature will be controlled during shipment.

Drugs will be picked up at the Embassy by a MSF team and transported to the trial central pharmacy in Gueckedou, where it will be kept in a safe place at controlled room temperature <30° C.

At the end of the trial, the unused and/or returned investigational medicinal products will remain at the disposal of the Guinean health authorities, who will be authorized to use it according to national procedures.

8.7 Trial surveillance

8.7.1 Safety monitoring board

An independent Data Safety Monitoring Board (DSMB) of seven external experts will oversee the trial, with special attention to severe adverse events, mortality, and number of subjects needed. The DSMB members are pharmacologist, statistician, virologist, anthropologist, pediatrician, infectious diseases specialist and experienced lead investigator in clinical trials in developing countries (*see the list in Appendix 5*). They will help make decisions that require an independent assessment while the trial is underway. This includes:

- Premature discontinuation of the inclusion in group A1, A2 and/or C, because of futility or toxicity criteria are met (*see section 10*), or because external new fact lead to recommend such discontinuation;
- Other substantial changes to the protocol deemed to be necessary based on the trial data or on external new facts.

Intermediate analysis for futility will be prepared by the steering committee and interpreted by the DSMB.

The recommendations from the DSMB with all documentation sent from the sponsor to the DSMB will be circulated to three ethics committees after each session.

8.7.2 Steering Committee and Scientific Advisory Board

The Steering Committee is composed of leading investigators in each field. They meet on a weekly basis (*see the list in Appendix 5*).

The trial Scientific Advisory Board (SAB) is composed of the members of the steering committee, external experts and representatives of the sponsor. The SAB meets before the beginning of the inclusion and then at least once a month until the end of the trial. The SAB guarantees that the trial remains scientifically relevant and that the methods used are appropriate, in light of the reports made by the investigators and of any external new facts. It decides about protocol amendments, and whether to open or close trial sites (*see the list in Appendix 5*).

9. Statistical analysis

9.1 Mortality

Mortality at Day-14 will be reported with its exact 95% confidence intervals (CI), overall, and separately by groups (A1, A2, C).

The analyses will be performed first using the intention to treat approach, including all patients with positive PCR who received at least one dose of favipiravir. In case of missing data for mortality at Day-14 patient will be considered dead.

A per protocol analysis will then be performed, including all patients who received the treatment until Day-9 or death.

In each group, we will conclude that favipiravir decreases mortality if the upper bound of the 95% CI does not include the observed pre-trial mortality in untreated patients with same duration of symptoms prior to trial initiation (*see section 6.3*).

In group A1, if the number of deaths is <24 (40%), <19 (32%) <13 (22%) or <8 (13%) out of 60 participants, the upper bound of the 95%CI will be <55%, <45%, <35% and <25%, respectively.

In group A2, if the number of deaths is <54 (45%), <42 (35%), <31 (26%), <20 (18%) out of 120 participants, the upper bound of the 95%CI will be <55%, <45%, <35% and <25%, respectively.

In group C, if the number of deaths is <17 (38%), <13 (29%), <9 (20%), <5 (11%) out of 45 participants, the upper bound of the 95%CI will be <55%, <45%, <35% and <25%, respectively.

9.2 Other outcomes

Mortality by Day-14 with exact 95%CI will be reported according to the second group definition (AC1, AC2, YC). In each group, we will conclude that favipiravir decreases mortality if the upper bound of the 95% CI does not include the observed pre-trial mortality (30%, 85% and 70%, respectively) in untreated patients with same characteristics of symptoms prior to trial initiation (*see section 6.3*).

All the following outcomes will be reported overall and separately by the two group definitions (A1, A2, C or AC1, AC2, YC).

The evolution of EBOV plasma RNA and infectious loads between Day-0 and the end of follow up will be described in each patient. The numbers, proportions and exact 95% CI will be described for grade 3-4 adverse events; resistance mutations; and patients reaching criteria for cure at Day-30. The distribution (median, IQR, min-max) of initial and maximal viral load, time to maximal viral load and to undectability, and rate of increase/decrease will be reported. The distribution of trough concentrations of favipiravir at each point and the inter- and intra-patient variability of concentrations will be described.

Factors associated with mortality by Day-14, cure at Day-30 and grade 3-4 clinical or biological adverse events (including time between first symptoms and treatment initiation, evolution of EBOV viral load, trough concentrations of favipiravir) will be studied.

9.3 Intermediate analyses and stopping rules for futility

Futility: In group A1, two intermediate analyses of mortality are planned after the 20th and 40th definitively included patients reaches Day-14 (or die prior to Day-14), respectively. Recruitment in group A1 will be stopped if the results show that the trial is unlikely to prove that mortality with favipiravir is <55%. The DSMB will be in position to recommend to stop inclusion in group A1 for futility if the number of deaths is ≥14 among first 20 patients, and ≥22 among first 40 patients in group A1.

In group A2, five intermediate analyses of mortality are planned after 20, 40, 60, 80 and 100 definitively included patients reach Day-14 (or died prior to Day-14), respectively. Recruitment in group A2 will be stopped if the results show that the trial is unlikely to prove that mortality with favipiravir is <55%. The DSMB will be in position to recommend stopping inclusion in group A2 for futility if the number of deaths is ≥14, 22, 29, 36 and 44 among first 20, 40, 60, 80, and 100 patients, respectively.

In group C, four intermediate analyses of mortality are planned after the 10, 20, 30 and 40 definitively included patients reaches Day-14 (or die prior to Day-14), respectively. Recruitment of children will be stopped if the results show that the trial is unlikely to prove that mortality with favipiravir is <55%. The DSMB will be in position to recommend stopping inclusion in group C for futility if the number of deaths is ≥10, 14, 18 and 22 among first 10, 20, 30 and 40 patients, respectively.

Toxicity: The Mereva CTU (*see section 8*) will make available to the DSMB members a day-by-day-standardized report of the number of patients included, dead, and alive at end of follow-up. SAE other than death will be reported to the DSMB on a weekly basis. In addition, the DSMB will receive the results of all biological tests available at each intermediate analysis. The DSMB may decide to carry out an intermediate analysis if the number of observed deaths or SAEs is higher than anticipated.

10. Trial termination

If the DSMB does not recommend early termination, recruitment in all groups will stop as soon as 60 patients are included in group A1. The trial will stop when all participants reach end of follow-up, *i.e.*: Day-30 or death, if the patients dies before Day-30.

Because of the sensitiveness of the data in the context of a deadly disease outbreak, the Scientific Advisory Board will immediately review exhaustive mortality data and partially available biological data, in order to conclude and provide the Guinean health authorities with recommendations regarding the use of favipiravir in immediate care and/or in further research steps. If data suggest that favipiravir provides a significant benefit to the patients, it will be made available to new patients with EVD.

This trial is the first phase of a research aiming at finding the best antiviral treatment for patients with EVD. Depending on the results of this first phase, further phases could consist of continuing favipiravir alone, replacing favipiravir with brincidofovir, combining both drugs, combining one of these drugs with other interventions, or any other options that would arise in the interval based on new data and on drug availability.

11. Ethical and regulatory issues

11.1 Communication with regulatory authorities and ethics committees

Ethics:
Prior to commencement of the study, the investigators will seek approval from:

- The Inserm IRB00003888/FWA00005831;
- The Comité National d'Éthique pour la Recherché en Santé (Guinea);
- The ethics committee of MSF.

Safety:
- The protocol of the trial as well as the IMP documentation has been has been discussed with the French Drug Agency (ANSM: Agence Nationale de Sécurité du Médicament et des Produits de Santé).

11.2 Informed consent

The availability of an informed consent prior to the study is a criterion for study participation. The investigators will ensure that the information provided to the patients is clear, using the language deemed the most appropriate, and easy to understand. In case of minor patients, both children and their parents/guardians will be informed and the availability of written informed consent, signed by the parents/adult guardians will be required prior to inclusion.

The informed consent and information sheets for adults and children are shown in **Erreur ! Source du renvoi introuvable.**. Consent will normally be given by signing the 'informed consent form'. If the consent cannot be given in writing, fingerprint consent or any other form of non-written consent will be formally documented. Witnesses (all of them members of the staff of the ETC, because external people are not allowed to enter the high risk zone) may participate in the process, under the condition that this is accepted by the patient.

11.3 Safety

Given the high risk of transmission of EBOV to health care personal, this study raises safety issues. The sponsor and the study team will pay particular attention to the following:

- All research personal participating in the study will be informed of the nature of the research, including the risks for themselves, the necessity to follow strict prevention procedures, and the procedure to be followed in the event of an accidental exposure (*see* **Erreur ! Source du renvoi introuvable.**);
- All research and care personal participating in the study will be trained to international standard procedures for the prevention of EBOV transmission, and be equipped with the international standard material before getting in contact with patients;
- A post exposition prophylaxis (PEP) procedure will be written in collaboration between the care actors and the trial SAB. It may include early treatment with favipiravir and will be updated in real time according to the most recent evidence;
- The sponsor, in close collaboration with the French Ministry of Foreign Affairs, will ensure that all research and/or care personal participating in the study receive the highest standard care in case they get infected with Ebola during the study period. This includes medical insurance plans to any personal from abroad travelling to Guinea as well as to the Guinean personal recruited for the study.

11.4 Social aid

The study budget includes social aid. Social workers in charge in each clinical center will be free to use it to help patients prepare their return home after they are declared cured, or help families in case of patient's death.

11.5 Partnership

The staff on the investigational site and the partners head quarter members involved in activities related to the trial will be member of the study group, and, as such, will be proposed to co-author publications. International collaborative clinical research has two complementary goals: improving care and patients outcomes in the short terms; and helping partners develop clinical research skills and facilities in the medium term. The Inserm 897 team has been working in partnership with African

scientific partners for the past twenty years, and half of their staff is African. In the present study, the Dermatology and Infectious Diseases departments of the Donka Hospital, and the Centre de Formation et de Recherche en Santé Rurale will actively participate in the study coordination, as well as in national and international Ebola networks. Five Guinean professionals who participate in the trial teams will receive a master scholarship for the year 2015-2016 to be trained in clinical research (n=2) and in anthropology (n=3).

All institutions listed in appendix 5 as having a representative in the steering committee are partners of the trial consortium. This includes the Ebola task force in Guinea, several Inserm units (which are seperate entities, different from the Inserm national team who acts as a sponsor), Médecins Sans Frontière, French Red Cross, ALIMA, Pasteur Institute, EMLAB, Centre de Formation et de Recherche en Santé Rurale, Université Cheikh Anta Diop Universiteit van Amsterdam, Ecole Nationale Supérieure de Lyon, association Solthis, and the Treichville, Donka, Necher, Bicètre, and Bordeaux hospitals. All steering committee members are also investigators of the trial and members of the SAB. As such, they have equal partnership in terms of scientific participation (inputs, decisions, interpretations, initiative…), under the scientific leadership of the two principal investigators.
In terms of publications, all investigators will be members of the trial study group, and all publications will be made "in the name of" the study group (all investigators names are mentioned) or "with" the entire study group (all investigators are co-authors), depending on the subject of the publication (the main results will be published, partial data may be published "in the name of", and written by those investigators who have been directly involved in specific aspects.

11.6 Community involvement

Clinical trials must be acceptable to all parties. Their implementation requires trust between medical and target communities in addition to specific provisions relating to the public understanding of science and the interventions being studied. In Guinea as elsewhere, popular reactions to the Ebola epidemic in general and the response in particular have been fuelled by distrust, rumours articulating conspiracy theories, even triggering sporadic violence. Such phenomena are not irrational, but have a historical and political basis in the history of the region, the relationship to the State, and experience of past public health interventions back to colonial times that were at times coercive.

Our strategy to foster ownership of the trial will be:

1) Target key community leaders and give them the information enabling them to answer question and speak on behalf of the clinical trial. This was already done in the first trial site, Gueckedou, in mid-November, and will be done in other sites in early December. In Gueckedou, the list of community leaders includes:

- Civil servant in charge of the administration of the Prefecture de Gueckedou
- General director of Health for Guinée forestière
- Ebola coordinator at the prefecture level
- Ebola focal point at the level of the sous-prefecture
- Council of elders of Gueckedou Region
- Council of the youngster of Gueckedou
- President of APAGE (Association des personnes affectées et guéries d'Ebola/ Association for affected and cured people from Ebola)
- Imam of Gueckedou
- Priest of Gueckedou

2) To foster the articulation of practice and communication

The spread of rumours and of situation of misunderstanding is highly related to violent actions (isolation of patient, protected burial, spray of housing) carried out without the necessary explanations nor the understandings of the legitimate populations fears. We will pay particular attention to communicate with patient families with the help of a team of local psychologist and health promoters:

- At the arrival of patients

- At their departure
- At their death with their family
- When doing outreach programs

This will be done using cell phones or during face to face interactions. A follow up of patient situation when back home will be organizing by phone. In case of need, a team will meet the patient at home.

3) To foster the ownership of the project by the workers of the EVD care centers (from lay workers to medical personnel). Being at the heart of the trial, they will be the first to be exposed to questions from the communities in which they belong. Being clear to them about the clinical trial and fostering their appropriation of the project is the best way to turn them into positive communicators.

11.7 Communication of results

Three kinds of results will be communicated. The first involves « raw » findings from ongoing research, and these will be communicated and discussed internally first with staff and possibly selected community representatives. This first level of discussion contributes to the participatory nature of the analysis of results as noted above. The second involves specific messages to be communicated by clinical trial staff to patients and the broader community. These messages will be elaborated through the consultative process outlined above and great care will be made to liaise with other actors in the field to ensure that messages are simple, consistent, and are unlikely to conflict with future messages (for instance if a drug is found to be ineffective). Finally, longer term results evaluating strategy and drawing lessons will be communicated through the usual academic mechanisms (peer reviewed articles, participation in workshops and conferences, etc.).

We expect the benefits to be greater public understanding of the research, receptivity to research results, and better knowledge to inform consent by participants. Potential risks to participants are few, and are linked to the stigma associated with the disease. The ethnographic approach that involves participation in everyday life reduces these risks considerably.

12. Timeline

This is a project within an exceptional context of emergency, with the following timeline:

- Sept 30, 2014: first version of the protocol;
- Oct 1- Nov 31, 2014: finalization of the protocol, process of ethical approval; development of the eTMF, SOPs and database; recruitment and training of the team; supply in medicine;
- Dec 15, 2014: first inclusion.
- May 31, 2015: last inclusion.
- June 30, 2015: end of the trial.
- August 15, 2015: final results for the clinical criteria (tolerance and mortality).
- September 30, 2015: final results for the virologic criteria (quantitative PCR).

13. References

Bah EI, Lamah MC, Fletcher T, Jacob ST, Brett-Major DM, Sall AA, Shindo N, Fischer WA 2nd, Lamontagne F, Saliou SM, Bausch DG, Moumié B, Jagatic T, Sprecher A, Lawler JV, Mayet T, Jacquerioz FA, Méndez Baggi MF, Vallenas C, Clement C, Mardel S, Faye O, Faye O, Soropogui B, Magassouba N, Koivogui L, Pinto R, Fowler RA. Clinical presentation of patients with Ebola virus disease in Conakry, Guinea. *N Engl J Med*. 2015;372(1):40-7.

Baize S, Pannetier D, Oestereich L, et al. Emergence of Zaire ebola virus disease in Guinea. *N Engl J Med.* 2014;371(15):1418-25.

Baranovich T, Wong SS, Armstrong J, Marjuki H, Webby RJ, Webster RG, Govorkova EA. T-705 (favipiravir) induces lethal mutagenesis in influenza A H1N1 viruses in vitro. *J Virol*. 2013;87(7):3741-51.

Bouazza N, Treluyer JM, Foissac F, Mentré F, Taburet AM, Guedj J, Anglaret X, de Lambalerie X, Keita S, Malvy D, Frange P. Favipiravir for children with Ebola. *Lancet.* 2015;385(9968):603-4.

Feldmann H1, Geisbert TW. Ebola haemorrhagic fever. *Lancet*. 2011;377(9768):849-62.

Fowler RA, Fletcher T, Fischer WA, Lamontagne F, Jacob S, Brett-Major D, Lawler JV, Jacquerioz FA, Houlihan C, O'Dempsey T, Ferri M, Adachi T, Lamah MC, Bah EI, Mayet T, Schieffelin J, McLellan SL, Senga M, Kato Y, Clement C, Mardel S, Vallenas Bejar De Villar RC, Shindo N, Bausch D. Caring for critically ill patients with ebola virus disease. Perspectives from West Africa. *Am J Respir Crit Care Med*. 2014;190(7):733-7.

Furuta Y, Takahashi K, Shiraki K, Sakamoto K, Smee DF, Barnard DL, Gowen BB, Julander JG, Morrey JD. T-705 (favipiravir) and related compounds: Novel broad-spectrum inhibitors of RNA viral infections. *Antiviral Res*. 2009;82:95-102.

Furuta Y, Gowen BB, Takahashi K, Shiraki K, Smee DF, Barnard DL. Favipiravir (T-705), a novel viral RNA polymerase inhibitor. *Antiviral Res*. 2013;100:446-54.

Furuta Y, Takahashi K, Fukuda Y, Kuno M, Kamiyama T, Kozaki K, Nomura N, Egawa H, Minami S, Watanabe Y, Narita H, Shiraki K. In Vitro and In Vivo Activities of Anti-Influenza Virus Compound T-705. *Antimicrob Agents Chemother.* 2014;46: 977–981.

Gowen BB, Wong MH, Jung KH, Sanders AB, Mendenhall M, Bailey KW, Furuta Y. Sidwell, R.W. In vitro and in vivo activities of T-705 against arenavirus and bunyavirus infections. *Antimicrob Agents Chemother*. 2007, 51, 3168–3176.

Jamieson D, Uyeki T, Callahan WM, Meaney-Delman D, Rasmussen SA. What Obstetrician-Gynecologists Should Know About Ebola: A Perspective From the Centers for Disease Control and Prevention. *Obstet Gynecol*. 2014, *in press*.

Jin Z, Smith LK, Rajwanshi VK, Kim B, Deval J. The ambiguous base-pairing and high substrate efficiency of T-705 (Favipiravir) Ribofuranosyl 5'-triphosphate towards influenza A virus polymerase. *PloS one*. 2013;8(7):e68347.

Johnson KM. Ebola hemorrhagic fever in Zaire, 1976. *Bull World Health Organ* 1978; 56: 271–293. http://whqlibdoc.who.int/bulletin/1978/Vol56-No2/bulletin_1978_56(2)_271-293.pdf

Kiso M, Takahashi K, Sakai-Tagawa Y, Shinya K, Sakabe S, Le QM, Ozawa M, Furuta Y, Kawaoka Y. T-705 (favipiravir) activity against lethal H5N1 influenza A viruses. *Proc Natl Acad Sci U S A*. 2010;107(2):882-7.

Ksiazek TG, Rollin PE, Williams AJ, Bressler DS, Martin ML, Swanepoel R, Burt FJ, Leman PA, Khan AS, Rowe AK, Mukunu R, Sanchez A, Peters CJ. Clinical virology of Ebola hemorrhagic fever (EHF): virus, virus antigen, and IgG and IgM antibody findings among EHF patients in Kikwit, Democratic Republic of the Congo, 1995. *J Infect Dis*. 1999;179 Suppl 1:S177-87.

Mendenhall M, Russell A, Juelich T, Messina EL, Smee DF, Freiberg AN, Holbrook MR, Furuta Y, de la Torre JC, Nunberg JH, Gowen BB. T-705 (favipiravir) inhibition of arenavirus replication in cell culture. *Antimicrob. Agents Chemother*. 2011;55(2):782-7.

Mendenhall M, Russell A, Smee DF, Hall JO, Skirpstunas R, Furuta Y, Gowen BB. Effective oral favipiravir (T-705) therapy initiated after the onset of clinical disease in a model of arenavirus hemorrhagic Fever. *PLoS Negl Trop Dis*. 2011;5:e1342.

Mentré F, Taburet AM, Guedj J, Anglaret X, Keita S, de Lambalerie X, Malvy D. Choice of dosage regimen for the first evaluation of favipiravir in humans infected with Ebola virus. *Lancet Infect Dis*. 2014*, in press*.

Morrey JD, Taro BS, Siddharthan V, Wang H, Smee DF, Christensen AJ, Furuta Y. Efficacy of orally administered T-705 pyrazine analog on lethal West Nile virus infection in rodents. *Antiviral Res*, 2008, 80, 377–379.

Mupapa K, Mukundu W, Bwaka MA, Kipasa M, De Roo A, Kuvula K, Kibadi K, Massamba M, Ndaberey D, Colebunders R, Muyembe-Tamfum JJ. Ebola hemorrhagic fever and pregnancy. *J Infect Dis*. 1999;179 Suppl 1:S11-2.

OCHA report; United Nations Office for the coordination of Humanitarian Affairs, WHO.

Oestereich L, Lüdtke A, Wurr S, Rieger T, Muñoz-Fontela C, Günther S. Successful treatment of advanced Ebola virus infection with T-705 (favipiravir) in a small animal model. *Antiviral Res*, 2014, 105:17-21.

Olinger GG Jr, Pettitt J, Kim D, Working C, Bohorov O, Bratcher B, Hiatt E, Hume SD, Johnson AK, Morton J, Pauly M, Whaley KJ, Lear CM, Biggins JE, Scully C, Hensley L, Zeitlin L. Delayed treatment of Ebola virus infection with plant-derived monoclonal antibodies provides protection in rhesus macaques. *Proc Natl Acad Sci U S A*. 2012;109:18030-5.

Peacock G, Uyeki TM, Rasmussen SA. Ebola Virus Disease and Children: What Pediatric Health Care Professionals Need to Know. *JAMA Pediatr*. 2014, *in press.*

Qiu X, Wong G, Audet J, Bello A, Fernando L, Alimonti JB, et al. Reversion of advanced Ebola virus disease in nonhuman primates with ZMapp. *Nature*. 2014;514:47-53.

Rocha-Pereira J, Jochmans D, Dallmeier K., Leyssen P, Nascimento MS, Neyts J. Favipiravir (T-705) inhibits in vitro norovirus replication. *Biochem. Biophys. Res. Commun*. 2012, 424, 777–780.

Safronetz D, Falzarano D, Scott DP, Furuta Y, Feldmann H, Gowen BB. Antiviral efficacy of favipiravir against two prominent etiological agents of hantavirus pulmonary syndrome. *Antimicrob. Agents Chemother*. 2013, 57, 4673–4680.

Schieffelin JS, Shaffer JG, Goba A, Gbakie M, Gire SK, Colubri A, Sealfon RS, Kanneh L, Moigboi A, Momoh M, Fullah M, Moses LM, Brown BL, Andersen KG, Winnicki S, Schaffner SF, Park DJ, Yozwiak NL, Jiang PP, Kargbo D, Jalloh S, Fonnie M, Sinnah V, French I, Kovoma A, Kamara FK, Tucker V, Konuwa E, Sellu J, Mustapha I, Foday M, Yillah M, Kanneh F, Saffa S, Massally JL, Boisen ML, Branco LM, Vandi MA, Grant DS, Happi C, Gevao SM, Fletcher TE, Fowler RA, Bausch DG, Sabeti PC, Khan SH, Garry RF; KGH Lassa Fever Program; Viral Hemorrhagic Fever Consortium; WHO Clinical Response Team. Clinical illness and outcomes in patients with Ebola in Sierra Leone. *N Engl J Med*. 2014;371(22):2092-100.

Sleeman K, Mishin VP, Deyde VM, Furuta Y, Klimov AI, Gubareva LV. In vitro antiviral activity of favipiravir (T-705) against drug-resistant influenza and 2009 A(H1N1) viruses. *Antimicrob. Agents Chemother*. 2010;54(6):2517-24.

Smee DF, Hurst BL, Wong MH, Bailey KW, Tarbet EB, Morrey JD, Furuta Y. Effects of the combination of favipiravir (T-705) and oseltamivir on influenza A virus infections in mice. *Antimicrob. Agents Chemother*. 2010;54(1):126-33.

Smee DF, Hurst BL, Egawa H, Takahashi K, Kadota T, Furuta Y. Intracellular metabolism of favipiravir (T-705) in uninfected and influenza A (H5N1) virus-infected cells. *J Antimicrob Chemother*. 2009;64(4):741-6.

Smee DF, Tarbet EB, Furuta Y, Morrey JD, Barnard DL. Synergistic combinations of favipiravir and oseltamivir against wild-type pandemic and oseltamivir-resistant influenza A virus infections in mice. *Future virology*. 2013;8(11):1085-94.

Smither SJ, Eastaugh LS, Steward JA, Nelson M, Lenk RP, Lever MS. Post-exposure efficacy of oral T-705 (Favipiravir) against inhalational Ebola virus infection in a mouse model. *Antiviral Res*. 2014;104:153-5.

Tarbet EB, Maekawa M, Furuta Y, Babu YS, Morrey JD, Smee DF. Combinations of favipiravir and peramivir for the treatment of pandemic influenza A/California/04/2009 (H1N1) virus infections in mice. *Antiviral research*. 2012;94(1):103-10

Tayama Y, Miyake K, Sugihara K, Kitamura S, Kobayashi M, Morita S, Ohta S, Kihira K. Developmental changes of aldehyde oxidase activity in young Japanese children. *Clin Pharmacol Ther*. 2007;81(4):567-72.

Tayama Y, Sugihara K, Sanoh S, Miyake K, Kitamura S, Ohta S. Developmental changes of aldehyde oxidase activity and protein expression in human liver cytosol. *Drug Metab Pharmacokinet*. 2012;27(5):543-7.

UN, Global Response to Ebola Crisis, 18 sept 2014.

Unprecedented number of medical staff infected with Ebola. Geneva: World Health Organization, August 25, 2014e (http:// www.who.int/mediacentre/news/ebola/25-august-2014/en).

Warren TK, Wells J, Panchal RG, Stuthman KS, Garza NL, Van Tongeren SA, Dong L, Retterer CJ, Eaton BP, Pegoraro G, Honnold S, Bantia S, Kotian P, Chen X, Taubenheim BR, Welch LS, Minning DM, Babu YS, Sheridan WP, Bavari S. Protection against filovirus diseases by a novel broad-spectrum nucleoside analogue BCX4430. *Nature*. 2014;508:402-5.

WHO Ebola Response Team. Ebola Virus Disease in West Africa The First 9 Months of the Epidemic and Forward Projections. *N Engl J Med.* 2014;371(16):1481-95.

World Health Organization. Ebola Response Roadmap update: 3 October 2014d (http://apps.who.int/iris/bitstream/10665/134449/1/roadmapupdate22sept14_eng.pdf?ua=1).
