# Supplementary material for: Experimental Treatment with Favipiravir for Ebola Virus Disease (the JIKI Trial): A Historically Controlled, Single-Arm Proof-of-Concept Trial in Guinea
Source: PLoS Med. 2016 Mar 1;13(3):e1001967. doi: 10.1371/journal.pmed.1001967 (PMC4773183; doi:10.1371/journal.pmed.1001967)
Supplement: S3 Text — (PDF) [file pmed.1001967.s011.pdf]

# JIKI TRIAL

## Information sheets and consent forms

**Table of contents:**


- **Information Sheet, adults, original version (French)**
- **Consent Form, adults, original version (French)**
- **Information Sheet, parental, original version (French)**
- **Consent Form, parental, original version (French)**
- **Information Sheet, parental, original version (French)**
- **Information Sheet, children, original version (French)**

- **Information sheet, adults, translation (English)**
- **Consent Form, adults, translation (English)**
- **Information sheet, parental, translation (English)**
- **Consent Form, parental, translation (English)**
- **Information sheet, parental, translation (English)**
- **Information sheet, children, translation (English)**

# Notice d'information aux adultes auxquelles est proposée la participation à l'essai JIKI

Madame ou Monsieur,

Le médecin que vous allez voir va vous proposer de participer à une étude intitulée « **Efficacité du favipiravir pour réduire la mortalité chez les personnes infectées par le virus Ebola en Guinée** » (Essai JIKI).

Cette étude portée par l'INSERM est conduite dans le cadre d'un programme collaboratif avec le Ministère de la Santé de Guinée et Médecins Sans Frontières (MSF). Elle est dirigée par le Docteur Sakoba Keita, coordonnateur de la lutte contre le virus Ebola en Guinée, et le Professeur Denis Malvy, Professeur de Maladies Infectieuses en France.

**Pourquoi vous propose-t-on de participer ?**

On vous propose de participer à cette étude parce que le test sanguin que vous venez de faire montre que vous avez une infection par le virus Ebola.

**Qu'est ce que « l'infection par le virus Ebola » ?**

Le virus Ebola est un microbe actuellement très fréquent en Guinée. C'est une maladie contagieuse, qui s'attrape par contact direct avec une personne malade. Quand on a été en contact avec une personne malade et qu'on devient infecté par le virus, on développe des symptômes comme, par exemple, ceux que vous avez actuellement: fièvre, fatigue, maux de tête, maux de ventre, nausées, vomissements, diarrhée.

**Quels sont les traitements actuels du Virus Ebola ?**

Actuellement, quand on est infecté par le virus, on reçoit un traitement pour soulager les symptômes: médicaments contre la douleur, produits nutritifs, médicaments contre les infections (comme le paludisme), médicaments contre la diarrhée, les vomissements, ou la déshydratation. On est également hospitalisé dans une partie isolée du centre de soins parce que, pendant quelques jours, on est soi-même contagieux et si on a des contacts avec d'autres personnes on risque de les contaminer.

La maladie à Ebola est une maladie grave dont on peut mourir. Tous ces traitements diminuent le risque de mourir, mais comme ils n'agissent pas directement contre le virus lui-même, ils ne sont pas aussi efficaces qu'on le souhaiterait. Il faut donc tenter de trouver des nouveaux médicaments qui agissent contre le virus, et qui pourraient empêcher la maladie de devenir grave et d'en mourir.

**Qu'est ce qu'on vous propose de faire dans cette étude ?**

Dans cette étude, on veut essayer un médicament qui s'appelle le favipiravir. C'est un médicament qui est efficace contre la grippe. On pense qu'avec des doses plus fortes, il pourrait aussi être efficace contre le virus Ebola, mais on n'en est pas sûr. Dans cette étude, on voudrait le tester.

**Comment va se dérouler l'étude ?**

Le favipiravir se donne en comprimés. Trois fois par jour le premier jour : première dose de 12 comprimés puis 12 comprimés 8 heures après et 6 comprimés 16 heures après. Ensuite la dose est de 6 comprimés deux fois par jour pendant les 9 jours suivants.

Pendant votre hospitalisation, on vous fera une prise de sang le premier jour, le $2^{eme}$ jour, le $4^{eme}$ jour, à la fin des symptômes, le $13^{eme}$ jour et le $30^{eme}$ jour pour rechercher la présence du virus dans le sang.

A chaque prise de sang, un petit peu de sang sera pris en plus dans un petit tube de 4 ml. Ces tubes ne contiendront pas de nom, uniquement un numéro et ils seront stockés et conservés au congélateur pour être analysés plus tard en France dans le laboratoire hautement sécurisé de Lyon. Un tel laboratoire n'existe pas en Guinée. Ces analyses comprendront des mesures de la quantité de virus dans le sang, des mesures de la quantité de médicament dans le sang. Les résultats de ces analyses n'auront aucune conséquence sur votre suivi médical et sur votre traitement. Cela servira également pour la recherche sur le virus Ebola, l'étude de nouveaux tests pour identifier le virus de l'Ebola, l'étude de nouveaux traitements, et l'étude des effets de l'infection à Ebola sur l'humain.

**Quels sont les avantages et les risques de participer à l'étude ?**

Comme il n'y a jamais eu d'étude du favipiravir chez des personnes infectées par Ebola, on ne sait pas si ce médicament va être efficace ou pas. Si vous acceptez de participer, on espère que vous irez mieux, mais on ne peut pas vous le promettre. Par ailleurs, comme tout médicament, le favipiravir peut avoir des risques (effets secondaires), comme la diarrhée, et l'atteinte du foie ou des articulations. On pense qu'il est bien toléré, puisque les nombreuses personnes qui l'ont pris auparavant contre la grippe n'ont pas eu de problème important. Cependant, dans notre étude, on va le donner à des doses plus élevées que les doses qu'on a utilisées auparavant contre la grippe; il est donc possible qu'on découvre des effets secondaires qu'on n'attend-pas.

**Est-ce que je peux refuser ?**

Oui, vous êtes totalement libre d'accepter ou de refuser de participer à l'étude, donc de prendre ou pas le favipiravir. Si vous refusez, vous n'avez pas à expliquer pourquoi, et cela n'aura aucune conséquence sur les soins qui vous seront donnés et sur les relations avec les médecins. De même, si vous acceptez de participer vous pourrez demander à tout moment d'arrêter votre participation, donc d'arrêter le médicament, tout en continuant à être traité comme les personnes qui restent dans l'étude.

Si vous acceptez de participer, il vous sera demandé de donner votre « consentement ». L'étude pourra alors commencer immédiatement pour vous.

Vous pouvez dès maintenant poser toutes les questions complémentaires que vous souhaitez à la personne qui vous a expliqué l'étude.

Vous pouvez également vous désengager à n'importe quel moment de l'étude. Dans ce cas vous recevrez les mêmes soins que les personnes qui ne participent pas à l'étude et dans les meilleures conditions. Vos données ne seront plus inclues dans l'étude.

**Que va-t-il se passer en fin d'étude ?**

Quand les signes de la maladie auront disparu, on vous fera une dernière prise de sang pour confirmer que vous êtes guéris, puis vous pourrez sortir de l'hôpital. On vous expliquera alors exactement les précautions qu'il faut prendre, et on vous proposera de revenir une fois en consultation environ deux semaines plus tard pour s'assurer que tout va bien et pour faire une prise de sang.

A la fin de l'étude, vous serez également invité à une réunion d'information sur les résultats de cette étude pour que vous puissiez connaître exactement la réponse aux questions auxquelles votre participation aura permis de répondre.

**Confidentialité, utilisation des résultats, et dispositions légales**

Cette étude a été autorisée par le Comité National d'Éthique et par le Ministre de la Santé de la Guinée. Le Comité d'Évaluation Éthique de l'Inserm en France et le Comité d'Éthique de Médecins Sans Frontières ont examiné l'étude et donné un avis favorable. En cas de dommage vous avez la

garantie d'être protégé par l'assurance responsabilité civile qui a été souscrite par l'Inserm qui est le promoteur de l'étude.

Toutes les données vous concernant, c'est-à-dire les questionnaires remplis par l'équipe médicale à chacune de vos visites, et les résultats de vos prises de sang, resteront confidentiels et ne pourront être consultés que par l'équipe médicale qui s'occupe de la recherche. Il n'y aura aucune utilisation commerciale de vos données. Les prélèvements sanquins seront conservés 10 ans en France. Les données médicales seront conservées 10 ans en France et en Guinée. L'avis L'avis des comités d'éthique qui ont approuvé la présente étude sera systématiquement redemandé en cas de changement de la finalité de la recherche ou de changement de la durée de conservation des prélèvements.

Ces données seront informatisées et « non identifiantes », c'est-à-dire que votre nom ni aucune autre donnée permettant de vous identifier ne figurera dans aucun des documents papier et aucun des recueils informatiques renseignés pour l'essai. Ces données seront identifiées par un code unique composé de lettre et de chiffres.

Ces données seront analysées en France et en Guinée. Vous pouvez à tout moment demander à votre médecin les informations qui se trouvent dans votre dossier médical, y compris les informations recueillies dans le cadre de cet essai, et demander à ce que les informations qui s'y trouvent soient rectifiées si elles ne sont pas exactes.

# Formulaire de consentement pour un patient adulte

**Je soussigné (e) …………………………………….., déclare avoir lu ou qu'il m'a été lu la fiche d'information jointe à ce formulaire et avoir clairement compris les objectifs, avantages et inconvénients de l'étude.**

**J'ai pu discuter de cette étude avec le médecin qui a proposé ma participation. Il a répondu à toutes mes questions de manière claire. Les risques et avantages m'ont été expliqués.**

**Je comprends que ma participation à cette étude est volontaire et que je peux choisir de refuser de participer sans avoir à en expliquer les raisons.**

**Je comprends aussi que certains échantillons de sang seront congelés et conservés pour analyses futures.**

**Je donne mon accord pour participer à cette étude.**

**Signature (ou empreinte digitale) du participant ………………………..,**

**Date ……………………..,**

**Signature du Témoin éventuel du consentement oral ………………………..,**

**Date ……………………..,**

**Je soussigné Dr……………………………………….., atteste avoir expliqué les détails pertinents de cet essai au participant nommé ci-dessus. Je me suis assuré que le participant a compris et a donné son consentement en toute connaissance de cause.**

**Je promets de respecter tous les termes et conditions mentionnés dans ce formulaire de consentement, de garder une confidentialité totale et de respecter les droits et libertés de l'individu aussi bien que les exigences du travail scientifique.**

**Signature de l'investigateur……………………..,** **Date ……………………..,**

# Notice d’information aux parents (ou titulaires de l’autorité parentale) d’un patient mineur auquel est proposée la participation à l’essai JIKI

Madame ou Monsieur,

Le médecin que vous allez voir va vous proposer que votre enfant participe à une étude intitulée « **Efficacité du favipiravir pour réduire la mortalité chez les personnes infectées par le virus Ebola en Guinée** » (Essai JIKI).

Cette étude portée par l’INSERM est conduite dans le cadre d’un programme collaboratif avec le Ministère de la Santé de Guinée et Médecins Sans Frontières (MSF). Elle est dirigée par le Docteur Sakoba Keita, coordonnateur de la lutte contre le virus Ebola en Guinée, et le Professeur Denis Malvy, Professeur de Maladies Infectieuses en France.

**Pourquoi propose-t-on que votre enfant participe ?**

On vous propose que votre enfant participe à cette étude parce qu’il est malade. Sa maladie est due à une infection par le virus Ebola

**Qu’est ce que « l’infection par le virus Ebola »** ?

Le virus Ebola est un microbe actuellement très fréquent en Guinée. C’est une maladie contagieuse, qui s’attrape par contact direct avec une personne malade. Quand on a été en contact avec une personne malade et qu’on devient infecté par le virus, on développe des symptômes comme, par exemple, ceux que vous avez actuellement : fièvre, fatigue, maux de tête, maux de ventre, nausées, vomissements, diarrhée.

**Quels sont les traitements actuels du Virus Ebola ?**

Actuellement, quand on est infecté par le virus, on reçoit un traitement pour soulager les symptômes : médicaments contre la douleur, produits nutritifs, médicaments contre les infections (comme le paludisme), médicaments contre la diarrhée, les vomissements, ou la déshydratation. On est également hospitalisé dans une partie isolée du centre de soins parce que, pendant quelques jours, on est soi-même contagieux et si on a des contacts avec d’autres personnes on risque de les contaminer.

La maladie à Ebola est une maladie grave dont on peut mourir. Tous ces traitements diminuent le risque de mourir, mais comme ils n’agissent pas directement contre le virus lui-même, ils ne sont pas aussi efficaces qu’on le souhaiterait. Il faut donc tenter de trouver des nouveaux médicaments qui agissent contre le virus, et qui pourraient empêcher la maladie de devenir grave et d’en mourir.

**Qu’est ce qu’on vous propose de faire dans cette étude ?**

Dans cette étude, on veut essayer un médicament qui s’appelle le favipiravir. C’est un médicament qui est efficace contre la grippe. On pense, qu’avec des doses plus fortes, il pourrait aussi être efficace contre le virus Ebola, mais on n'en est pas sûr. Dans cette étude, on voudrait le tester chez environ 45 enfants.

**Comment va se dérouler l’étude?**

Le favipiravir se donne en comprimés, dont le nombre sera adapté au poids de votre enfant. Si votre enfant est trop jeune ou trop faible pour arriver à avaler les comprimés, ils pourront être écrasés et mélanger à de la nourriture ou des boissons pour qu’ils soient plus facile de les avaler. Au total, le traitement va durer 10 jours.

Votre enfant sera hospitalisé(e) dans la zone d'isolement, il(elle) recevra tous les soins habituels dont on vous a parlé plus haut (médicaments de la nutrition et de la réhydratation, antidouleurs, etc.), et il(elle) prendra en plus le favipiravir pendant 10 jours.

Pendant son hospitalisation, on fera une prise de sang à votre enfant le premier jour, le 2$^{eme}$ jour, le 4$^{eme}$ jour, à la fin des symptômes, le 13$^{eme}$ jour et le 30$^{eme}$ jour, pour rechercher la présence du virus dans le sang.

A chaque prise de sang, un petit peu de sang sera pris en plus dans un petit tube. Ces tubes ne contiendront pas de nom, uniquement un numéro et ils seront stockés et conservés au congélateur pour être analysés plus tard en France dans le laboratoire hautement sécurisé de Lyon. Un tel laboratoire n'existe pas en Guinée. Ces analyses comprendront des mesures de la quantité de virus dans le sang et des mesures de la quantité de médicament dans le sang. Les résultats de ces analyses n'auront aucune conséquence sur le suivi médical et sur le traitement de votre enfant. Cela servira également pour la recherche sur le virus Ebola, l'étude de nouveaux tests pour identifier le virus de l'Ebola, l'étude de nouveaux traitements, et l'étude des effets de l'infection à Ebola sur l'humain. En aucun cas ces échantillons ne pourront être utilisés à des fins comerciales. Ils seront détruits au bout de 10 ans s'ils n'ont pas servi.

**Quels sont les avantages et les risques de participer à l'étude?**

Comme il n'y a jamais eu d'étude du favipiravir chez des personnes infectées par Ebola, on ne sait pas si ce médicament va être efficace ou pas. Si vous acceptez que votre enfant participe, on espère qu'il(elle) ira mieux, mais on ne peut pas vous le promettre. Par ailleurs, comme tout médicament, le favipiravir peut avoir des risques (effets secondaires) comme la diarrhée et l'atteinte du foie ou des articulations. On pense qu'il est bien toléré, puisque les nombreux adultes qui l'ont pris auparavant contre la grippe n'ont pas eu de problème important. Cependant, dans notre étude, on va le donner à des doses plus élevées que les doses qu'on a utilisées auparavant contre la grippe. De plus, le favipiravir n'a encore jamais été prescrit à des enfants jusqu'à présent ; il est donc possible qu'on découvre des effets secondaires qu'on n'attend pas.

**Est-ce que je peux refuser ?**

Oui, vous êtes totalement libre d'accepter ou de refuser que votre enfant participe à l'étude, donc qu'il reçoive ou pas le favipiravir. Si vous refusez, vous n'avez pas à expliquer pourquoi, et cela n'aura aucune conséquence sur les soins qui seront donnés à votre enfant et sur les relations avec votre médecin. De même, si vous acceptez que votre enfant participe, vous pourrez demander à tout moment d'arrêter sa participation, donc d'arrêter le médicament, tout en continuant à ce qu'il(elle) soit traité(e) comme les personnes qui restent dans l'étude.

Si vous acceptez que votre enfant participe, il vous sera demandé de signer le « formulaire de consentement ». Selon l'âge de votre enfant, il (elle) pourra également signer ce formulaire en même temps que vous s'il (elle) le souhaite. L'étude pourra alors commencer immédiatement pour votre enfant.

Vous pouvez dès maintenant poser toutes les questions complémentaires que vous souhaitez à la personne qui vous a expliqué l'étude.

Vous pouvez également désengager à n'importe quel moment de l'étude votre enfant. Dans ce cas votre enfant recevra les mêmes soins que les personnes qui ne participent pas à l'étude et dans les meilleures conditions. Les données de votre enfant ne seront plus inclues dans l'étude. Votre enfant peut également de lui-même se désengager à n'importe quel moment de l'étude.

**Que va-t-il se passer en fin d'étude ?**

Quand les signes de la maladie auront disparu, on fera à votre enfant une dernière prise de sang pour confirmer qu'il(elle) est guéri(e), puis il(elle) pourra sortir de l'hôpital. On vous expliquera alors exactement les précautions qu'il faut prendre, et on vous proposera de revenir une fois en consultation environ deux semaines plus tard pour s'assurer que tout va bien et pour faire une prise de sang.

A la fin de l'étude, vous et votre enfant serez également invité à une réunion d'information sur les résultats de cette étude pour que vous puissiez connaître exactement la réponse aux questions auxquelles sa participation aura permis de répondre.

**Confidentialité, utilisation des résultats, et dispositions légales**

Cette étude a été autorisée par le Comité National d'Éthique et par le Ministre de la Santé de la Guinée. Le Comité d'Évaluation Éthique de l'Inserm en France et le Comité d'Éthique de Médecins Sans Frontières ont a examiné l'étude et ont donné un avis favorable. En cas de dommage vous avez la garantie que votre enfant est protégé par l'assurance responsabilité civile qui a été souscrite par l'Inserm qui est le promoteur de l'étude.

Toutes les données concernant votre enfant, c'est-à-dire les questionnaires remplis par l'équipe médicale à chacune de ses visites, et les résultats de ses prises de sang, resteront confidentiels et ne pourront être consultés que par l'équipe médicale qui s'occupe de la recherche. Il n'y aura aucune utilisation commerciale de ses données. Les prélèvements sanquins seront conservés 10 ans en France. Les données médicales seront conservées 10 ans en France et en Guinée. L'avis des comités d'éthique qui ont approuvé la présente étude sera systématiquement redemandé en cas de changement de la finalité de la recherche ou de changement de la durée de conservation des prélèvements.

Ces données seront informatisées et « non identifiantes », c'est-à-dire que ni votre nom ni celui de votre enfant ni aucune autre donnée permettant de vous identifier ne figureront dans aucun des documents papier et aucun des recueils informatiques renseignés pour l'essai. Ces données seront identifiées par un code unique composé de lettre et de chiffres.

Ces données seront analysées en France et en Côte d'Ivoire. Vous pouvez à tout moment demander au médecin de votre enfant les informations qui se trouvent dans son dossier médical, y compris les informations recueillies dans le cadre de cet essai, et demander à ce que les informations qui s'y trouvent soient rectifiées si elles ne sont pas exactes.

# Formulaire de consentement pour les parents (ou titulaires de l'autorité parentale) d'un patient mineur

**Je soussigné (e) ……………………………………………, père / mère / titulaire de l'autorité parentale de l'enfant ……………………………………………, déclare avoir lu ou qu'il m'a été lue la fiche d'information jointe à ce formulaire et avoir clairement compris les objectifs, avantages et inconvénients de l'étude.**

**J'ai pu poser toutes les questions et discuter de ce projet de recherche avec le médecin qui a proposé ma participation, dont le nom et la signature sont indiqués ci-dessous, aussi bien qu'avec le personnel de l'étude. Ils ont répondu à toutes mes questions de manière claire. Les risques et avantages m'ont été expliqués.**

**Je comprends que la participation de mon enfant à cet essai clinique est volontaire, que je peux choisir de refuser qu'il (elle) y participe et que, si j'accepte, je peux retirer mon consentement à tout moment sans aucune pénalité ou désavantage pour moi ni mon enfant sans avoir à en expliquer les raisons.**

**Je comprends aussi que certains échantillons de sang seront congelés et conservés pour analyses futures.**

**Je donne mon accord pour que mon enfant participe à cette étude.**

**En signant ce formulaire de consentement, je ne renonce à aucun de mes droits légaux.**

**Signature (ou empreinte digitale) des parents**……………………………,

**Date** ………………………,

**Signature du Témoin éventuel du consentement oral**……………………………,

**Date** ………………………,

**Signature (ou empreinte digitale) du patient mineur (facultatif)**……………………………,

**Date** ………………………,

**Je soussigné Dr……………………………………………, atteste avoir expliqué les détails pertinents de cet essai au père (ou mère ou titulaire de l'autorité parentale) de l'enfant nommé ci-dessus. Je me suis assuré que celui-ci (celle-ci) a compris et a donné son consentement en toute connaissance de cause.**

**Je promets de respecter tous les termes et conditions mentionnés dans ce formulaire de consentement, de garder une confidentialité totale et de respecter les droits et libertés de l'individu aussi bien que les exigences du travail scientifique.**

**Signature de l'investigateur**………………………, **Date** ………………………,

# Notice d'information aux mineurs auxquels est proposée la participation à l'essai JIKI

Le médecin que tu vas voir va te proposer participer à une étude intitulée « **Efficacité du favipiravir pour réduire la mortalité chez les personnes infectées par le virus Ebola en Guinée** » (Essai JIKI). On te propose de participer à cette étude parce qu'on pense que tu as une infection par le virus Ebola.

**<u>Qu'est ce que « l'infection par le virus Ebola »</u> ?**

Le virus Ebola est un microbe actuellement très fréquent en Guinée. C'est une maladie contagieuse, qui s'attrape par contact avec une personne malade. Quand on devient infecté par le virus, on développe des symptômes comme, par exemple, ceux que tu as actuellement : fièvre, fatigue, maux de tête, maux de ventre, nausées, vomissements, diarrhée.

**<u>Quels sont les traitements actuels du Virus Ebola ?</u>**

Actuellement, quand on est infecté par le virus, on reçoit un traitement pour soulager les symptômes. Ces traitements diminuent le risque de mourir, mais comme ils n'agissent pas directement contre le virus lui-même, ils ne sont pas aussi efficaces qu'on le souhaiterait. Il faut donc tenter de trouver des nouveaux médicaments qui agissent contre le virus, et qui pourraient empêcher la maladie de devenir grave et d'en mourir.

**<u>Comment va se dérouler l'étude?</u>**

Dans cette étude, on veut essayer un médicament qui s'appelle le favipiravir. On pense que ce médicament pourrait aussi être efficace contre le virus Ebola, mais on n'en est pas sûr.

Tu seras hospitalisé(e) dans la zone d'isolement et tu prendras des comprimés de favipiravir pendant 10 jours. Ces comprimés seront écrasés et mélangés à de la nourriture ou des boissons pour qu'ils soient plus facile de les avaler. Pendant ton hospitalisation, on fera plusieurs prises de sang pour rechercher la présence du virus dans le sang.

**<u>Quels sont les avantages et les inconvénients de participer à l'étude?</u>**

Comme il n'y a jamais eu d'étude du favipiravir chez des personnes infectées par Ebola, on ne sait pas si ce médicament va être efficace ou pas. Par ailleurs, comme tout médicament, le favipiravir peut avoir des inconvénients (effets secondaires). On pense qu'il est bien toléré mais on n'en est pas sûr.

**<u>Est-ce que je peux refuser ?</u>**

Oui, tu es totalement libre d'accepter ou de refuser de participer à l'étude, donc de recevoir ou pas le favipiravir. Si tu refuses, cela n'aura aucune conséquence sur les soins qui te seront donnés ni sur les relations avec ton médecin. A tout moment, tu peux demander d'arrêter ta participation, donc d'arrêter le médicament.

Si tu acceptes de participer, tu peux signer avec tes parents le « formulaire de consentement ». Tu peux dès maintenant poser toutes les questions complémentaires que tu veux à la personne qui t'a expliqué l'étude.

**<u>Que va-t-il se passer en fin d'étude ?</u>**

Quand les signes de la maladie auront disparu, on te fera une dernière prise de sang pour confirmer que tu es guéri(e), puis tu pourras sortir de l'hôpital. On t'expliquera alors exactement les précautions qu'il faut prendre, et on te proposera de revenir une fois en consultation environ deux semaines plus tard pour s'assurer que tout va bien et pour faire une prise de sang.

A la fin de l'étude, tu seras invité avec tes parents à une réunion d'information sur les résultats de cette étude.

# Information sheet for potential adults to participate in JIKI trial

Dear Sir or Madam,

The physician you are about to speak with will invite you to participate in a study entitled « **Efficacy of favipiravir to reduce mortality in patients infected with Ebola virus in Guinea** » (JIKI trial).

INSERM is the sponsor of this study and conducted as part of collaboration between the Ministry of Health of Guinea and Médecins Sans Frontières/Doctors Without Borders (MSF). It is headed by Dr. Sakoba Keita, coordinator of the fight against Ebola virus in Guinea, and Professor Denis Malvy, Professor of Infectious Diseases in France.

**Why are we inviting you to participate?**

We are inviting you to participate in this study because the result of the Ebola virus confirmatory test is positive.

**What is "Ebola virus disease"?**

Ebola virus is a microbe that is currently very common in Guinea. It is a contagious disease that spreads through direct contact with an infected person. When a person has been in contact with an infected person and becomes infected with the virus, he/she develops symptoms such as those you are currently showing: fever, fatigue, headaches, stomachaches, nausea, vomiting and diarrhea.

**What treatments are currently available for Ebola virus?**

Currently, when one is infected with the virus, one receives a treatment to relieve the symptoms: painkillers, nutrients, drugs against infections (such as malaria), diarrhea, vomiting or dehydration. The patient is hospitalized in an isolated room of the health center because he/she is contagious for a few days and is likely to contaminate people if he/she has contact with them.

Ebola disease is a serious disease which can kill. All these treatments reduce the risk of dying, but as they do not act directly against the virus itself, they are not as effective as expected. Therefore, we must try to find new drugs that act against the virus and which could prevent the disease from becoming severe and life-threatening.

**What is the purpose of this study?**

In this study, we want to try a drug called Favipiravir. This is drug which is effective against flu. We think it might also be effective against Ebola virus in higher doses, but we are not sure. We would like to test it in this study.

**How will the trial be implemented?**

The recommended dosage for Favipiravir in tablet form is on the first day 12 tablets, then 8 hours later 12 tablets then 8 hours later 6 tablets. For the next 9 days you will be given 6 tablets twice daily.

During your hospital stay, you will undergo a blood test on the second and fourth days, then at the end of the symptoms for the virus detection in the blood.

At every venous puncture an additional small amount of blood will be taken in one small tube of 7 ml. These tubes will not be named, only have a number and will be stored to be analysing later in France in the highly secure laboratory in Lyon. Such a laboratory does not exist in Guinea. These tests will measure the amount of virus in the blood and the drug concentration in the blood. The results of these

analyzes will not affect your medical care and your treatment. This library will be used for further research on the Ebola virus, tests new means of diagnosing Ebola infection, tests new treatments against Ebola and follow the effects of Ebola infection on the human body.

**What are the benefits and risks of participating in the study?**

Since there has never been a Favipiravir study in people infected with Ebola, we do not know whether this drug will be effective or not. If you agree to participate, we hope you will get better, but we cannot promise you. Furthermore, like any drug, Favipiravir can have risks (side effects), such as diarrhea, liver or joints damage. We think it is well tolerated, since many people who took it previously against influenza had no major problem. However, in our study, we will prescribe it at higher doses than those used previously against influenza; so, it is possible to discover side effects that are not expected.

**May I refuse?**

Yes, you are totally free to accept or refuse to participate in the study or to take Favipiravir or not. If you refuse, you do not have to explain why, and it will not affect the care given to you and your relationship with physicians. Similarly, if you agree to participate, you may request to withdraw your participation at any time, therefore discontinuing the drug while continuing to be treated as people who remain in the study.

If you agree to participate, you will be asked to give your "consent" meaning that you have immediately started the study.

You can now ask any question to the person who explained the study to you.

At any moment in time you can decide to stop your participation in the study. You will then stop receiving favipiravir, but you will still benefit from the standard of care as any other patient who is not on favipiravir receives. If you which, all your trial data can be removed from the trial data base.

**What will happen at the end of the study?**

When the signs of the disease will have disappeared, you will undergo a final blood test to confirm that you are healed at which time you will be discharged from the hospital. We will exactly explain to you what precautions to take and offer you an extended medical care. You will be asked to return to the treatment center two weeks later for a consultation to ensure that all is well and for a blood sample.

At the end of the study, you will also be invited to attend one briefing on the results of this study so that you can know the exact answers to the questions which your participation in the study will have helped to answer.

**Confidentiality, use of results, and legal measures**

This study was approved by the National Ethics Committee and the Minister of Health of Guinea. The Ethics Review Committee of Inserm in France and the Ethics Committee of Doctors Without Borders reviewed the study and gave a favorable opinion. In case of damage you are guaranteed to be protected by civil liability insurance has been taken out by Inserm the sponsor of the study.

All of your data, that is, the questionnaires completed by the medical team at each of your visits and your blood test results will remain confidential; only the medical team in charge of the research study will be entitled to analyze them. There will be no commercial use of your data. The samples will be stored 10 years in France. Medical data will be stored 10 years in France and in Guinea. Relevant ethics committees (ie the same as thos who approved the present study) will be systematically requested in case of change of the purpose of research or change of the time frame.

Your data will be computerized and "de-identified". In other words, your name or any other personal information that can identify you will not appear in any of the hard-copy documents and digital database of the study. These data will be identified by a unique identifier consisting of letters and numbers.

These data will be analyzed in France and Guinea. You can ask your physician the information in your medical records at any time including the data collected for this study and you can request him/her to correct the information in your records if they are not accurate.

# Consent form for an adult patient

**I undersigned …………………………………………, declare have read or make me read the information form provided with this consent form and I have understood the objectives, benefits and risks of the study.**

**I was able to discuss this study with the doctor who proposed my participation. He answered all my questions clearly. The risks and benefits have been explained to me.**

**I understand that my participation in this study is voluntary and that I can choose not to participate. If I agree, I can withdraw my consent at any time without penalty or disadvantage for my care without having to explain why.**

**I understand that some blood samples will be frozen and stored for future analysis.**

**I give my consent to participate in this study.**

**Signature (or fingerprint) of the patient**…………………………,

**Date** ……………………….,

**Signature of Witness (if oral consent)** …………………………,

**Date** ……………………….,

**I undersigned Dr**…………………………………………, **attest that, I explained the important information of the study to the patient named above. I ensured that the patient has understood and consented to knowingly**

**I promise to respect all terms and conditions mentioned in the consent form, to keep confidentiality and to respect the rights and freedoms of the patient as well as the requirements of scientific work.**

**Investigator signature** ……………………….,                                   **Date** ……………………….,

# Information sheet for parent (or holders of parental authority) of a minor patient to participate in JIKI trial

Dear Sir or Madam,

The doctor you are about to speak with will invite your child to participate in a study entitled « **Efficacy of favipiravir to reduce mortality in patients infected with Ebola virus in Guinea** » (JIKI trial).

INSERM is the sponsor of this study and conducted as part of a collaboration between the Ministry of Health of Guinea and Médecins Sans Frontières/Doctors Without Borders (MSF). It is headed by Dr. Sakoba Keita, coordinator of the fight against Ebola virus in Guinea, and Professor Denis Malvy, Professor of Infectious Diseases in France.

**Why are we inviting your child to participate?**

Your child is inviting to participate in this study because he (she) has Ebola virus disease.

**What is "Ebola virus disease"?**

Ebola virus is a microbe that is currently very common in Guinea. It is a contagious disease that spreads through direct contact with an infected person. When you have been in contact with an infected person and you become infected with the virus, you develop symptoms such as those your child is currently showing: fever, fatigue, headaches, stomachaches, nausea, vomiting and diarrhea.

**What treatments are currently available for Ebola virus?**

Currently, when one is infected with the virus, one receives a treatment to relieve the symptoms: painkillers, nutrients, drugs against infections (such as malaria), diarrhea, vomiting or dehydration. He/she is also hospitalized in an isolated room of the health center because he/she is contagious for a few days and is likely to contaminate people if he/she has contact with them.

Ebola disease is a serious disease which can kill. All these treatments reduce the risk of dying, but as they do not act directly against the virus itself, they are not as effective as expected. Therefore, we must try to find new drugs that act against the virus and which could prevent the disease from becoming severe and life-threatening.

**What is the purpose of this study?**

In this study, we want to try a drug called Favipiravir. This is a drug which is effective against flu. We think it might also be effective against Ebola virus in higher doses, but we are not sure. In this study, we would like to test on about 45 Children.

**How will the trial be implemented?**

The Favipiravir gives tablets whose numbers will be adapted to your child's weight.

If your child is too young or too small to swallow tablets, the tablets may be crushed and mixed with food or beverages. Overall, the treatment will last 10 days.

Your child will be hospitalized in the isolation area, he/she will receive all routine care (nutrition and hydration, painkillers, etc.), and in addition to Favipiravir during the 10 days.

During your hospital stay, your child will undergo a blood test on the second day and then at the end of the symptoms for the virus detection in the blood.

At every venous puncture an additional small amount of blood will be taken in one small tube. These tubes will not be named, only have a number and will be stored to be analysing later in France in the highly secure laboratory in Lyon. Such a laboratory does not exist in Guinea. These tests will measure the amount of virus in the blood and the drug concentration in the blood. The results of these analyzes will not affect the medical care and the treatment of your child. This library will be used for further research on the Ebola virus, tests new means of diagnosing Ebola infection, tests new treatments against Ebola and follow the effects of Ebola infection on the human body.

**What are the benefits and risks of participating in the study?**

Since there has never been a Favipiravir study in people infected with Ebola, we do not know whether this drug will be effective or not. If you agree that your child participate, we hope that your child will get better, but we cannot promise you. Furthermore, like any drug, Favipiravir can have risks (side effects) such as diarrhea, and liver or joints damage. We think it is well tolerated, since many people who took it previously against influenza had no major problem. However, in our study, we will prescribe it at higher doses than those used previously against influenza; so, it is possible to discover side effects that are not expected.

**May I refuse?**

Yes, you are totally free to accept or refuse that your child to participate in the study or to take Favipiravir or not. If you refuse, you do not have to explain why, and it will not affect the care given to your child and the relationship with the doctors. Similarly, if you agree that your child to participate, you may request at any time to withdraw its participation, therefore discontinuing the drug while continuing to be treated as people who remain in the study.

If you agree that your child to participate, you will be asked to sign a "consent form" meaning that you have immediately started the study. According to age of your child, he/she will also sign this consent form along with you if he/she wishes.

You can now ask any questions to the person who explained the study to you.

At any moment in time you can decide to stop the participation of your child in the study. Your child will then stop receiving favipiravir, but your child will still benefit from the standard of care as any other child who is not on favipiravir receives. If you which, all the trial data of your child can be removed from the trial data base. Your child may also itself disengage at any time of the study.

**What will happen at the end of study?**

When the signs of the disease will have disappeared, your child will undergo a final blood test to confirm that your child is healed and he/she could discharge from the hospital. We will exactly explain to you what precautions to take. You will be asked to return to the treatment center two weeks later for a consultation to ensure that all is well and for a blood sample.

At the end of the study, you will also be invited to attend one briefing on the results of this study so that you can know the exact answers to the questions which your participation in the study will have helped to answer.

**Confidentiality, use of results, and legal measures**

This study was approved by the National Ethics Committee and the Minister of Health of Guinea. The Ethics Review Committee of Inserm in France and the Ethics Committee of Doctors Without Borders reviewed the study and gave a favorable opinion. In case of damage you are guaranteed to be protected by civil liability insurance has been taken out by Inserm the sponsor of the study.

All of your child data, that is, the questionnaires completed by the medical team at each of your visits and your blood test results will remain confidential; only the medical team in charge of the research study will be entitled to analyze them. There will be no commercial use of your data. The samples will be stored 10 years in France. Medical data will be stored 10 years in France and in Guinea. Relevant ethics committees (ie the same as thos who approved the present study) will be systematically requested in case of change of the purpose of research or change of the time frame

Data will be computerized and "de-identified". In other words, the name of your child will not appear in any of the hard-copy documents and digital database of the study. These data will be identified by a unique identifier consisting of letters and numbers.

These data will be analyzed in France and Guinea. You can ask at doctor the information in medical records of your child at any time including the data collected for this study and request him/her to correct the information in your records if they are not accurate.

# Consent form for parents (or holders of parental authority) of a minor patient

**I undersigned ……………………………………., father / mother / holder of parental authority over the child……………………………………, declare have read or make me read the information form provided with this consent form and I have understood the objectives, benefits and risks of the study.**

**I was able to discuss this study with the doctor who my proposed this study. He answered all my questions clearly. He explains the risks and benefits at my.**

**I understand that the participation of my child in this study is voluntary. I can to refuse that he/she participates at study. If I agree, I can withdraw my consent at any time without penalty or disadvantage for me or my child without having to explain why.**

**I understand that some blood samples will be frozen and stored for future analysis.**

**I give my consent to that my child participates in this study.**

**Signature (or fingerprint) of the patient**………………………..,

**Date** ……………………..,

**Signature of Witness (if oral consent)** ………………………..,

**Date** ……………………..,

**Signature (or fingerprint) of the minor patient (facultative)**………………………..,

**Date** ……………………..,

**I undersigned Dr……………………………………….., attest that, I explained the important information of the study at father / mother / holder of parental authority of the child named above. I ensured that the patient has understood and consented to knowingly**

**I promise to respect all terms and conditions mentioned in the consent form, to keep confidentiality and to respect the rights and freedoms of the patient as well as the requirements of scientific work.**

**Investigator signature** ……………………..,                    **Date** ……………………..,

# Information sheet for potential minor patient to participate in JIKI trial

The doctor you are about to speak with will invite you to participate in a study entitled "Efficacy of Favipiravir to reduce mortality among **people infected with Ebola virus in Guinea**" (JIKI trial).

We are inviting you to participate in this study because you have Ebola virus disease.

**What is “Ebola virus disease”?**

Ebola virus is a microbe that is currently very common in Guinea. It is a contagious disease that spreads through direct contact with an infected person. When a person becomes infected with the virus, he/she develops symptoms such as those you are currently showing: fever, fatigue, headaches, stomachaches, nausea, vomiting and diarrhea

**What treatments are currently available for Ebola virus?**

Currently, when one is infected with the virus, one receives a treatment to relieve the symptoms. All these treatments reduce the risk of dying, but as they do not act directly against the virus itself, they are not as effective as expected. Therefore, we must try to find new drugs that act against the virus and which could prevent the disease from becoming severe and life-threatening.

**How will the trial be implemented?**

In this study, we want to try a drug called Favipiravir. We think this drug might also be effective against Ebola virus, but we are not sure.

You will be hospitalized in the isolated room and you will take favipiravir tablets for 10 days. The tablets will be grinded and mix with foods or beverages so that they will be easier to take. During your hospital stay, you will undergo many blood tests for the virus detection in the blood.

**What are the benefits and risks of participating in the study?**

Since there has never been a Favipiravir study in people infected with Ebola, we do not know whether this drug will be effective or not. Furthermore, like any drug, Favipiravir can have risks (side effects). We think it is well tolerated but we are not sure.

**May I refuse?**

Yes, you are totally free to accept or refuse to participate in the study or to receive Favipiravir or not. If you refuse, it will not affect the care given to you and your relationship with your physician. You may request to withdraw your participation at any time, therefore discontinuing the drug.

If you agree to participate, you and your parents will be asked to sign a "consent form".

You can now ask any question to the person who explained the study to you.

**What will happen at the end of the study?**

When the signs of the disease will have disappeared, you will undergo a final blood test to confirm that you are healed at which time you will be discharged from the hospital. We will exactly explain to you what precautions to take and offer you an extended medical care. You will be asked to return to the treatment center two weeks later for a consultation to ensure that all is well and for a blood sample.

At the end of the study, you and your parents will also be invited to attend one briefing on the results of this study.
